# Evaluating fine-tuned, embedding-based, and zero-shot models for aspect-based sentiment analysis in South Slavic news

**DOI:** 10.3389/frai.2026.1844418

**Published:** 2026-09-18

**Authors:** Nishan Chatterjee, Boshko Koloski, Antoine Doucet, Senja Pollak, Matthew Purver

**Affiliations:** 1 Jožef Stefan Institute, Ljubljana, Slovenia; 2 Jožef Stefan International Postgraduate School, Ljubljana, Slovenia; 3 University of La Rochelle, La Rochelle, France; 4 University of Ljubljana, Ljubljana, Slovenia; 5 Queen Mary University of London, London, United Kingdom

**Keywords:** aspect-based sentiment analysis, benchmark, large language models, learning-to-defer, media monitoring, South Slavic languages, targeted sentiment analysis

## Abstract

In the contemporary digital media landscape, the ability to automatically distill public opinion from a vast and continuous stream of information is highly important. Aspect-Based Sentiment Analysis (ABSA) offers this granular capability. In this work, we address a specific, industrially relevant formulation of this task, more formally known as document-level entity-targeted sentiment analysis (TSA) or entity-level sentiment analysis (ELSA), where the “aspects” are named entities such as companies and brands. However, its application to morphologically complex, less-resourced languages like those in the South Slavic family, particularly within the long-form news domain, remains a significant challenge. In this work, we introduce AspectBench, a new benchmark dataset for document-level ABSA, comprising real-world online news articles in Slovenian and Serbo-Croatian, designed to test generalization to unseen aspects, with the Serbo-Croatian portion being made publicly available. Using this benchmark, we conduct a comprehensive empirical study evaluating five distinct modeling paradigms, including lightweight document-embedding-based classifiers, fine-tuned multilingual and language-specific pretrained language models (PLMs), hierarchical attention networks (HANs), local large language models (LLMs) in zero and few-shot settings, and learning-to-defer (L2D) policies with multi-expert PLMs, complete LLM deferral, and confidence-gated selective LLM deferral using DSPy-calibrated prompts. Our experiments show that supervised expert models remain the most reliable foundation for this task, with Longformer, mDeBERTa-v3, mT5, language-specific encoders, and hierarchical models providing strong performance depending on language and class balance. Aspect masking is generally useful for supervised expert models, especially the PLM and HAN variants, but is not uniformly beneficial across all model families and evaluation views. Standalone local LLMs are not competitive, and complete LLM deferral is unstable when applied to every instance. However, selective LLM deferral is more promising because routing low-confidence expert predictions to the LLM can improve or stabilize performance while limiting unnecessary LLM calls. We additionally use minimum viable set analysis to show how performance, calibration, and uncertainty scale with annotation volume, providing practical guidance for future media-monitoring annotation campaigns on real-world datasets. Our work therefore fills a crucial gap for less-resourced ABSA and offers practical insights into the trade-offs between model complexity, data characteristics, and the development of robust, learning-to-defer systems for real-world media monitoring.

## 1 Introduction

The ability to automatically distill public opinion from a vast and continuous stream of online information is essential for modern media analysis. Sentiment analysis, therefore, is a key component in news media monitoring as it allows organizations to understand public opinion and react accordingly. By analyzing the emotional tone of media mentions, companies can identify potential crises, adjust their communication strategies, and even improve product development. It helps in measuring the effectiveness of public relations campaigns and understanding the public perception of a brand (Olukemi et al., 2024). However, news articles, blogs, and online content may contain multiple targets and a broad range of topics. Therefore, understanding not only the general sentiment but also the specific targets of that sentiment is the key to critical decision-making. Aspect-Based Sentiment Analysis (ABSA) provides this granular capability by identifying sentiment polarity toward particular entities and their attributes within a text (Ocampo Diaz et al., 2020). This task is a cornerstone of modern media monitoring, enabling organizations to track nuanced opinions across a wide range of topics, including products, policies, and public figures as they evolve in real-time.

While the term ABSA often refers to sentiment toward general attributes (e.g., product quality or price), our work focuses on sentiment directed at specific named entities, which, in our case, are company and brand names. This formulation aligns more precisely with what the literature defines as Entity-Level Sentiment Analysis (ELSA) (Rønningstad et al., 2022) or Targeted Sentiment Analysis (TSA) (Song et al., 2023). Throughout this paper, we use the term ABSA to remain consistent with our industrial application domain framing and the broader research category, where our task is specifically document-level, entity-targeted sentiment classification. Furthermore, for clarity and consistency, we will use the term “aspect” as a shorthand to refer to these target entities (in our case, company names). This distinction is critical in a media monitoring context, where the goal is to track the reputation of specific organizations rather than abstract features.

However, moving ABSA from controlled benchmarks to real-world, multilingual media monitoring presents significant challenges. A primary obstacle is the disparity in resources between languages. Although most state-of-the-art models and large-scale datasets are English-centric, this leaves morphologically complex, less-resourced language families such as South Slavic largely underdeveloped (Chebolu et al., 2023). Furthermore, the nature of news media introduces specific data complexities. Unlike typical review datasets, news articles are often long-form documents where sentiment toward an aspect can be subtly expressed across multiple paragraphs, a challenge that sentence-level ABSA methods struggle to address (Chebolu et al., 2023). The data is also frequently imbalanced, with neutral or majority-class sentiments dominating. Perhaps most critically for industrial applications, media monitoring systems must be able to dynamically handle unseen aspects, i.e., new companies, products, or topics that were not present in the training data, requiring models with strong generalization capabilities.

In this study, we introduce AspectBench and use it as an evaluation suite for document-level, entity-targeted sentiment classification. The task focuses on long news articles, where the same article may mention several organizations and where a target entity may be seen during training or appear only at test time, enabling us to explicitly evaluate generalizability. Using AspectBench, we evaluate a spectrum of modeling strategies that correspond to realistic deployment choices. The dataset comprises carefully cleaned and curated ABSA data sourced from real-world news articles written in Slovenian and Serbo-Croatian. The Serbo-Croatian portion of the dataset follows the ISO 639-3 “HBS” macrolanguage convention, which groups Bosnian, Croatian, Montenegrin, and Serbian under a single macrolanguage identifier while preserving their individual language codes (SIL International, 2026). This grouping is also common in South Slavic NLP work because these standards are highly mutually intelligible, frequently treated as a single pluricentric language in linguistics, share modeling resources, and are often grouped in NLP research due to their high linguistic overlap (Rupnik et al., 2023). We use the label Serbo-Croatian throughout the paper for this macrolanguage grouping, while noting that the dataset contains mostly Serbian and Croatian articles, a small number of automatically flagged Bosnian articles (136 in total), and both Latin and Cyrillic script.

Addressing these challenges requires a systematic evaluation of diverse modeling approaches to understand their respective trade-offs. Our goal here is to benchmark and evaluate existing approaches with necessary adaptations or optimizations and to assess them for industrial usage in multilingual settings. Using our AspectBench dataset, we conduct an empirical evaluation across the model families and deployment choices that are most relevant for this setting.

We evaluate five distinct approaches to modeling: (1) lightweight document embeddings test whether a single dense representation is sufficient for entity-targeted sentiment; (2) fine-tuned PLMs test the performance of end-to-end multilingual, short- and long-context, encoder-only, text-to-text, and language-specific supervised experts; (3) hierarchical architectures test whether explicit sentence-level aggregation helps on long documents at the cost of training complexity; (4) zero- and few-shot prompting of LLMs tests whether small open-weight quantised LLMs can solve the task without supervised expert models; and finally, (5) learning-to-defer (L2D) experiments help separate three questions, asking whether multiple expert models can be combined or whether they have similar failure points, whether an LLM can act as an adjudicator on every expert decision, and whether confidence-gated escalation on expert predictions can identify a smaller set of cases where LLM reasoning is actually useful.^1^

This design addresses a clear gap in the literature by providing a systematic comparative study of these distinct approaches on *long-form news* in South Slavic languages, with a specific focus on generalization to unseen aspects.^2^ Addressing all these challenges requires a benchmark and evaluation design that reflect the way entity sentiment is used in media monitoring.

In summary, our main contributions are as follows.

- AspectBench: We introduce a document-level, entity-targeted ABSA benchmark for South Slavic news, with Slovenian and Serbo-Croatian subsets, long media-monitoring documents, and a test design that explicitly measures generalization to unseen target entities. The Serbo-Croatian subset is made publicly available.
- Controlled Empirical Evaluation: We compare dense document embeddings, XLM-RoBERTa variants (Conneau et al., 2020), Longformer (Beltagy et al., 2020), mDeBERTa-v3 (He et al., 2023), mT5 (Xue et al., 2021), Slovenian and Serbo-Croatian language-specific encoders like SloBERTa (Ulčar and Robnik-Šikonja, 2021) and BERTić (Ljubešić and Lauc, 2021), and a streamlined hierarchical XLM-RoBERTa model (Yan et al., 2024) under consistent metrics and seen/unseen splits. This separates the effects of supervision, input length, language specialization, document aggregation, and aspect masking.
- Deferral Analysis for Expert and LLM Systems: We evaluate non-LLM expert policies, including voting, probability averaging, confidence selection, and shallow stackers (Wolpert, 1992; Breiman, 2001; Hastie et al., 2009); complete LLM deferral, where every expert prediction is adjudicated by a DSPy-optimized LLM prompt; and selective LLM deferral, where only low-confidence expert predictions are routed to the LLM. Rather than proposing a new L2D objective, we provide a systematic empirical evaluation of uncertainty-gated, DSPy-optimized complete and selective expert to LLM deferral for document-level entity-targeted ABSA in South Slavic news.
- Minimum Viable Set Analysis: We analyse how performance, class behavior, calibration, and model uncertainties change as the amount of labeled training data increases. These results provide a planning lens for future annotation campaigns by showing that the required dataset size depends on the target criterion, such as macro-F1, QWK, minority-class behavior, or uncertainty stabilization.
- Practical Findings for Media Monitoring: We show that strong supervised expert models remain the most reliable foundation, aspect masking is generally useful for expert-model variants but not universal, complete LLM deferral is unstable as a full-stream replacement, and selective LLM deferral can improve or stabilize performance when expert uncertainty identifies difficult target-attribution cases.

The remainder of this paper is structured as follows. Section 2 reviews related work. Section 3 describes the construction and characteristics of AspectBench. Section 4 outlines the evaluation protocol. Section 5 presents the research questions and methodological scope. Section 6 describes the evaluated models and deferral systems. Section 6 presents the empirical results and error analyses, which are then discussed in Section 7. Section 8 concludes the paper. Supplementary material is organized into dataset and preprocessing details (Supplementary Section S1), prompting details (Supplementary Section S2), complete and selective deferral diagnostics (Supplementary Section S3), qualitative and minimum viable set diagnostics (Supplementary Section S4), and model hyperparameters (Supplementary Section S5).

## 2 Related work

The progression from coarse-grained sentiment analysis to Aspect-Based Sentiment Analysis (ABSA) marks a critical shift toward the fine-grained understanding required for industrial applications such as media monitoring (Zorenböhmer et al., 2025). While early work established core subtasks such as Aspect Term Extraction (ATE) and Aspect Sentiment Classification (ASC) (Huang et al., 2025), applying these concepts to real-world data introduces significant challenges related to document length, language diversity, and model generalization. Our work builds upon several interconnected lines of research that address these complexities.

The terminology around ABSA, TSA, and ELSA is overlapping rather than strictly separated. Pei et al. (2019) describe TSA as also being known as ABSA and note that datasets vary in how they define core concepts such as “target” and “aspect.” In the more traditional ABSA formulation, aspects often refer to attributes or components of an entity, such as product quality or price. In TSA, the target is often a concrete entity, mention, or entity-aspect pair. ELSA makes the entity-level framing explicit by asking for sentiment toward volitional entities such as organizations or persons, especially in longer documents where sentence-level target sentiment may not aggregate cleanly to an entity-level judgment (Rønningstad et al., 2022). Our task sits at the intersection of these formulations. The industrial label is ABSA, the target units are named entities, and the prediction is a document-level sentiment label for each article-entity pair. We therefore use ABSA as the broad task label while defining the specific task as document-level, entity-targeted sentiment classification.

Several news-oriented datasets motivate this distinction. RuSentNE evaluates sentiment toward named entities in Russian news sentences (Golubev et al., 2023). NewsMTSC frames the news setting as multi-target-dependent sentiment classification, emphasizing that articles can contain multiple entities and indirect sentiment cues (Hamborg and Donnay, 2021). SToNe frames Croatian headline analysis as TSA while explicitly distinguishing entity-level sentiment toward a named entity from the overall tone of the headline (Barić et al., 2023). These examples show that news sentiment resources use ABSA-adjacent terminology differently depending on whether the focus is an entity, an entity-aspect relation, a document, a sentence, or a headline. AspectBench complements this work by focusing on South Slavic media monitoring, full-document inputs, and explicit seen/unseen target-entity splits.

The evolution of ABSA modeling approaches reflects a continuous effort to capture context better. While early neural networks improved upon feature-based methods (Ding et al., 2024), the advent of Pre-trained Language Models (PLMs), such as BERT and XLM-RoBERTa, established a new state-of-the-art by leveraging deep contextual understanding (Jayakody et al., 2024). However, the fixed input length of these standard PLMs poses a significant limitation for long news articles. To overcome this, Hierarchical Attention Networks (HANs) were developed to explicitly model inter-sentence dependencies, which proved to be effective for document-level tasks by processing the text in stages (Ruder et al., 2016). Our evaluation of standard PLMs against a streamlined hierarchical architecture directly investigates this trade-off between contextual scope and architectural complexity.

The recent emergence of Generative decoder-only Large Language Models (LLMs) has introduced a new paradigm centered on in-context learning and prompting. Although the few-shot capabilities of LLMs are well-documented (Wang et al., 2024), their application to ABSA is an active area of research. Techniques such as Chain-of-Thought (CoT) prompting, which elicits step-by-step reasoning, have shown promise for complex tasks (Wei et al., 2022), and prompt optimization methods are being developed to tune LLM performance for ABSA without full fine-tuning (Sun et al., 2024). Wu et al. (2025) conduct a multilingual evaluation of several LLMs on ABSA tasks, but their primary focus remains on high-resource languages (English, Spanish, French, Dutch, and Russian), leaving under-resourced language families like South Slavic unexamined. Building on this, an advanced line of inquiry explores how LLMs can be combined with external tools or specialized models (Paranjape et al., 2023). In this paper, we evaluate this interaction empirically, where the LLM is not treated as a replacement classifier by default, but as a possible adjudicator whose usefulness depends on the expert signal, the routing policy, and the cost of unnecessary interventions.

A significant challenge in deploying AI for high-stakes applications is ensuring trustworthiness and reliability. A well-established paradigm to address this is Human-in-the-Loop (HITL) decision-making, where predictions from a primary model that are deemed uncertain are flagged for review by a human expert (Thuy and Benoit, 2024). Recent work has begun to explore automating this process, replacing the human expert with a general-purpose LLM acting as a reasoning engine (Li et al., 2025). This approach is also closely related to automated methods of selective classification or reject-option classification, where a model trades coverage for lower risk by abstaining on difficult cases (Geifman and El-Yaniv, 2017). It is also formalized as learning to defer (L2D), where a classifier is trained with a rejector that decides whether another decision maker should handle the item (Madras et al., 2018; Mozannar and Sontag, 2020). Recent L2D work broadens this framing to expert-conditional advice and adaptive model selection (Montreuil et al., 2026; Kondadadi and Ortega, 2026), and prompt-optimization work shows that DSPy can be used to optimize structured LLM programs and demonstrations (Soylu et al., 2024). Our work does not claim a new formal L2D objective, nor does it claim novelty for uncertainty-based LLM deferral in general. Instead, we contribute a task-specific empirical deferral analysis for document-level entity-targeted ABSA whereby we compare non-LLM expert policies, complete LLM deferral, and selective LLM deferral under the same benchmark, and we test whether expert confidence can identify cases where a DSPy-optimized local LLM is more useful than harmful. To our knowledge, this is the first systematic evaluation of uncertainty-gated, DSPy-optimized selective LLM deferral for document-level entity-targeted ABSA in South Slavic news.

Concurrent with model development is the critical need for robust and representative benchmarks. Although foundational ABSA datasets like SemEval are English-centric and review-based, recent efforts such as M-ABSA (Wu et al., 2025) and UzABSA (Matlatipov et al., 2024) have expanded multilingual coverage. However, the majority of existing ABSA research continues to focus on product reviews or social media posts, while the news domain remains significantly underexplored, despite its practical importance in media monitoring and public discourse analysis. A recent systematic review found that over 70% of ABSA datasets are based on commercial reviews, leaving other critical domains like news media significantly underrepresented in benchmark creation (Hua et al., 2024).

Moreover, a gap persists for the South Slavic language family. Many existing benchmarks do not adequately test for out-of-distribution (OOD) generalization, a critical failure point for models that may overfit to spurious correlations in training data (Bae et al., 2025; Feger et al., 2025). To address this, modern benchmark design emphasizes the creation of challenging test sets that evaluate generalization to unseen entities or concepts (Heng et al., 2024; Xing et al., 2020). Our creation of AspectBench directly addresses these gaps by introducing a South Slavic news ABSA benchmark with label-partially-unseen splits, bridging both the domain and resource limitations in existing evaluations.

A further line of work concerns annotation planning under limited labeling budgets. Learning-curve methods estimate how classifier performance changes as additional labeled examples become available, and have been used to predict the sample size needed to reach a target performance level (Figueroa et al., 2012; Chang et al., 2023; Banko and Brill, 2001). In NLP, this question is also related to annotation-stopping work, where learning curves are used to monitor whether further annotation is likely to produce meaningful gains (Tomanek and Hahn, 2008), and to active learning, which reduces labeling effort by choosing informative examples rather than annotating a fixed random sample (Settles, 2010; Schröder and Niekler, 2020; Jacobs et al., 2021; Weeber et al., 2021). Our minimum viable set analysis differs from active acquisition methods in that it does not propose a sampling policy. Instead, it retrospectively measures how quickly useful task signals emerge under increasing labeled-data fractions based on our existing two datasets, and shows that the answer depends on the selected operating criterion, i.e., early learning-curve gains, near-final macro-F1, minority-class behavior, and uncertainty stability do not necessarily stabilize at the same point.

In summary, while prior work has advanced ABSA across model types and languages, gaps remain in benchmarking under-resourced contexts like South Slavic news and in evaluating when LLMs should interact with specialized expert models. Our work addresses these gaps by introducing a new benchmark and providing a comprehensive evaluation that compares expert models, expert-combination policies, complete LLM deferral, and selective LLM deferral under the same document-level entity-targeted ABSA setting.

## 3 The AspectBench dataset

This section details the construction, characteristics, and motivation behind AspectBench, our novel dataset for Aspect-Based Sentiment Analysis (ABSA) in South Slavic languages.

### 3.1 Motivation and industrial relevance

The proliferation of digital news and social media necessitates advanced tools for media monitoring, particularly for understanding nuanced public opinion toward specific entities or topics. ABSA addresses this by identifying the sentiment expressed toward particular aspects within a text. However, the development and evaluation of robust ABSA models are often hampered by the lack of suitable, large-scale, and domain-specific datasets, especially for less-resourced languages such as those in the South Slavic group.

AspectBench is developed to address this gap, focusing on the needs identified within the project. The dataset is derived from real-world news clippings, reflecting the type of content encountered by media monitoring agencies. We were motivated by the need to create a benchmark that supports the evaluation of models on their ability to generalize to unseen aspects and handle the linguistic variety and noise inherent in news articles from the Slovenian and Serbo-Croatian language areas. This aligns with the practical industry requirement of tracking sentiment toward emerging entities or topics not present in the training data, for instance, new clients like companies, institutions, or public figures in the media monitoring sector that were not part of the original annotation pool.

A second industrial motivation concerns annotation planning. Media-monitoring datasets are expensive to extend because each additional language, market, or client portfolio requires target-specific annotation by trained analysts. We therefore include a minimum viable set analysis to estimate how performance, calibration, and uncertainty change as progressively larger portions of the training data are used. The goal here is not to identify a universal minimum dataset size. Instead, the analysis provides a practical planning framework, since a campaign optimized for early macro-F1 gains may stop at a different point than one designed to stabilize minority-class behavior, QWK, or calibrated uncertainty estimates. This is useful for future extensions of AspectBench and for related industrial annotation campaigns where the final sentiment distribution is not known in advance.

### 3.2 Source and annotation process

The raw data for AspectBench consists of a large collection of analyzed, publicly available online news media and social media outlets covering diverse topics from Slovenia and the broader Southeastern European region. The aspect-based sentiment annotations were provided by Kliping d.o.o., a Ljubljana-based international media monitoring and analysis agency that operates across these markets and offers analytics and intelligence services for public sector and corporate clients.

Our corpus comprises client-ordered news clippings in Slovenian and the Western Balkans, reflecting real-world coverage tailored for monitoring emerging topics, entities, and sentiment trends. The annotation involved assigning associated sentiment polarity to document-aspect pairs. Importantly, the task was sentiment annotation for prespecified document and aspect pairs rather than open-ended aspect extraction, where the annotators judged the polarity expressed in the document with respect to a target entity, typically a company, organization, product, or other monitored entity. This multilingual annotation effort was conducted by trained native speakers using a shared annotation schema across all languages present in the initial dataset to ensure consistency across the languages. The annotators were media-monitoring analysts trained within Kliping's production annotation workflow. Before annotating independently, analysts were trained by a supervisor in the use of the annotation schema and the expected interpretation of sentiment labels.

Initially, the dataset comprised approximately 219k articles. Each article was annotated with one or more aspect-sentiment pairs. An “aspect” in this context refers to a specific entity, typically a company or product name, mentioned within the article. The associated “sentiment” indicates the polarity of the opinion expressed toward that aspect, categorized as positive (+1), neutral (0), or negative (–1). Following the annotation schema used in the media-monitoring workflow, positive sentiment denotes a document containing favorable context toward the target entity; neutral sentiment denotes a factual, mixed, unclear, or non-evaluative mention toward said entity; and negative sentiment denotes an unfavorable context, such as criticism, reputational harm, adverse events, financial or legal difficulties, or other negatively framed coverage in isolation or in comparison with any additional entities present in the document.

Each document-aspect pair was single-coded by one trained analyst. The production workflow did not include systematic double annotation of every item, and therefore standard inter-annotator agreement statistics such as Cohen's κ cannot be computed retrospectively for the released corpus. The quality of the data was instead controlled through a typical industrial annotation process, where supervisors reviewed portions of analysts' coded work for accuracy, the annotators received training before the independent coding phase, and annotation quality was also subject to feedback from end users of the media-monitoring service. We acknowledge that this differs from a controlled academic annotation campaign with double coding and adjudication, and we therefore treat the annotations as expert production labels rather than independently adjudicated gold-standard labels.

A significant portion of the articles (approximately 75.2k or 34.3%) contain annotations for multiple aspects, leading to a total of 295.2k individual aspect-sentiment instances after flattening the dataset (i.e., creating a separate data point for each aspect-sentiment pair within an article). Example records are provided in Supplementary Section S1.1, “Example Dataset Records”.

### 3.3 Pre-processing and normalization

The raw data underwent a multi-step preprocessing pipeline to ensure reliability for model training and evaluation; Supplementary Section S1.2, “Data Preprocessing”, describes the full pipeline and Supplementary Figure S1 shows the corresponding dataset-size changes. The key steps were (1) outlier removal based on article length, (2) URL normalization, (3) language identification and filtering using FastText (Joulin et al., 2016, 2017), (4) aspect keyword mention filtering to retain only articles explicitly mentioning the target aspect, and (5) final length normalization. This resulted in the final AspectBench splits, with 82.7k items in the Serbo-Croatian subset and 51.6k items in the Slovenian subset.

### 3.4 Dataset statistics and characteristics

The sentiment distribution shows a significant skew, particularly in the Slovenian subset (16.3% positive, 82.6% neutral, 1.2% negative), reflecting natural occurrences of sentiment in news media. The Serbo-Croatian subset exhibits a more balanced distribution (43.0% positive, 49.5% neutral, 7.5% negative). Full distribution visualizations are provided in Supplementary Section S1.3 and Supplementary Figure S2.

Aspect Distribution: The dataset contains a diverse range of aspects. The Serbo-Croatian subset features 65 unique aspects, while the Slovenian subset features 49 unique aspects. The distribution of instances per aspect varies, with some high-frequency entities and a long tail of less frequent ones.^3^ This diversity is essential for evaluating the generalizability of the model.

Multi-Aspect Articles: While flattening the dataset creates individual instances for each aspect-sentiment pair, it is important to note that the original articles often discussed multiple tracked entities.

### 3.5 Task definition

AspectBench is designed to evaluate the task of document-level, entity-targeted sentiment analysis. Given an entire news article and a specific target aspect (e.g., a company name) that is mentioned within that article, the goal is to classify the sentiment expressed *toward that specific aspect* within the context of the *entire document* as positive, neutral, or negative. The models are expected to perform this task for both the Serbo-Croatian and Slovenian languages. A key challenge, and a focus of our evaluation, is the ability of models to generalize to aspects not seen during training.

### 3.6 Dataset availability

While the dataset consists of two subsets, namely Serbo-Croatian and Slovenian, only the Serbo-Croatian subset is made available for academic research, and the Slovenian subset remains private due to privacy and commercial agreements with our industrial partner (see Data availability). This limited release is a constraint of the present benchmark. It means that the publicly available portion does not cover the full multilingual collection used in our internal experiments, and future work should therefore further validate the findings on additional similar publicly distributable datasets and annotation campaigns.

### 3.7 Limitations of the dataset

AspectBench should be interpreted as a realistic media-monitoring benchmark rather than as a uniformly sampled or fully adjudicated sentiment corpus. Its industrial origin provides naturally occurring, operationally relevant ABSA instances, but also introduces several constraints.

First, the distribution of aspects, entities, topics, and domains is shaped by client-ordered monitoring needs at the time of annotation and by entities that are salient in public news and social media coverage in the region. Therefore, the benchmark is not intended to be a uniformly sampled representation of all possible entities, domains, or topics in Slovenian and Western Balkan media. It may overrepresent commercially, institutionally, or publicly monitored entities, and should not be interpreted as an unbiased estimate of regional media sentiment in general.

Second, as described in Section 3.2, the annotations originate from a production media-monitoring workflow rather than a controlled academic annotation campaign. The document-aspect pairs were single-coded by trained analysts, with supervisor quality checks applied to portions of the coded work, but without a systematic double annotation and adjudication. Due to this, standard inter-annotator agreement statistics such as Cohen's κ cannot be computed retrospectively. The labels should therefore be treated as expert production annotations rather than independently adjudicated gold-standard labels.

Third, as described in Section 3.2, the public release is limited to the Serbo-Croatian subset, while the Slovenian subset remains private due to privacy and commercial restrictions associated with the industrial data provider. This limits full reproducibility for experiments involving the complete multilingual corpus and motivates future validation on additional publicly distributable datasets and annotation campaigns.

Overall, these limitations do not undermine the benchmark's intended use, but they do define its scope. AspectBench is designed to evaluate ABSA methods in a realistic regional media-monitoring scenario, not to provide a uniformly sampled, fully open, or exhaustively adjudicated sentiment corpus.

## 4 Evaluation protocol

A rigorous and consistent evaluation protocol is essential for a meaningful comparison of different ABSA approaches and for understanding their generalization capabilities. This section outlines our strategy for dataset splitting, the metrics used for assessment, and brief notes on implementation.

### 4.1 Train/test split strategy

To evaluate model performance, especially the ability to generalize to previously unseen aspects—a critical requirement in real-world media monitoring—we adopted a label-partially-unseen split strategy for both the Slovenian (SL) and Serbo-Croatian (SC) subsets of AspectBench. This approach, where a subset of aspect labels is deliberately withheld from the training and validation sets and appears only in the test set, aligns with recent recommendations for evaluating zero-shot and generalization capabilities in text classification (Shu et al., 2022; Yin et al., 2019).

For each language, the data was divided into a combined training-validation set and a holdout test set. To ensure reproducibility and mitigate sampling bias in the training-validation portion, we created three distinct train-validation splits using stratified sampling based on sentiment labels, employing different random seeds for each split. The models were trained and validated on these splits, and the final performance is reported as the average of the three runs on the single fixed holdout test set.

#### 4.1.1 Constructing the “aspect robustness test set”

The holdout test sets were carefully constructed to probe the generalization of the model to novel aspects. Our selection criteria for aspects to be included exclusively in the test set were:

1. High-Frequency Aspects: Inclusion of very common entities [e.g., “Audi,” “A1 Slovenija” for SL; “mts” (Telekom Srbija) for SC] that models would not have encountered during training. This simulates the challenge of handling prominent new market players or topics.
2. Low-Frequency Aspects: Inclusion of uncommon or rare entities (e.g., “Prva osebna zavarovalnica,” “Energetika Ljubljana” for SL; “Mobi Banka,” “API bank” for SC) to test performance on the long tail of aspect distribution.
3. Sentiment-Balanced Aspects: Where possible, inclusion of aspects with a relatively balanced distribution of negative, neutral, and positive sentiments (e.g., “Mlinotest” for SL; “Knez Petrol” for SC) to ensure models are tested on diverse sentiment contexts for unseen targets.

This mixed-criteria approach for selecting unseen aspects aligns with best practices for creating test sets that can reveal performance drops when models encounter new, underrepresented, or previously unseen targets, as advocated by Xing et al. (2020).

#### 4.1.2 Dataset split statistics

Articles ranged from approximately 250 to 5,000 characters. Under typical SentencePiece/BPE tokenization (Kudo and Richardson, 2018; Bommarito, 2025) schemes used by contemporary multilingual language models, this corresponds roughly to 60–1,500 tokens. Median article lengths were 2,300 characters (600 tokens) for Slovenian and 2,000 characters (500 tokens) for Serbo-Croatian.

Table 1 summarizes the sentiment distribution for the training-validation (Train-Val) and test sets for both languages. The overall sentiment distributions are preserved between the Train-Val and Test partitions through stratified sampling based on the original dataset proportions.

**Table 1:** Sentiment distribution in train-validation and test sets for Slovenian (SL) and Serbo-Croatian (SC) subsets of AspectBench.

Language	Split	Negative (–1)	Neutral (0)	Positive (+1)	Total
Slovenian	Train-Val	507 (1.2%)	36,230 (82.5%)	7,126 (16.3%)	43,863 (85%)
	Holdout test	90 (1.2%)	6,393 (82.6%)	1,257 (16.2%)	7,740 (15%)
Serbo-Croatian	Train-Val	5,255 (7.5%)	34,803 (49.5%)	30,249 (43%)	70,307 (85%)
	Holdout test	927 (7.5%)	6,142 (49.5%)	5,338 (43%)	12,407 (15%)

Counts (and percentages) are shown for negative (–1), neutral (0), and positive (+1) sentiment classes.

#### 4.1.3 Focus on unseen aspects in test sets

A key characteristic of our evaluation protocol is the deliberate inclusion of a substantial number of test instances associated with “test-only” aspects—aspect labels that do not appear in the training or validation sets. Table 2 summarizes the extent of this split in both languages.

**Table 2:** Contribution of test-only (unseen) aspects to the holdout test set, broken down by sentiment label.

Language	Unique Aspects	Negative (–1)	Neutral (0)	Positive (+1)	Total
Slovenian	$\frac{11}{47}$ (23.4%)	$\frac{78}{90}$ (86.7%)	$\frac{4,374}{6,393}$ (68.4%)	$\frac{908}{1,257}$ (72.2%)	$\frac{5,360}{7,740}$ (69.2%)
Serbo-Croatian	$\frac{10}{64}$ (15.6%)	$\frac{639}{927}$ (68.9%)	$\frac{2,896}{6,142}$ (47.2%)	$\frac{3,796}{5,338}$ (71.1%)	$\frac{7,331}{12,407}$ (59.1%)

The proportion of unique aspects that are unseen at training time (23.4% for SL, 15.6% for SC) aligns with zero-shot evaluation protocols proposed by Yin et al. (2019), where 15–30% of labels are held to meaningfully assess generalization without severely limiting the training signal. Here, unusually, these unseen aspects contribute a majority of the instances in the test set—69.2% for SL and 59.1% for SC. This design choice is intentional and better reflects likely real-world usage, as well as recent robustness benchmarks [e.g., ARTS (Xing et al., 2020), Likelihood Splits (Godbole and Jia, 2023)] that emphasize evaluating models under difficult, underrepresented, or entirely novel targets, where performance typically degrades most. We note that Slovenian and Serbo-Croatian differ slightly in their unseen label and token shares. This variation arises from their respective long-tail distributions, not from arbitrary thresholding. We deliberately maintain comparable criteria for selection (head, tail, balanced), prioritizing consistency in evaluation philosophy over numerical parity.

### 4.2 Metrics

Given the sentiment class imbalance observed in AspectBench (see Table 1), particularly for the negative class in Slovenian, we prioritize metrics that are resilient to such distributions. Our primary evaluation metric is Macro F1-score. We also report macro precision, macro recall, weighted F1-score (as a complementary F1 measure that accounts for class support), Quadratic Weighted Kappa (Graham and Jackson, 1993) which treats the sentiment classes as ordinal and penalizes misclassifications based on their distance from the true class, providing insight into the severity of errors, and per-class F1-scores (for negative, neutral, and positive classes) to understand model performance on individual sentiment categories.

Where applicable, we also analyse performance separately on instances corresponding to “seen” aspects (present in training) vs. “unseen” aspects (exclusive to the test set) to quantify generalization capabilities explicitly.

### 4.3 Implementation notes

All experiments were conducted using standard open-source libraries. Pre-trained language models were primarily sourced from the Hugging Face Transformers library (Wolf et al., 2020) while PyTorch (Paszke et al., 2017) served as the back-end for all our implementations. For few-shot LLM experiments, we used the DSPy framework (Khattab et al., 2023) for programmatic prompt optimization. Specific model architectures, base pre-trained checkpoints [e.g., “xlm-roberta-base” (Conneau et al., 2020)], and key hyperparameters for each experimented approach are detailed within their respective descriptions in Section 6 and summarized in Supplementary Section S5, “Model Hyperparameters and Training Details”.

Generative AI tools, specifically Gemini 2.5 Pro (Google DeepMind, 2025), Claude 4 and Claude 4.5 (Anthropic, 2025a,b), GPT-4o and GPT-5.5 (OpenAI, 2024, 2026), and Grok (xAI, 2025), were used to assist in the development workflow, including boilerplate code generation, bash scripting for experiment automation, and debugging. All AI-generated code was manually reviewed, tested, and validated by the authors to ensure correctness. Detailed scripts for reproducing these experiments are available (see Data availability).

## 5 Research questions and methodological scope

This study evaluates the effectiveness of several modeling approaches for Aspect-Based Sentiment Analysis (ABSA) on full-length news articles in South Slavic languages, in particular, the experimental groups reported in Tables 3–6. The following research questions guide our investigation:

- RQ1: Which expert-model families are most reliable for document-level entity-targeted ABSA in South Slavic news and with what general settings? We compare dense document embeddings, fine-tuned PLMs, language-specific encoders, and a hierarchical model to determine how much benefit comes from end-to-end supervision, longer input windows, language specialization, and explicit document context management.
- RQ2: When does aspect masking help? We evaluate masked and unmasked variants wherever the architecture supports a direct comparison. Masking is treated as an empirical input-conditioning strategy, not as a universally valid preprocessing rule.
- RQ3: Can learning-to-defer improve over single expert models? We first test non-LLM policies over the strongest PLM/HAN experts, including majority voting, probability averaging, confidence-based expert selection, and shallow stackers. These baselines ask whether expert diversity alone resolves difficult cases.
- RQ4: What role should LLMs play in this setting? We separate prompting-only LLMs, complete LLM deferral, and selective LLM deferral. Complete deferral tests whether an optimized local LLM can adjudicate the full stream of expert predictions; selective deferral tests whether expert uncertainty can identify a smaller set of cases where LLM reasoning is useful.
- RQ5: How much annotation is needed before future campaigns become useful? We use minimum viable set analysis to examine how macro-F1, QWK, per-class behavior, calibration, and fine-tuned model uncertainty change with increasing training-data size. This question is treated as an industrial planning analysis rather than as a universal sample-size guarantee.

**Table 3:** Experiments on the Slovenian dataset across all methods.

Strategy	Accuracy	Precision	Recall	F1	QWK	Negative F1	Neutral F1	Positive F1
Embeddings								
Baseline	90.17 (± 0.21)	79.00 (± 2.69)	63.16 (± 1.02)	68.39 (± 1.03)	0.640 (± 0.012)	40.98 (± 2.25)	94.18 (± 0.11)	70.02 (± 1.14)
Masked	90.39 (± 0.11)	75.34 (± 1.72)	62.86 (± 1.07)	67.21 (± 1.29)	0.653 (± 0.007)	35.82 (± 3.88)	94.29 (± 0.06)	71.53 (± 0.64)
Extractive	89.72 (± 0.07)	76.16 (± 1.91)	59.28 (± 1.50)	63.47 (± 0.89)	0.627 (± 0.012)	26.95 (± 1.95)	93.90 (± 0.09)	69.56 (± 1.24)
Fine-tuned PLMs								
XLM-R truncated	90.28 (± 0.33)	73.12 (± 1.55)	67.58 (± 0.29)	70.03 (± 0.80)	0.659 (± 0.013)	43.90 (± 1.67)	94.20 (± 0.19)	71.99 (± 1.13)
XLM-R extractive	90.35 (± 0.45)	74.25 (± 1.06)	69.51 (± 0.80)	71.42 (± 0.95)	0.679 (± 0.010)	46.20 (± 2.00)	94.16 (± 0.30)	73.90 (± 0.75)
XLM-R LLM summary	89.32 (± 0.64)	72.61 (± 4.35)	65.73 (± 1.56)	68.02 (± 0.48)	0.636 (± 0.011)	40.28 (± 0.40)	93.57 (± 0.43)	70.21 (± 0.87)
XLM-R masked LLM summary	89.12 (± 0.41)	71.77 (± 3.34)	67.65 (± 1.63)	69.41 (± 2.15)	0.630 (± 0.007)	45.42 (± 6.17)	93.45 (± 0.28)	69.35 (± 0.39)
Longformer baseline	90.28 (± 0.18)	73.72 (± 2.23)	69.58 (± 2.13)	71.47 (± 2.01)	0.663 (± 0.008)	48.12 (± 5.78)	94.19 (± 0.10)	72.09 (± 0.64)
Longformer masked	91.13 (± 0.43)	78.68 (± 2.77)	69.00 (± 0.55)	72.50 (± 1.05)	0.697 (± 0.013)	47.50 (± 1.89)	94.66 (± 0.26)	75.35 (± 1.10)
mDeBERTa-v3 baseline	89.83 (± 0.97)	74.59 (± 2.17)	68.30 (± 1.19)	70.71 (± 1.82)	0.660 (± 0.020)	46.18 (± 3.64)	93.84 (± 0.65)	72.11 (± 1.48)
mDeBERTa-v3 masked	90.62 (± 0.26)	77.41 (± 0.52)	67.75 (± 0.71)	71.30 (± 0.60)	0.678 (± 0.011)	45.86 (± 0.67)	94.36 (± 0.15)	73.66 (± 1.00)
mT5 baseline	90.25 (± 0.38)	71.45 (± 1.87)	65.21 (± 1.55)	67.75 (± 0.42)	0.661 (± 0.005)	36.53 (± 1.59)	94.16 (± 0.30)	72.57 (± 0.70)
mT5 masked	90.87 (± 0.24)	77.27 (± 1.44)	64.87 (± 1.91)	69.37 (± 1.12)	0.668 (± 0.013)	40.85 (± 3.08)	94.59 (± 0.14)	72.69 (± 1.30)
SloBERTa baseline	91.28 (± 0.25)	75.98 (± 1.33)	71.17 (± 2.52)	73.16 (± 1.03)	0.700 (± 0.006)	49.19 (± 3.57)	94.77 (± 0.16)	75.51 (± 0.75)
SloBERTa masked	91.62 (± 0.02)	77.19 (± 0.83)	70.03 (± 0.12)	73.03 (± 0.31)	0.710 (± 0.005)	47.59 (± 1.47)	94.98 (± 0.02)	76.52 (± 0.60)
Hierarchical								
HAN baseline	89.28 (± 0.25)	71.05 (± 0.71)	69.29 (± 0.69)	70.11 (± 0.43)	0.640 (± 0.007)	46.36 (± 1.92)	93.53 (± 0.15)	70.42 (± 0.72)
HAN masked	90.37 (± 0.15)	74.64 (± 1.30)	69.92 (± 1.39)	71.57 (± 0.47)	0.685 (± 0.001)	46.05 (± 1.58)	94.14 (± 0.11)	74.51 (± 0.10)
Prompting LLMs								
Direct prompting	61.06	43.72	70.22	42.52	0.310	11.22	72.74	43.61
DSPy direct prompting	61.18	42.40	68.96	42.55	0.280	14.88	72.48	40.27
DSPy COT	55.66	42.97	70.67	40.97	0.285	14.29	65.75	42.86
L2D using best PLMs								
Majority vote	92.73	87.88	70.55	76.35	0.742	54.55	95.66	78.86
Average probabilities	92.79	88.58	70.23	76.20	0.744	53.85	95.70	79.04
Longformer masked expert	92.25	86.93	70.44	75.88	0.729	54.55	95.36	77.72
Confidence pick	92.69	87.48	69.46	75.22	0.741	51.16	95.64	78.87
SloBERTa masked expert	92.40	80.78	70.46	74.53	0.733	49.66	95.46	78.46
Linear regression stacker	91.91	74.88	72.90	73.81	0.731	47.62	95.11	78.69
Random forest stacker	91.94	75.68	72.01	73.62	0.729	47.20	95.13	78.52
HAN-XLMR masked expert	91.46	80.13	67.56	72.16	0.697	46.38	94.91	75.20
Complete LLM deferral								
HAN softmax complete	90.05	72.09	71.70	71.77	0.678	47.34	93.94	74.04
HAN softmax complete + masked	90.13	71.95	71.81	71.77	0.678	47.06	94.00	74.26
HAN complete deferral	91.32	77.25	71.68	74.05	0.707	51.28	94.76	76.10
HAN complete deferral + masked	91.91	82.22	69.94	74.33	0.719	50.72	95.15	77.12
SloBERTa masked complete deferral	91.98	74.43	71.86	73.09	0.721	45.98	95.21	78.07
Longformer masked complete deferral	90.06	69.56	72.01	70.72	0.679	43.98	93.95	74.24
Selective LLM deferral								
Longformer selective deferral	92.62	86.89	72.29	**77.34**	**0.745**	57.35	95.57	79.09
Longformer selective deferral + masked	92.47	84.41	71.71	76.30	0.740	54.68	95.48	78.74
Automatic baselines/controls								
Document sentiment classifier	48.64	40.46	55.63	35.18	0.139	8.98	57.99	38.59
Majority class	82.60	27.53	33.33	30.16	*NA*	0.00	90.47	0.00

The top scores across each category (Accuracy, Precision, Recall, F1, QWK, Negative F1, Neutral F1, and Positive F1) for each Method have been underlined, while global high scores for each category have been marked in bold. Note that higher scores are better.

### 5.1 Embedding-based dense document representations

To explore if a single document-level embedding offers a sufficient representation for ABSA, we employ BGE-M3 (Chen et al., 2024), an embedding model supporting dense retrieval on documents up to 8,192 tokens, thereby allowing us to inherently process full document text without any transformation. BGE-M3 produces 1,024-dimensional embeddings. We then normalize these embeddings and use a multi-layer perceptron (MLP) comprising a 512-unit hidden layer (with ReLU and dropout), a 256-unit second hidden layer (again with ReLU and dropout), and a 3-unit output layer to classify these embeddings. We use 0.3 as the dropout rate. We train over 15 epochs with the AdamW optimiser (Kingma and Ba, 2015), learning rate 1e-4, batch size 64, and a weight decay of 0.01 with CrossEntropyLoss and a ReduceLROnPlateau scheduler, selecting the best checkpoint based on validation macro F1-score.

#### 5.1.1 Baseline BGE-M3 (full document embedding + MLP)

To establish a baseline for embedding methods, we encode the full, untruncated article by removing any pre-existing “<aspect>” and “</aspect>” tags from the raw article text. BGE-M3 directly encodes this into a single document embedding, which was subsequently fed into the standard [1,024 512 -> 256 -> 3] MLP.

#### 5.1.2 BGE-M3 with sentence filtering + MLP

To assess if focusing BGE-M3 on sentences containing aspect mentions improves performance, we extract only sentences containing mentions of the aspect forms/keywords. We then first concatenate the extracted sentences into a single block of text. BGE-M3 then encodes this condensed text to produce a single embedding similar to Section 5.1.1, which is subsequently classified by the MLP.

#### 5.1.3 BGE-M3 with aspect masking + MLP

To evaluate whether abstracting aspect mentions with a placeholder token helps with generalization, we replace all occurrences of “<aspect>... </aspect>”, i.e., the aspect-keywords, with a special “[ASPECT]” token. BGE-M3 then encodes this transformed text, and the embedding is classified using our standard MLP architecture.

### 5.2 Fine-tuning Pre-trained Language Models (PLMs)

We fine-tune several PLM families to separate conclusions about document-level entity-targeted ABSA from conclusions about any single encoder. XLM-RoBERTa remains the baseline multilingual encoder because it is a strong and widely used cross-lingual checkpoint for less-resourced languages (Conneau et al., 2020). Longformer is included as the long-context encoder baseline, since its sparse attention pattern can process substantially longer inputs than standard 512-token encoders (Beltagy et al., 2020). mDeBERTa-v3 provides a second multilingual encoder family based on disentangled attention and ELECTRA-style pre-training (He et al., 2023). mT5 provides a multilingual text-to-text alternative, allowing us to test whether casting sentiment classification as conditional label generation changes the performance profile (Xue et al., 2021). Finally, we include language-specific encoders, with SloBERTa for Slovenian and BERTić for the Serbo-Croatian subset (Ulčar and Robnik-Šikonja, 2021; Ljubešić and Lauc, 2021).

All added encoder baselines use the same article-aspect input structure. In unmasked runs, the original <aspect> and </aspect> tags are stripped from the article and the target entity string is passed as the paired input. In masked runs, the tagged target mention in the article and the paired aspect string are both replaced with “[ASPECT]”. This gives a direct masked/unmasked comparison for the encoder and text-to-text baselines while keeping the downstream task fixed as three-way sentiment classification.

#### 5.2.1 Base XLM-RoBERTa (truncated input)

To establish a strong baseline, we fine-tune XLM-RoBERTa with truncated inputs. For each instance, we first clean the article by removing the “<aspect>” and “</aspect>” tags. The input is a concatenation of the cleaned article with the aspect name as “[CLS] Cleaned-Article-Text [SEP] Aspect-Name [SEP]”. This combined sequence is truncated to a maximum of 512 tokens, with shorter sequences padded (46.85% for Slovenian, 53.68% for Serbo-Croatian fit within the context window).

#### 5.2.2 XLM-RoBERTa with extractive summarization (target filtering)

To mitigate information loss from simple truncation, this variant focuses the PLM on sentences that directly mention the aspect. We process each article using SpaCy (Montani et al., 2023)^4^ for sentence segmentation. We extract only sentences containing aspect keywords (marked by “<aspect>” tags), concatenate them, and clean the aspect tags from this condensed filtered text. This filtered text is then paired with the aspect string as “[CLS] Filtered-Article-Text [SEP] Aspect-Name [SEP]”. The input is again truncated to 512 tokens if necessary, though most extractive summaries (99.90% for Slovenian, 99.96% for Serbo-Croatian) fit within XLMR's context window.

#### 5.2.3 XLM-RoBERTa with LLM-assisted summarization

To provide XLM-RoBERTa with a dense, aspect-focused representation of the entire article, potentially capturing information that may be crucial beyond the truncation window, we used Gemma-3-27b-QAT (Gemma Team and Google DeepMind, 2025) to generate the summaries. To enhance summary quality and mitigate potential repetitiveness from the quantised model, we implement a 3-pass generation strategy with varied top-p, top-k, and temperature, selecting the most lexically diverse summary using the LexicalRichness library (Shen, 2022). These LLM-generated summaries, detailed in Supplementary Section S2.1, “LLM Summarization Prompt”, are also cleaned of residual aspect tags and formatted as “[CLS] LLM-Generated-Article-Summary [SEP] Aspect-Name [SEP]”. We also truncate the input to 512 tokens if needed, though most LLM summaries (99.77% for Slovenian, 99.98% for Serbo-Croatian) fit this limit.^5^ Since quantised LLMs tend to be somewhat error-prone, we also penalize token repetitions with a “repetition penalty” of 1.15 and additional logic checks to catch these errors so that we can requery with alternative sampling parameters.

#### 5.2.4 XLM-RoBERTa with LLM-assisted summarization and aspect masking

To understand whether learning a single masked representation offers any improvement over the non-masked version, we replicate Section 5.2.3 by replacing the aspect names with a “[ASPECT]” placeholder token (i.e., masking).

#### 5.2.5 Longformer

Longformer is used to test whether the main limitation of standard PLM fine-tuning is the 512-token input window. We fine-tune an XLM-RoBERTa-initialized Longformer checkpoint with a 4,096-token maximum sequence length and global attention on the first token. This keeps the article-aspect pair format used by the other encoder baselines while allowing substantially more of the news article to remain visible to the classifier.

#### 5.2.6 mDeBERTa-v3

mDeBERTa-v3 is included as a strong multilingual encoder from a different pre-training family than XLM-RoBERTa. We fine-tune the base multilingual checkpoint with the same article-aspect paired input format and a 512-token maximum length. This baseline helps determine whether the XLM-R results are specific to RoBERTa-style pre-training or persist under a DeBERTa-style encoder.

#### 5.2.7 mT5

mT5 is trained as a text-to-text classifier. The source sequence contains a short task prefix, the target aspect, and the article text; the target sequence is one of the three sentiment labels. At evaluation time, we avoid invalid free-form generations by scoring the three allowed label strings by conditional token loss and selecting the lowest-loss label. This keeps mT5 comparable to the classification models while respecting its encoder-decoder formulation.

#### 5.2.8 Language-specific encoders

We use SloBERTa for Slovenian and BERTić for the Serbo-Croatian subset to test whether language-specialized pre-training improves over broadly multilingual checkpoints. These models use the same paired article-aspect input format as the multilingual encoders, with language-specific tokenisers and 512-token maximum inputs.

### 5.3 Streamlined Hierarchical Attention Network (HAN) for document-level ABSA

To investigate the efficacy of hierarchical architectures for ABSA in long documents (Yang et al., 2016), we implemented a streamlined version inspired by architectures such as DART (Yan et al., 2024), which are designed to capture complex interactions within long texts. Our approach (which we call HAN or HAN-XLMR) uses XLM-RoBERTa as its core sentence encoder. The primary objective was to effectively process complete documents by first generating contextualized representations for individual sentences, which are augmented with aspect-specific information, and then employing a document-level attention mechanism to aggregate these sentence-level representations to classify the final sentiment.

A key motivation for our streamlined design was to enhance training stability and reduce computational overhead compared to the original DART architecture. The original DART (Yan et al., 2024) proposes a multi-stage interaction mechanism involving an interact-encoder operating on [CLS] token summaries of sentences and a refine-encoder that further processes the full token sequences of each sentence. Our HAN simplifies this by employing a single interaction transformer that operates exclusively on the [CLS] token embeddings derived from each sentence, augmented with learned sentence positional embeddings. This simplification removes the computationally intensive refine-encoder and the local token-level attention stage, aiming for a more tractable yet effective model, since we found that training the original implementation on our datasets required very careful hyper-parameter tuning, or else it ran into exploding/vanishing gradients.

The core HAN architecture consists of an XLM-RoBERTa-base sentence encoder, followed by a sentence interaction module and a global aggregation attention layer. For sentences, we set a maximum sequence length of 96 tokens. We processed up to 128 sentences per document (which were both above the max threshold of sentence length and max sentences for each article in both datasets). The sentence interaction transformer consists of two layers with eight attention heads each, and the global aggregation attention mechanism uses four heads. The final classification MLP had a hidden dimension of 256 units, and a dropout rate of 0.2 was applied throughout the network where applicable. We train for 10 epochs using the AdamW optimiser (Kingma and Ba, 2015) with a learning rate of 1e-5 and a weight decay of 0.01. We employed a micro-batch size of 4, with gradient accumulation to achieve an effective batch size of 32. Additionally, we use “CrossEntropyLoss” and a “ReduceLROnPlateau” learning rate scheduler, with model selection based on the best validation macro F1-score.

We conducted experiments to investigate the impact of hierarchical aggregation of attention compared to the XLMR baseline Section 5.2.1 and assess the usefulness of aspect masking. The following configurations were evaluated:

#### 5.3.1 HAN: baseline

In this configuration, we remove <aspect> and </aspect> tags, and the text is segmented into sentences. Each sentence is prefixed with the aspect name itself to prime the sentence encoder. The global aggregation query is the embedding of this same aspect name token, which the model learns to associate with aspect-relevant features from the sentence representations.

#### 5.3.2 HAN with aspect masking

To assess whether learning a unified representation for the aspect is beneficial, this setup replaces all occurrences of “<aspect>... </aspect>” in the original article with a “[ASPECT]” placeholder token. Each sentence is then prefixed with this same “[ASPECT]” placeholder before encoding, providing the model with two forms of aspect signaling, namely a general marker for each sentence and a specific placeholder at the aspect's original location.

### 5.4 Large Language Models (LLMs)

We also investigate the utility of open-source multilingual Large Language Models (LLMs) for ABSA on AspectBench, focusing on the Gemma-3-27b-qat model (Gemma Team and Google DeepMind, 2025). We additionally tested GaMS 27B and Qwen2.5 32B in zero- and few-shot settings; however, Gemma-3 27B consistently outperformed GaMS and Qwen by 20-30%. Given the breadth of our experimentation, we therefore continue with Gemma-3 27B QAT as our LLM base for all LLM-based experiments.

Our exploration spans from direct prompting to more sophisticated strategies involving prompt optimization and guided reasoning, primarily facilitated by the Declarative Self-improving Python (DSPy) framework (Khattab et al., 2023). DSPy allows developers to programmatically optimize prompts for LLMs by defining how the data flows and how small changes transform the outputs through composable modules and signatures. Signatures in DSPy declaratively specify the input and output fields for an LLM task (e.g., article and aspects as inputs, and a sentiment as output).^6^ These signatures can be combined into modules (like “Predict” for direct prediction or “ChainOfThought” for reasoning steps) to build complex programs. DSPy can then optimize these programs, including the underlying prompts and few-shot examples, using a separate LLM (a “teacher” model) and a small amount of training data.

Given that instruction-tuned LLMs can be highly sensitive to class imbalances in few-shot examples (Zhao et al., 2021), and to make the extensive optimization process computationally tractable, we curated a smaller, balanced training set for all DSPy optimizations. This set contains an equal number of negative, neutral, and positive examples sampled from the original train/validation splits.^7^ However, all final evaluations are performed on the original imbalanced holdout test set to ensure a realistic assessment of performance.

For all LLM experiments, we query the model 3 times per item and take the mode of the predictions, defaulting to “neutral” in case of ties or no valid responses.^8^ The LLM sampling parameters were generally set to a temperature of 1.0, top_k of 64, and top_p of 0.95. For DSPy-based methods, the student LLM (Gemma-3-27b-qat) was configured with a maximum token generation limit of 1024. We additionally employed Qwen-2.5-72b (Qwen Team, Alibaba Group, 2024a,b) as the teacher model for DSPy's MIPROv2 optimization process since we observed that it consistently produced better results than using Gemma-3-27b-qat to optimize itself. The MIPROv2 optimiser (Opsahl-Ong et al., 2024) was set to “heavy” for Slovenian and “medium” for Serbo-Croatian (as “heavy” tended to overfit on Serbo-Croatian, producing unreadable tokens). For the DSPy optimization itself, the MIPROv2 optimiser was also set with a temperature of 1.0 to encourage diverse exploration of prompt strategies. The optimization was performed using a class-balanced subset of the training and validation set, and the final scores are reported on the holdout test set.^9^ Additionally, all optimizations initialize with our handcrafted prompt (Section 5.4.1).

#### 5.4.1 LLM with direct prompting

We queried the LLM using manually crafted prompts to establish a zero-shot baseline. This prompt, detailed in Supplementary Section S2.2.1, “Direct Prompting”, instructs the LLM to analyse the sentiment toward a specified aspect within an article and respond with a single sentiment label (“negative,” “neutral,” or “positive”). The same prompt structure was used for both the Slovenian and Serbo-Croatian datasets.

#### 5.4.2 LLM with DSPy-optimized prompting

We use DSPy to optimize the prompting strategy for direct sentiment classification with a basic “SentimentClassificationSignature” (input: article, aspect; output: sentiment). The optimized prompts are available in Supplementary Section S2.2.1, “Direct Prompting.”

#### 5.4.3 LLM with DSPy-optimized Chain-of-Thought (CoT)

We use DSPy to optimize a Chain-of-Thought (CoT) prompting approach using the “ReasoningSentimentSignature” (input: article, aspect; output: reasoning, sentiment) to encourage more explicit reasoning. This allows for an inspection of the model's rationale. The optimized CoT prompts are included in Supplementary Section S2.2.2, “Chain-of-Thought Prompting”.

### 5.5 Learning to defer using best PLMs

Before introducing LLM-based deferral, we test whether the strongest supervised experts can improve by deferring among themselves. For each language, we select high-performing but architecturally diverse experts from the PLM/HAN results. Slovenian uses Longformer, SloBERTa, and HAN-XLMR. Serbo-Croatian uses Longformer, mDeBERTa-v3, BERTić, and HAN-XLMR; the HAN-XLMR expert is retained because it is the expert we used to compare how complete LLM deferral performed when using softmax vs. uncertainty scores.^10^

We evaluate four non-LLM policies over these experts. Majority voting chooses the sentiment label predicted by the largest number of experts. Probability averaging averages expert class probabilities before taking the final label. Confidence pick selects the prediction from the expert with the highest top-class confidence for the given instance. Finally, logistic-regression and random-forest stackers learn shallow combination rules over expert probabilities and confidence features (Wolpert, 1992; Breiman, 2001; Hastie et al., 2009). These policies test whether expert diversity alone resolves hard cases, and they provide a deployment-relevant baseline for the LLM deferral systems. We did not jointly train a full learned deferral model over all experts and an LLM; this remains a natural future-work direction once per-item expert predictions and routing costs are standardized.

### 5.6 Complete and selective LLM deferral

The LLM-assisted experiments are treated as learning-to-defer systems rather than as a single joint architecture. In complete LLM deferral, every test instance is passed to the LLM together with the article, the target aspect, the task description, and the prediction made by an expert model. The LLM is asked to keep the expert label, revise it, or abstain according to the optimized prompt program. This setting tests whether a local LLM can act as a universal adjudicator over the full evaluation stream.

We evaluate two strategies for complete deferral. The first is softmax-based deferral, where the LLM receives the expert's raw output probabilities (top-class probability, softmax probabilities per class, and validation-based reliability scores) for the article-aspect instance, framed as evidence the LLM should consider when reasoning toward a label. The second is uncertainty-based deferral, where the LLM receives richer uncertainty signals derived from the HAN [predictive entropy, top-class probability, and the calibration score from Monte-Carlo-dropout agreement (Hasan et al., 2022)]. These signals are incorporated through DSPy prompt optimization and are intended to help the LLM treat the case as potentially ambiguous before deciding whether to keep or revise the HAN prediction. For both strategies, we also evaluate a *masked* variant in which explicit aspect names are replaced by a placeholder token to test whether the LLM relies on lexical aspect cues vs. contextual evidence. As in the prompting experiments, we optimize a prompt for Gemma-3 27B QAT as the student model using Qwen 2.5 72B Q4_K_M as the teacher. We use MIPROv2 with medium (300 train-validation samples) and heavy (1,000 train-validation samples) settings to estimate the best prompt variant.

The softmax-only complete-deferral runs are weaker and less stable than the uncertainty-based runs. We therefore do not expand softmax-based complete deferral to every top PLM expert. The expanded complete-deferral rows focus on the stronger uncertainty-based design for the selected top experts.

In selective LLM deferral, the expert model first predicts every instance and ranks instances by confidence. Only the lowest-confidence fraction is routed to the LLM, and high-confidence instances retain the expert prediction. This turns the LLM into a targeted escalation mechanism rather than a replacement classifier. We evaluate fixed gate rates and report the final task metrics together with the LLM call rate. Selective deferral is evaluated for Slovenian with the Longformer expert since it had the best multi-expert policy by macro-F1, and for Serbo-Croatian with both Longformer and mDeBERTa-v3 experts.^11^ For each case, we preserve masked and unmasked prompt variants so that the masking analysis remains visible across expert-only, complete-deferral, and selective-deferral settings. Figure 1 provides an overview of the complete and selective LLM deferral pipelines.

**Figure 1:**
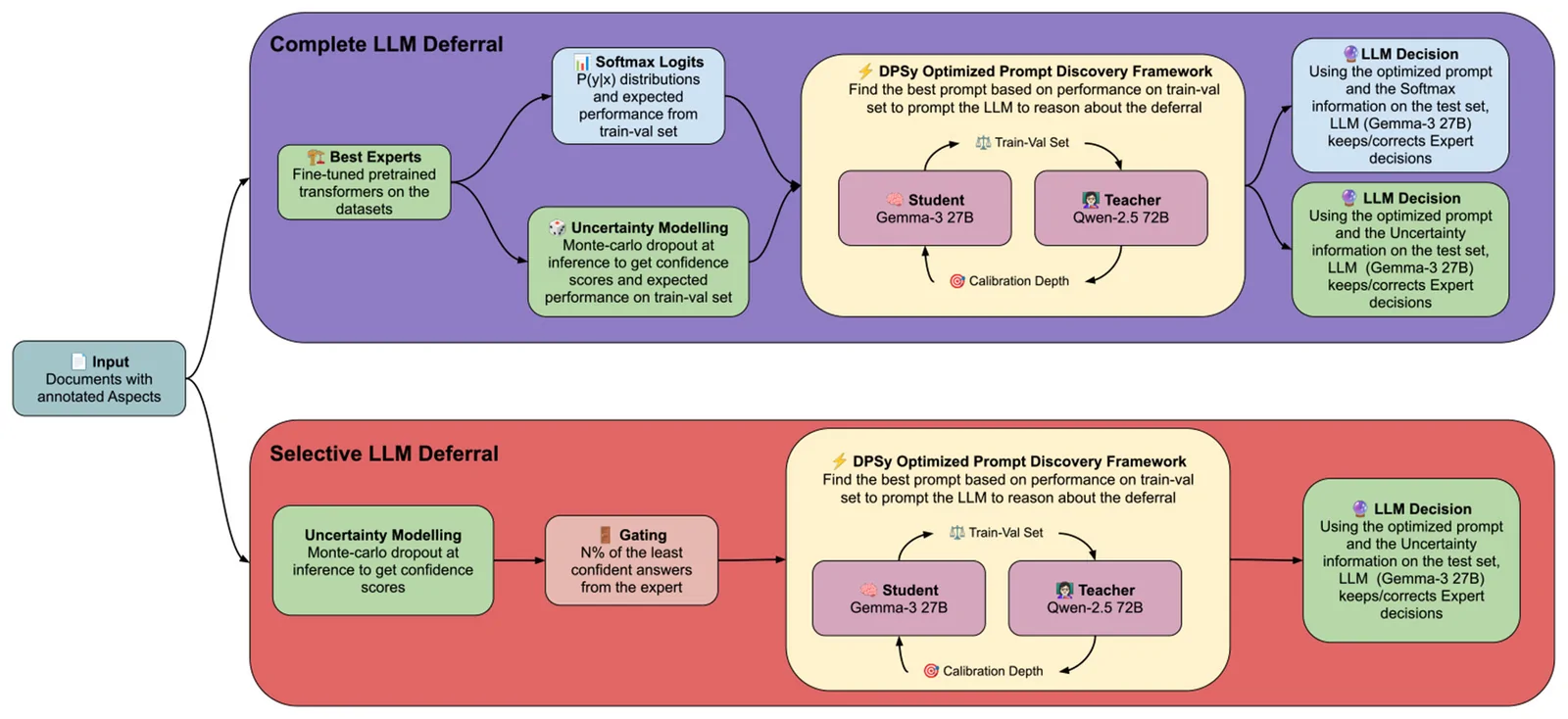
Overview of the LLM deferral experiments. Complete LLM deferral sends every article-aspect instance to the LLM together with the expert prediction and confidence information. Selective LLM deferral first uses the expert model's confidence to identify difficult instances and sends only this gated subset to the LLM, while high-confidence instances retain the expert prediction.

### 5.7 Minimum viable set analysis

We additionally run a minimum viable set (MVS) analysis to estimate how much labeled data is needed before performance, calibration, and uncertainty begin to stabilize. This analysis is not treated as another model family in Tables 3–6; instead, it is an annotation-planning diagnostic for the same document-level entity-targeted ABSA setting.

The experiment trains masked language-specific encoder experts on progressively larger stratified subsets of the training and validation data, using 1%, 2%, 5%, 10%, 20%, 30%, 40%, 50%, 75%, and 100%. Each percentage is evaluated with three run indices and validation-based early stopping. The held-out test set remains fixed, so changes in macro-F1, QWK, per-class F1, calibration, and uncertainty can be attributed to the available training volume rather than to a changing evaluation split. After training, we run Monte-Carlo dropout with ten stochastic samples per checkpoint to estimate predictive entropy, mutual information, confidence, Expected Calibration Error (ECE), and Brier score.

We summarize the MVS behavior with several complementary criteria, including relative macro-F1 drops from the full-data model (within 2%, 5%, and 10%), geometric knee detection on the scaling curve, a one-standard-error rule using fold variance, and an epistemic-uncertainty plateau based on mutual information. Since these criteria answer different practical questions, we do not collapse them into a single universal dataset-size recommendation.

### 5.8 Automatic baselines

As a control baseline, we use an off-the-shelf zero-shot cross-lingual news sentiment classifier from Andrenšek et al. (2024) in a zero-shot setting. We evaluate its performance on our two datasets to serve as a strong sentiment-only baseline. This helps contextualize the added value of aspect-level reasoning in our main evaluations.

As a trivial control baseline, we also report a majority class classifier, which always predicts the most frequent sentiment class in the test set. This baseline is widely used in sentiment analysis and text classification research as a lower bound for task difficulty (Wang and Manning, 2012; Zhang et al., 2015; Medhat et al., 2014). For our setting, the majority class is determined from the gold labels in the test data, and evaluation metrics are computed consistently with our main experiments, including macro-averaged F1, weighted F1, and quadratic weighted kappa (QWK). Since the classifier produces only a single label prediction, QWK values are undefined and reported as “N/A.” We further report results separately for seen and unseen aspects by stratifying the test sets according to whether aspect entities appeared in the training data, ensuring that this baseline is directly comparable to our model evaluations.

Additionally, we explored automated machine learning (AutoML) frameworks by applying AutoGluon (Tang et al., 2024) to fine-tune a multilingual ModernBERT model (Warner et al., 2025). However, this approach performed poorly, achieving a macro F1-score of approximately 15% on both language splits, indicating that standard AutoML pipelines may struggle with the document-level context and aspect-specific focus of this task without significant feature engineering or architectural customization.

## 6 Results and analysis

This section presents the empirical results of our experiments on the AspectBench dataset. We first provide an overall comparison of the best-performing model from each approach to establish a high-level view. We then delve into detailed analyses structured around our research questions, examining the performance of each model family, the impact of specific design choices like aspect masking, and performance differences across seen and unseen aspects. All reported scores are the average of three runs with different random seeds, with standard deviations provided where applicable.^12^ Detailed results, including per-class F1-scores, are available in Tables 3, 5, while performance on seen vs. unseen aspects is detailed in Tables 4, 6.

**Table 4:** Comparison of all methods across seen and unseen aspects for the Slovenian dataset.

Method	Learning strategy	F1 (seen)	F1 (unseen)	QWK (seen)	QWK (unseen)
Embeddings	Baseline	60.39 (± 0.77)	69.08 (± 1.04)	0.677 (± 0.020)	0.626 (± 0.010)
	Masked	62.21 (± 1.10)	67.46 (± 1.40)	0.699 (± 0.013)	0.635 (± 0.005)
	Extractive	63.45 (± 1.82)	62.90 (± 1.08)	0.689 (± 0.007)	0.602 (± 0.016)
Fine-tuned PLMs	XLM-R truncated	71.11 (± 2.11)	69.00 (± 1.26)	0.746 (± 0.006)	0.624 (± 0.018)
	XLM-R extractive	69.06 (± 0.93)	71.16 (± 1.14)	0.770 (± 0.005)	0.643 (± 0.012)
	XLM-R LLM summary	73.64 (± 3.27)	66.36 (± 0.55)	0.716 (± 0.026)	0.605 (± 0.006)
	XLM-R masked LLM summary	73.89 (± 2.04)	67.88 (± 2.63)	0.716 (± 0.008)	0.596 (± 0.013)
	Longformer baseline	71.77 (± 4.46)	70.40 (± 2.01)	0.766 (± 0.018)	0.622 (± 0.016)
	Longformer masked	73.03 (± 1.88)	71.64 (± 1.55)	0.786 (± 0.010)	0.663 (± 0.016)
	mDeBERTa-v3 baseline	68.84 (± 1.30)	70.16 (± 1.93)	0.738 (± 0.032)	0.629 (± 0.017)
	mDeBERTa-v3 masked	70.13 (± 0.22)	70.67 (± 0.78)	0.757 (± 0.005)	0.647 (± 0.016)
	mT5 baseline	65.46 (± 3.47)	67.37 (± 0.93)	0.731 (± 0.013)	0.634 (± 0.003)
	mT5 masked	62.79 (± 3.02)	70.07 (± 1.16)	0.713 (± 0.012)	0.651 (± 0.013)
	SloBERTa baseline	73.46 (± 0.92)	72.30 (± 0.96)	0.791 (± 0.011)	0.664 (± 0.011)
	SloBERTa masked	74.33 (± 1.66)	72.11 (± 0.45)	0.792 (± 0.015)	0.678 (± 0.008)
Hierarchical	HAN baseline	72.18 (± 1.92)	68.81 (± 0.58)	0.733 (± 0.003)	0.603 (± 0.010)
	HAN masked	72.20 (± 3.57)	70.95 (± 0.17)	0.746 (± 0.012)	0.661 (± 0.006)
Prompting LLMs	Direct prompting	38.85	44.37	0.283	0.323
	DSPy direct prompting	38.53	44.74	0.261	0.289
	DSPy COT	36.21	43.48	0.251	0.301
L2D using best PLMs	Majority vote	71.89	**76.40**	0.813	0.715
	Average probabilities	73.64	75.77	**0.826**	0.713
	Longformer masked expert	71.74	75.79	0.809	0.699
	Confidence pick	73.39	74.74	0.819	0.711
	SloBERTa masked expert	74.45	73.91	0.804	0.706
	HAN-XLMR masked expert	72.01	71.71	0.754	0.676
Complete LLM deferral	HAN softmax complete	70.85	71.38	0.744	0.652
	HAN softmax complete + masked	73.88	70.75	0.754	0.649
	HAN complete deferral	75.42	73.38	0.758	0.687
	HAN complete deferral + masked	77.29	73.27	0.785	0.694
	SloBERTa masked complete deferral	70.31	73.03	0.788	0.696
	Longformer masked complete deferral	65.18	72.52	0.689	0.675
Selective LLM deferral	Longformer selective deferral	**83.15**	76.17	**0.826**	**0.718**
	Longformer selective deferral + masked	75.95	75.96	**0.826**	0.714
Automatic baselines/controls	Document sentiment classifier	37.42	34.09	0.198	0.114
	Majority class	30.60	29.96	*NA*	*NA*

The top scores across each category (F1 and QWK, Seen vs. Unseen) for each Method have been underlined, while global high scores have been marked in bold. Selective LLM deferral rows use the best saved fixed gate for each prompt variant. Linear-regression and random-forest stacker rows are omitted from this seen/unseen breakdown because only aggregate policy metrics, not per-item predictions, were saved.

**Table 5:** Experiments on the Serbo-Croatian dataset across all methods.

Strategy	Accuracy	Precision	Recall	F1	QWK	Negative F1	Neutral F1	Positive F1
Embeddings								
Baseline	83.95 (± 0.12)	80.98 (± 0.17)	77.16 (± 0.27)	78.76 (± 0.25)	0.759 (± 0.002)	65.63 (± 0.60)	84.16 (± 0.20)	86.49 (± 0.01)
Masked	84.05 (± 0.22)	80.59 (± 0.12)	77.72 (± 0.18)	78.93 (± 0.11)	0.761 (± 0.002)	65.91 (± 0.33)	84.18 (± 0.37)	86.70 (± 0.22)
Extractive	81.60 (± 0.43)	78.39 (± 0.83)	72.70 (± 0.63)	74.88 (± 0.48)	0.720 (± 0.003)	58.12 (± 0.79)	82.06 (± 0.51)	84.45 (± 0.37)
Fine-tuned PLMs								
XLM-R truncated	85.42 (± 0.22)	84.63 (± 0.83)	78.32 (± 0.60)	80.84 (± 0.24)	0.789 (± 0.003)	69.36 (± 1.30)	85.76 (± 0.20)	87.41 (± 0.47)
XLM-R extractive	83.71 (± 0.13)	79.62 (± 0.51)	75.24 (± 0.39)	77.05 (± 0.39)	0.758 (± 0.005)	60.24 (± 1.07)	84.08 (± 0.17)	86.83 (± 0.13)
XLM-R LLM summary	84.66 (± 0.54)	82.07 (± 0.13)	77.69 (± 1.44)	79.52 (± 0.91)	0.783 (± 0.009)	66.45 (± 1.79)	84.79 (± 0.45)	87.30 (± 0.56)
XLM-R masked LLM summary	84.94 (± 0.33)	81.06 (± 0.83)	79.85 (± 0.45)	80.28 (± 0.43)	0.789 (± 0.006)	68.29 (± 0.82)	84.53 (± 0.48)	88.01 (± 0.13)
Longformer baseline	84.45 (± 0.34)	83.28 (± 0.60)	77.22 (± 0.50)	79.65 (± 0.14)	0.770 (± 0.005)	67.63 (± 0.69)	84.86 (± 0.22)	86.47 (± 0.64)
Longformer masked	85.87 (± 0.09)	81.39 (± 0.61)	81.43 (± 0.19)	81.37 (± 0.31)	0.800 (± 0.001)	69.60 (± 1.00)	85.80 (± 0.22)	88.69 (± 0.20)
mDeBERTa-v3 baseline	82.93 (± 1.78)	82.75 (± 0.63)	75.38 (± 2.28)	78.08 (± 1.69)	0.749 (± 0.028)	66.12 (± 1.81)	83.44 (± 1.01)	84.67 (± 2.83)
mDeBERTa-v3 masked	85.17 (± 0.40)	83.00 (± 0.73)	79.91 (± 0.02)	81.15 (± 0.34)	0.780 (± 0.004)	70.96 (± 0.32)	85.08 (± 0.64)	87.42 (± 0.24)
mT5 baseline	83.98 (± 0.21)	80.86 (± 0.99)	76.90 (± 1.48)	78.37 (± 0.53)	0.761 (± 0.007)	64.25 (± 1.22)	83.92 (± 0.19)	86.94 (± 0.46)
mT5 masked	83.89 (± 0.64)	78.48 (± 0.58)	80.15 (± 0.79)	79.07 (± 0.84)	0.766 (± 0.010)	66.43 (± 1.38)	83.59 (± 1.03)	87.19 (± 0.27)
BERTić baseline	86.31 (± 0.58)	84.49 (± 0.17)	79.74 (± 1.31)	81.70 (± 0.86)	0.802 (± 0.010)	70.03 (± 1.57)	86.38 (± 0.44)	88.68 (± 0.66)
BERTić masked	85.78 (± 0.38)	82.69 (± 0.61)	80.48 (± 0.36)	81.44 (± 0.17)	0.795 (± 0.004)	70.30 (± 1.21)	85.77 (± 0.37)	88.24 (± 0.57)
Hierarchical								
HAN baseline	83.86 (± 0.43)	80.81 (± 1.22)	78.57 (± 0.56)	79.50 (± 0.81)	0.764 (± 0.005)	68.34 (± 1.99)	83.74 (± 0.39)	86.41 (± 0.29)
HAN masked	84.62 (± 0.30)	80.39 (± 0.73)	80.84 (± 0.47)	80.53 (± 0.50)	0.784 (± 0.004)	69.84 (± 1.06)	84.36 (± 0.35)	87.39 (± 0.16)
Prompting LLMs								
Direct prompting	59.66	58.48	68.58	56.69	0.552	44.38	60.81	64.88
DSPy direct prompting	64.02	58.22	68.06	59.73	0.584	44.85	62.06	72.29
DSPy COT	66.37	60.35	69.23	61.98	0.598	47.32	65.77	72.83
L2D using best PLMs								
Confidence pick	87.79	85.18	83.32	**84.18**	0.829	74.84	87.75	89.94
Average probabilities	87.77	85.49	82.92	84.08	0.826	74.60	87.80	89.85
Majority vote	87.57	85.09	82.97	83.93	0.824	74.56	87.54	89.70
mDeBERTa-v3 masked expert	86.90	84.80	82.51	83.55	0.813	74.93	86.91	88.82
Random forest stacker	87.26	83.01	83.53	83.24	0.824	72.69	87.11	89.92
Longformer masked expert	87.02	82.73	83.04	82.86	0.820	71.97	86.92	89.70
BERTić masked expert	86.82	83.77	81.68	82.63	0.811	71.84	86.85	89.19
Linear regression stacker	86.86	81.82	83.48	82.59	0.818	71.26	86.74	89.77
Complete LLM deferral								
HAN softmax complete	84.65	80.14	81.53	80.78	0.784	70.56	84.53	87.24
HAN softmax complete + masked	84.79	80.09	81.69	80.83	0.786	70.33	84.70	87.44
HAN complete deferral	86.01	82.72	81.51	82.09	0.807	71.90	86.04	88.33
HAN complete deferral + masked	84.23	76.90	81.84	78.87	0.787	64.08	83.94	88.58
mDeBERTa-v3 masked complete deferral	85.20	80.35	82.06	81.05	0.793	70.12	84.80	88.23
Longformer masked complete deferral	85.08	77.94	82.80	79.90	0.798	65.64	84.77	89.27
BERTić masked complete deferral	83.79	77.50	80.85	78.81	0.778	65.49	83.09	87.83
Selective LLM Deferral								
Longformer selective deferral	87.63	83.95	84.26	84.07	**0.830**	74.73	87.41	90.06
Longformer selective deferral + masked	87.31	82.89	84.17	83.46	0.825	73.31	87.09	89.99
mDeBERTa-v3 selective deferral	87.26	83.51	84.13	83.74	0.827	74.53	86.87	89.83
mDeBERTa-v3 selective deferral + masked	87.18	83.23	83.60	83.36	0.818	73.36	87.05	89.66
Automatic baselines/controls								
Document sentiment classifier	67.55	57.29	59.80	58.14	0.439	31.76	66.72	75.94
Majority class	49.50	16.50	33.33	22.07	*NA*	0.00	66.22	0.00

The top scores across each category (Accuracy, Precision, Recall, F1, QWK, Negative F1, Neutral F1, and Positive F1) for each Method have been underlined, while global high scores for each category have been marked in bold. Note that higher scores are better.

**Table 6:** Comparison of all methods across seen and unseen aspects for the Serbo-Croatian dataset.

Method	Learning strategy	F1 (seen)	F1 (unseen)	QWK (seen)	QWK (unseen)
Embeddings	Baseline	87.76 (± 0.25)	72.28 (± 0.34)	0.839 (± 0.005)	0.706 (± 0.002)
	Masked	87.79 (± 0.24)	72.60 (± 0.14)	0.843 (± 0.005)	0.709 (± 0.002)
	Extractive	86.22 (± 0.27)	66.87 (± 0.90)	0.824 (± 0.005)	0.652 (± 0.006)
Fine-tuned PLMs	XLM-R truncated	91.50 (± 0.17)	73.53 (± 0.45)	0.891 (± 0.002)	0.725 (± 0.007)
	XLM-R extractive	89.68 (± 0.39)	68.30 (± 0.52)	0.865 (± 0.006)	0.690 (± 0.005)
	XLM-R LLM summary	88.74 (± 0.44)	72.98 (± 1.67)	0.860 (± 0.004)	0.733 (± 0.017)
	XLM-R masked LLM summary	88.39 (± 0.42)	74.45 (± 0.50)	0.852 (± 0.007)	0.748 (± 0.006)
	Longformer baseline	90.43 (± 0.85)	72.24 (± 0.71)	0.875 (± 0.008)	0.704 (± 0.012)
	Longformer masked	91.02 (± 0.51)	74.90 (± 0.71)	0.887 (± 0.005)	0.749 (± 0.001)
	mDeBERTa-v3 baseline	89.48 (± 0.99)	70.05 (± 3.23)	0.863 (± 0.017)	0.675 (± 0.051)
	mDeBERTa-v3 masked	90.07 (± 0.86)	74.78 (± 0.24)	0.869 (± 0.012)	0.724 (± 0.000)
	mT5 baseline	87.22 (± 0.67)	71.86 (± 1.07)	0.831 (± 0.005)	0.713 (± 0.011)
	mT5 masked	86.63 (± 1.05)	73.46 (± 0.89)	0.832 (± 0.020)	0.723 (± 0.007)
	BERTić baseline	90.52 (± 0.36)	75.48 (± 1.32)	0.880 (± 0.003)	0.751 (± 0.014)
	BERTić masked	90.22 (± 0.23)	75.29 (± 0.14)	0.877 (± 0.002)	0.744 (± 0.006)
Hierarchical	HAN baseline	88.65 (± 0.04)	72.94 (± 1.33)	0.847 (± 0.005)	0.711 (± 0.010)
	HAN Masked	89.96 (± 0.56)	73.93 (± 0.51)	0.868 (± 0.006)	0.733 (± 0.005)
Prompting LLMs	Direct prompting	54.22	57.33	0.497	0.576
	DSPy direct prompting	58.19	59.78	0.532	0.608
	DSPy COT	60.90	61.66	0.557	0.615
L2D using best PLMs	Confidence pick	93.04	78.20	0.911	0.781
	Average probabilities	92.94	78.09	0.908	0.776
	Majority vote	92.39	78.25	0.906	0.776
	mDeBERTa-v3 masked expert	92.33	77.49	0.900	0.761
	Longformer masked expert	92.25	76.63	0.903	0.772
	BERTić masked expert	91.48	76.55	0.896	0.760
Complete LLM deferral	HAN Softmax complete	89.83	74.39	0.870	0.733
	HAN Softmax complete + masked	90.43	74.21	0.876	0.733
	HAN complete deferral	91.56	75.64	0.888	0.758
	HAN complete deferral + masked	86.08	73.37	0.856	0.745
	mDeBERTa-v3 masked complete deferral	88.03	75.78	0.857	0.752
	Longformer masked complete deferral	88.23	73.95	0.872	0.756
	BERTić masked complete deferral	85.17	73.79	0.830	0.744
Selective LLM deferral	Longformer selective deferral	**93.44**	**79.56**	**0.913**	**0.799**
	Longformer selective deferral + masked	93.07	78.20	0.908	0.788
	mDeBERTa-v3 selective deferral	92.62	78.70	0.907	0.792
	mDeBERTa-v3 selective deferral + masked	92.68	77.94	0.903	0.769
Automatic baselines/controls	Document sentiment classifier	56.75	57.21	0.414	0.422
	Majority class	26.00	22.74	*NA*	*NA*

The top scores across each category (F1 and QWK, Seen vs. Unseen) for each Method have been underlined, while global high scores have been marked in bold. Selective LLM deferral rows use the best saved fixed gate for each prompt variant. Linear-regression and random-forest stacker rows are omitted from this seen/unseen breakdown because only aggregate policy metrics, not per-item predictions, were saved.

To contextualize our results, we first consider simple baselines. The Majority Class classifier (always predicting the most frequent class, “neutral”) performs reasonably well on that single class (F1 = 90.47% for Slovenian) but effectively zero on the others, yielding macro F1-scores of just 30.16% and 22.07% for Slovenian and Serbo-Croatian. These figures highlight the dataset's strong class imbalance and provide a natural lower bound against which more sophisticated models can be compared.

Additionally, the Document Sentiment Classifier performs slightly better but is still dramatically outperformed by all specialized models. This confirms that generic, document-level sentiment is an insufficient proxy for the nuanced, entity-targeted sentiment required by this task. Notably, neither of these simple baselines shows any significant performance difference between seen and unseen aspects, as they lack the capacity to learn aspect-specific features. These baseline results firmly establish the complexity of the task and the necessity of aspect-aware architectures, which we evaluate next.

### 6.1 Overall performance comparison

The overall comparison separates three questions that could otherwise be conflated. The first is whether strong supervised experts are still necessary for document-level entity-targeted sentiment classification, and which model properties matter most for this task. The second is whether several experts can be combined without an LLM. The third is whether an LLM is useful when it receives either the full prediction stream or only the low-confidence part of that stream.

To answer the first question, strong performance is not tied to a single architecture. Long-context, multilingual, text-to-text, language-specific, and hierarchical experts all provide competitive variants, with the best choice depending on language, metric, and whether the priority is aggregate performance, unseen-aspect robustness, or minority-class behavior. The strongest systems are supervised experts, or systems that keep a supervised expert at the center. Aspect masking is one of the clearest expert-model trends in Tables 3, 5. Across the main aggregate results, masked variants improve macro-F1 in 11/14 non-LLM paired rows, 10/12 fine-tuned or hierarchical rows, and all 6 Longformer, mDeBERTa-v3, and mT5 paired rows. The per-aspect analysis is more mixed, with positive mean ΔF1 in 8/18 family-language comparisons, indicating that the aggregate gains are not uniformly distributed across entities. We therefore treat masking as a strong practical recommendation for PLM/HAN-style expert models, rather than as a universal property of every model family or evaluation view. The input-conditioning rows also show that explicit extraction or LLM summarization is not necessary for strong performance, since several direct fine-tuning rows perform just as well as, if not better than context-management variants, suggesting that relevant sentiment cues are often available in the standard article-aspect input.

For the second question, the PLM-based L2D policies show that expert diversity helps, but only up to a point. Simple multi-expert policies improve over several single experts, while learned stackers do not clearly dominate, suggesting that the experts share many failure cases. This makes routing and adjudication more important than merely adding another similar expert.

Finally, prompting-only LLMs remain far below the fine-tuned PLMs and hierarchical models in both languages. The LLM L2D results are best read as deferral results rather than as evidence that the LLM can take over the task from the expert. Complete LLM deferral improves over standalone prompting because the LLM receives an expert prediction and confidence evidence, but it is unstable when applied to every item. Selective LLM deferral is more useful because it keeps high-confidence expert predictions unchanged and spends LLM calls only on the cases where the expert is least confident. This is why Tables 3–6 report complete deferral and selective deferral as separate strategy groups rather than as one hybrid family.

The table groupings follow the five evaluated approaches. *Embeddings, Fine-tuned PLMs, Hierarchical*, and *Prompting LLMs* describe the base model families. *L2D using Best PLMs* reports non-LLM expert policies such as majority vote, probability averaging, confidence pick, and shallow stackers. We separate *Complete LLM Deferral* and *Selective LLM Deferral* because they answer different deployment questions. *Complete LLM Deferral* reports systems where every item is sent to the LLM together with expert evidence. *Selective LLM Deferral* reports confidence-gated systems where only a fixed low-confidence fraction is sent to the LLM. The prompt-calibration choices used to select complete-deferral runs are tracked in the experimental provenance files, but they are not shown as table columns because the manuscript-level comparison is at the strategy level.

### 6.2 RQ1: expert model families and input conditioning

Our experiments show that end-to-end supervision and encoder-based approaches remain the strongest foundation for AspectBench. BGE-M3 document embeddings with an MLP are useful as a lightweight baseline, but they are consistently below the stronger PLM and hierarchical systems. This is most visible in Serbo-Croatian, where the best embedding row reaches 78.93 macro-F1, while several fine-tuned PLMs and HAN exceed 80 macro-F1. In Slovenian, embeddings are closer to the older XLM-R rows, but the strongest single-expert rows still come from fine-tuned encoders, especially SloBERTa and masked Longformer.

The PLM model set also clarifies the role of document length. Explicit compression or context management is not a prerequisite for strong performance. The XLM-R extractive and LLM-summary variants are informative, but they do not dominate the direct fine-tuning rows. For Slovenian, extractive XLM-R improves over truncated XLM-R, but LLM summaries are weaker and less stable; for Serbo-Croatian, truncated XLM-R remains stronger than extractive XLM-R and is close to the LLM-summary variants. At the same time, Longformer, mDeBERTa-v3, mT5, SloBERTa, and BERTić perform well without a separate summarization stage. Given the article-length distribution reported in Section 4.1.2, this suggests that the sentiment evidence is often available within the leading part of the article, repeated across the document, or recoverable from the standard article-aspect input. We therefore treat extractive filtering and LLM summarization as useful diagnostic variants rather than as necessary preprocessing steps for this benchmark.

In Slovenian, HAN outperforms the XLM-R variants in Table 3, but the newer end-to-end experts are competitive with it or stronger. This changes the practical interpretation of HAN. It is no longer the only strong long-document expert, but it remains useful because the expert-calibration and confidence-triage diagnostics in Supplementary Section S3, especially Supplementary Figures S3–S6, show very stable confidence behavior, making it a reasonable backbone for LLM deferral systems. In Serbo-Croatian, HAN is competitive but slightly below the simpler truncated XLM-R baseline and the strongest PLM experts. Its stable calibration again motivates its inclusion in the deferral analysis. Additional experiments would be needed to determine whether hierarchical aggregation with other PLM backbones yields the same calibration benefits.

The best expert family depends on the language and metric. Slovenian remains strongly affected by class imbalance, so improvements in negative and positive F1 matter even when accuracy changes little. Serbo-Croatian is less skewed and rewards models that preserve high positive and neutral performance while improving the negative class. Across both languages, however, the main conclusion is stable. Specialized supervised experts are the foundation on which the deferral systems must be built.

### 6.3 RQ2: aspect masking

Aspect masking is a strong candidate strategy for supervised expert models, but it should not be described as universally beneficial. At the table level, masked variants improve aggregate macro-F1 in 13 of 18 paired comparisons and aggregate QWK in 14 of 18. The pattern is stronger when the comparison is restricted to non-LLM expert rows, with masking improving macro-F1 in 11 of 14 non-LLM pairs and in 10 of 12 fine-tuned or hierarchical pairs. The reviewer-requested PLMs give the cleanest evidence, since Longformer, mDeBERTa-v3, and mT5 improve with masking in both languages.

The exceptions are important for interpretation. Slovenian BGE-M3 loses macro-F1 under masking while gaining QWK, which suggests fewer severe ordinal errors but weaker class balance. The language-specific encoders are also mixed, with Slovenian SloBERTa gaining QWK but losing a small amount of macro-F1, while Serbo-Croatian BERTić is stronger without masking. Complete LLM deferral is even less consistent, because the LLM can benefit from seeing the explicit target name when it is asked to adjudicate the text. The selective-deferral runs therefore combine a masked expert, which supplies the confidence gate, with masked and unmasked LLM prompt variants.

This distinction matters because the per-aspect paired analysis is more mixed than the aggregate tables, with only 8 of 18 family-language comparisons having a positive mean ΔF1 under masking. The safest conclusion is that masking is generally useful for PLM/HAN-style expert models, especially when generalization to unseen entities is important, but it remains an empirical input-conditioning choice that should be validated for each model family.

### 6.4 RQ3: learning-to-defer across expert models

The non-LLM learning-to-defer policies show that expert diversity helps, but only up to a point. In Slovenian, majority voting and probability averaging improve over the individual masked Longformer, SloBERTa, and HAN-XLMR experts, with majority vote reaching 76.35 macro-F1 and probability averaging reaching the highest QWK among these policies. However, the learned linear-regression and random-forest stackers do not improve over the simpler policies. This suggests that the experts share many hard cases rather than making independent, easily stackable errors. The qualitative analysis in Section 6.6 supports this interpretation: routed examples from different outcome types overlap in the same embedding neighborhoods, indicating that the difficult cases are not separated into clean model-specific failure regions.

Serbo-Croatian shows the same pattern with higher absolute scores. Confidence pick, probability averaging, and majority vote all exceed the individual masked experts, with confidence pick reaching 84.18 macro-F1 and 0.829 QWK. The stackers remain competitive but do not clearly dominate the simple policies. These results are important because they provide a non-LLM deferral baseline. If several strong experts fail on similar cases, then an LLM or a human analyst is only useful if the routing policy can identify a subset where the additional decision maker contributes something different.

### 6.5 RQ4: complete and selective LLM seferral

The LLM experiments separate three operating modes. Prompting-only LLMs are weak for this specialized task, even after DSPy optimization. Complete LLM deferral is stronger than prompting-only classification because the LLM receives the expert prediction and uncertainty evidence, but it is not stable enough to replace the expert on the full stream. When every item is sent to the LLM, the system also exposes already-correct high-confidence predictions to unnecessary revision.

Selective deferral changes the question from “Can the LLM classify every article-aspect pair?” to “Can expert confidence identify cases worth sending to the LLM, and can the LLM then improve them?” For Slovenian, the main setting uses the masked Longformer expert and an unmasked LLM prompt at a 10% gate, routing 774 of 7,740 test items. The full-test routed decision log improves over the Longformer expert from 75.88 to 77.34 macro-F1 and from 0.729 to 0.745 QWK, with 45 corrections and 16 degradations among routed items. For Serbo-Croatian, the main controlled diagnostic uses the masked mDeBERTa-v3 expert and an unmasked LLM prompt at a 30% gate, routing 3,723 of 12,407 items. This run remains competitive with the best multi-expert policies and improves QWK over the mDeBERTa expert, with 234 corrections and 190 degradations. The Serbo-Croatian Longformer selective run provides an additional check, improving over the Longformer expert at a 20% gate and reaching a larger correction/degradation margin of 233 to 157.

The table-level comparison also shows an important expert-backbone pattern: the Longformer-based selective-deferral row is the strongest Gemma selective-deferral row in both languages, including the Serbo-Croatian setting where it reaches 84.07 macro-F1 and 0.830 QWK in Table 5. We therefore treat the mDeBERTa-v3 curve as a controlled second Serbo-Croatian diagnostic rather than as evidence that mDeBERTa-v3 is uniformly the preferred selective-deferral backbone. The conservative interpretation is that long-context experts provide a strong base signal for this document-level task, while expert-specific calibration and prompt behavior still affect the best gate and the correction/degradation balance.

Figures 2, 3 show why the result is a deferral system result rather than an LLM replacement result. The blue dotted line corresponds to the expert prediction, i.e., the score obtained when the fine-tuned expert handles every test item alone. The orange oracle line replaces the lowest-confidence expert predictions with gold labels, so it measures the available headroom if the confidence gate really manages to find the hard cases. The green random-oracle line replaces the same number of randomly selected predictions with gold labels. When the orange curve is well above the green curve, confidence is selecting risk more effectively than chance. The red selective-LLM-deferral line shows the actual system improvement, where the lowest-confidence expert predictions are routed to the LLM and the remaining predictions are left unchanged.

**Figure 2:**
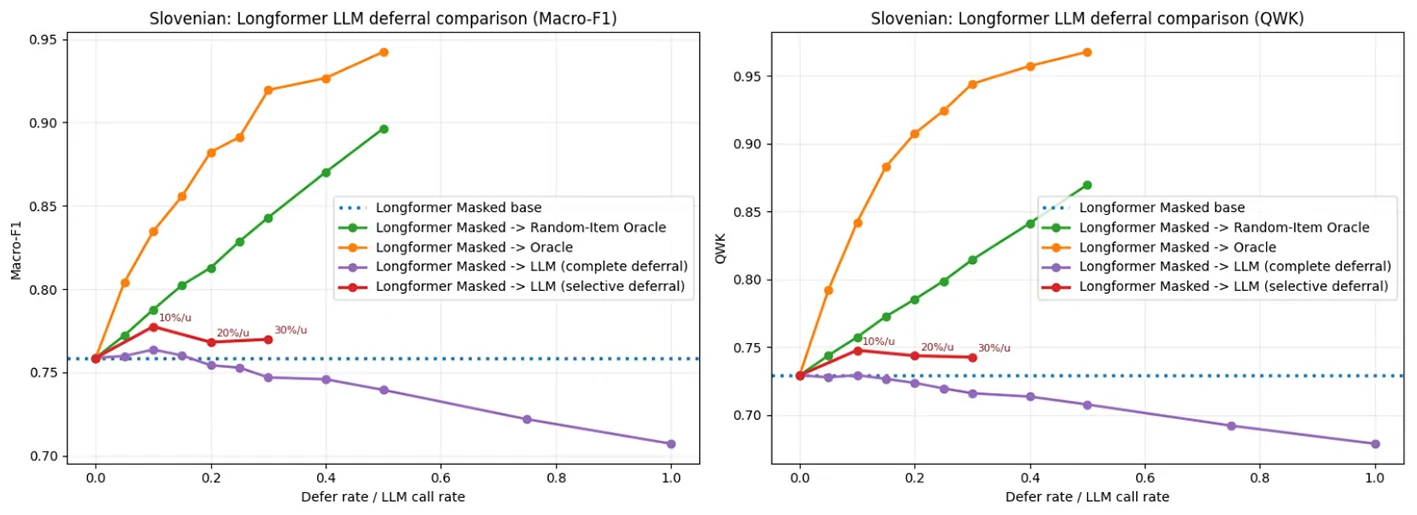
Complete vs. selective LLM deferral for the Slovenian masked Longformer expert. The oracle and random-oracle curves compare confidence-based routing against random routing, while the selective LLM curve shows the actual gated system. Selective deferral is most useful at the narrow 10% gate; complete deferral degrades when the LLM is applied to the full stream.

**Figure 3:**
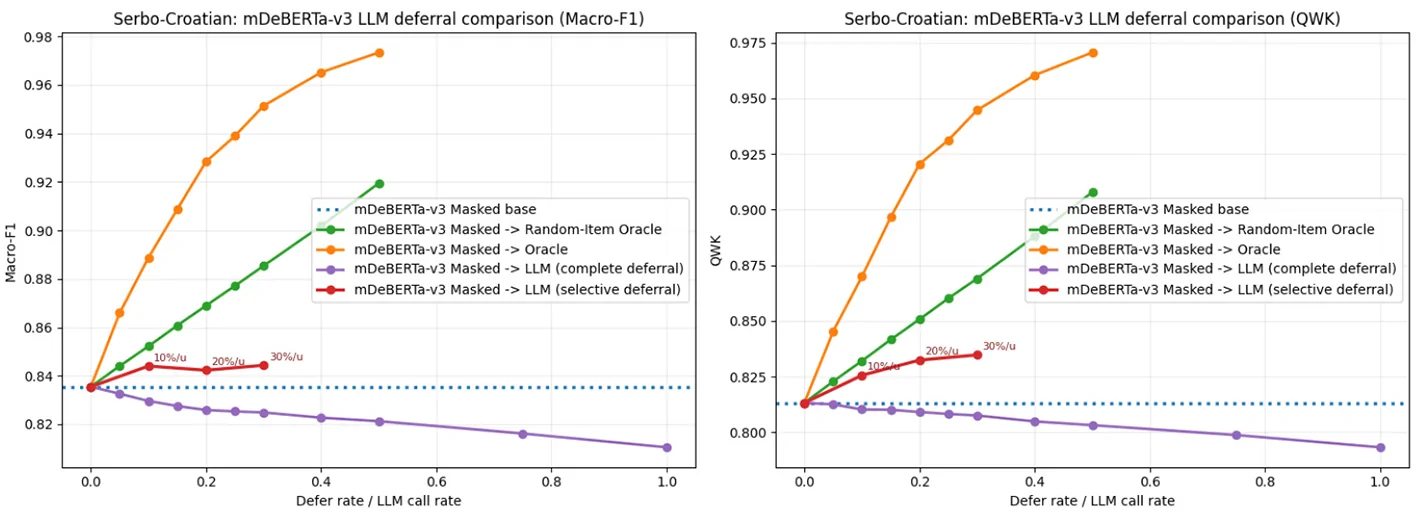
Complete vs. selective LLM deferral for the Serbo-Croatian masked mDeBERTa-v3 expert. Confidence-based oracle headroom remains above the random-oracle baseline, showing that uncertainty ranks difficult items. Selective deferral improves QWK more cleanly than macro-F1, indicating that several routed changes reduce ordinal error severity even when class-balanced F1 gains are modest. Complete deferral is less reliable as a full-stream replacement.

This diagnostic helps us separate two claims. First, the gap between the oracle and random-oracle curves shows that expert confidence is a useful risk-ranking signal, since the uncertainty gate is selecting a harder subset than random routing. Second, the red curve staying above or near the expert baseline at moderate gates shows that the LLM can recover part of the available headroom, even though it remains far below the oracle. This is deliberately weaker than claiming that confidence is perfectly calibrated: better routing policies may exist, especially for skewed class distributions, and should be treated as future work.

The calibration and polar-sentiment analyses in Supplementary Section S3 further show that the usefulness of uncertainty depends on class balance and on the number of routed positive and negative examples. Supplementary Figures S5, S6 compare expert confidence with empirical accuracy, while Supplementary Figures S7, S9, S12 restrict the deferral curves to sentiment-bearing cases only. When the oracle and random-oracle curves move closer together, especially on these smaller positive/negative subsets, uncertainty is a weaker routing policy and the LLM has less reliable headroom to exploit.

Complete deferral fails the deployment version of this test because it exposes already-correct high-confidence predictions to LLM revision. Selective deferral is therefore useful as controlled escalation, not as evidence that the LLM should replace the expert on the full-stream task.

The confidence-triage plots in Supplementary Figures S3, S4 provide the same evidence before the LLM is introduced. They sort items by expert confidence, remove the lowest-confidence fraction, and compute Macro-F1 and QWK only on the retained items. When retained-set scores rise, the removed items are disproportionately error-prone. A late drop at high defer fractions should not be read as confidence suddenly becoming useless; at that point, the retained subset can become small or class-impoverished, and Macro-F1 is especially sensitive because one weak minority-class estimate can dominate the average.

The triage plots should not be read as supporting a single architectural story such as “sharp confidence drop implies better selective deferral.” In Slovenian, Longformer and SloBERTa show sharper retained-set drops at high deferral fractions, while HAN-XLMR is more stable. In Serbo-Croatian, Longformer and HAN-XLMR are comparatively stable, whereas mDeBERTa-v3 and BERTić drop later. These patterns are useful diagnostics, but they are not conclusive mechanism proofs. The more robust claim from our study is that moderate-gated uncertainty ranking creates measurable deferral headroom, and selective LLM deferral is useful only when the LLM makes more corrections than degradations inside that headroom.

The class composition explains why the two languages behave differently. The Slovenian holdout set is highly skewed, with about 1.2% negative, 82.6% neutral, and 16.2% positive items. For the Slovenian Longformer, the bottom 30% confidence band shifts to about 2.3% negative, 65.5% neutral, and 32.3% positive. The gate is therefore strongly enriched for sentiment-bearing minority classes, especially positive and negative items. Past roughly the 25–30% gate range, retained-set Macro-F1 becomes less stable because the neutral-heavy remainder no longer reflects the full class mix. Serbo-Croatian is less skewed overall, with about 7.5% negative, 49.5% neutral, and 43.0% positive items. Its Longformer bottom 40% confidence band is about 11.5% negative, 50.3% neutral, and 38.2% positive, so the confidence gate looks more like a general difficulty filter, although negative cases are still overrepresented. These distributions do not prove that the deferred items are intrinsically ambiguous, but they do show that confidence identifies harder or more ambiguous regions of the task.

### 6.6 Qualitative error analysis

We further analyse the cases routed to the LLM by the selective gate. The goal is not to find a clean semantic cluster of “LLM-solvable” examples, but to understand what kind of difficulty expert uncertainty is selecting. The qualitative evidence supports a conservative interpretation in which selective deferral acts as an uncertainty-triggered adjudication mechanism, not as a topic-based router. Rescued cases, unresolved cases, and LLM-induced errors occur inside many of the same entity and topic neighborhoods. This overlap is consistent with the multi-expert results in Section 6.4: the strongest experts appear to encounter similar hard cases, rather than failing in wholly separate regions of the input space. The main difficulty is therefore local target attribution, i.e., deciding whether sentiment-bearing language in the article is actually directed at the target aspect.

In the selected hard-case cache, Slovenian uses the masked Longformer expert with an unmasked LLM prompt at a 10% gate, while Serbo-Croatian uses the masked mDeBERTa-v3 expert with an unmasked LLM prompt at a 30% gate. These settings route 774 Slovenian items and 3,723 Serbo-Croatian items. Figure 4 shows that model outcomes are mixed within the same embedding neighborhoods. This is useful negative evidence, since the LLM is not simply rescuing one separable topic cluster, and a simple topic router would not replace confidence-based gating. At the same time, the diagnostic has an important limitation: if the document embeddings do not separate the outcome types cleanly, topic models built on related representations may also blur distinct linguistic failure modes. We therefore use the UMAP and topic summaries as interpretive evidence for recurring templates, not as proof that the failure space has been fully explained.

**Figure 4:**
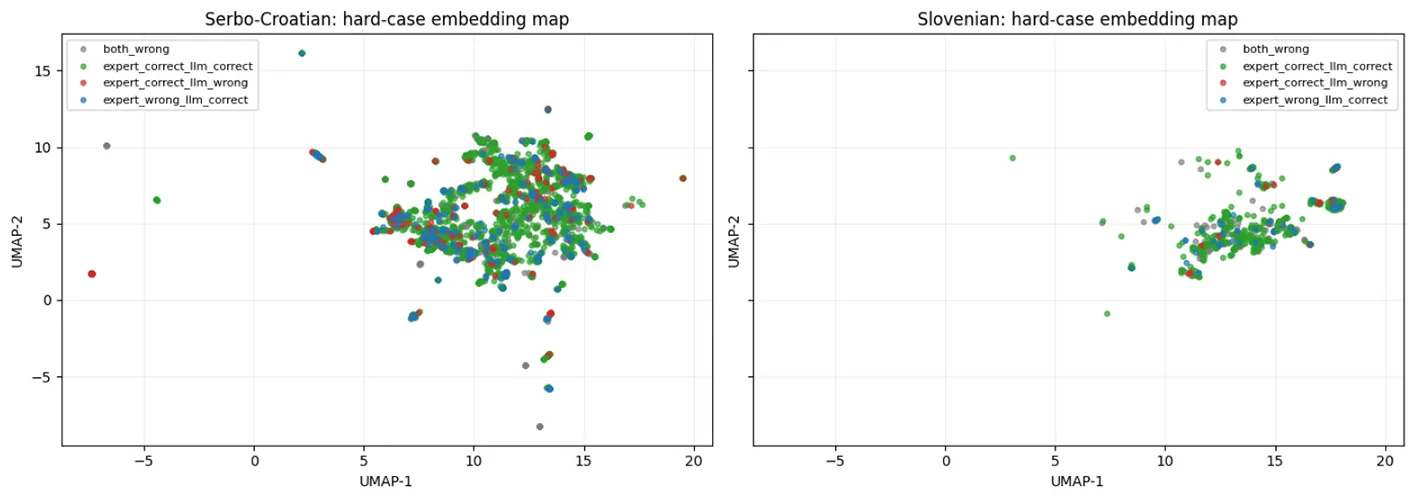
UMAP of LLM-routed hard cases for the selected selective-deferral gates. Slovenian uses the masked Longformer expert at a 10% gate, while Serbo-Croatian uses the masked mDeBERTa-v3 expert at a 30% gate. Outcome colors remain mixed within the same embedding neighborhoods, suggesting that the hard cases reflect overlapping target-attribution difficulties rather than a cleanly separable document topic or model-specific failure region.

The recurring templates are nevertheless interpretable. Routed examples include crisis or SMS-notification articles where negative event vocabulary is not necessarily negative sentiment toward the telecom operator, product-promotion articles where product positivity can be incorrectly transferred to the seller, and banking or merger articles where the target role is structurally complex. The LLM helps most when it can use local discourse cues to adjudicate the target relation; however, it fails when it overreads article-level affect, promotional language, or entity co-occurrence as target sentiment. Supplementary Section S4, “Qualitative and Industrial Diagnostics”, expands this analysis with class-conditioned UMAPs (Supplementary Figures S14, S15), routed-case outcome counts (Supplementary Table S1), and the multi-aspect diagnostic (Supplementary Figure S16). These analyses are exploratory, but they are consistent with the quantitative gate results because uncertainty identifies a hard band, and the LLM resolves a useful but limited fraction of that band.

### 6.7 RQ5: minimum viable set analysis

The minimum viable set analysis asks a different question from the model-comparison tables. If a media-monitoring team is starting a new annotation campaign, how quickly do useful signals appear as more labeled article-aspect pairs are added? Figure 5 shows that performance improves rapidly at small data fractions and then enters a slower diminishing-returns regime, but the answer depends on the criterion being optimized. For Slovenian, macro-F1 reaches within 10% of the full-data score at 30% of the training data and within 2–5% at 40%, making 40% a plausible planning point when near-final macro-F1 is required. For Serbo-Croatian, the corresponding macro-F1 thresholds are 10%, 30%, and 75%; the 30% point is therefore a useful initial stopping point if a within-5% macro-F1 band is acceptable, while stricter near-final performance requires substantially more data. The one-standard-error rule is more conservative, selecting 75% for Slovenian and the full 100% for Serbo-Croatian. The geometric knee appears much earlier (5% for Slovenian and 2% for Serbo-Croatian), showing where the first large gains saturate rather than where final-quality performance is reached.

**Figure 5:**
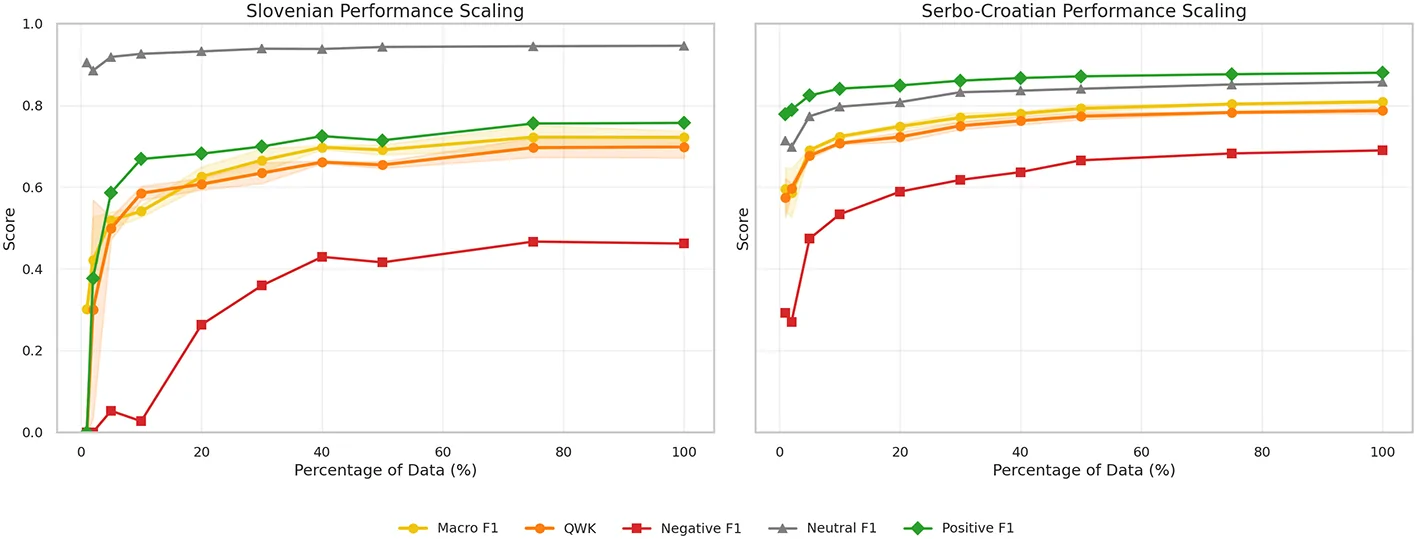
Minimum viable set analysis for the masked language-specific encoder experiments. Macro-F1 and QWK improve quickly at low training-data percentages and then show diminishing returns, but the apparent minimum viable set depends on whether the target is early curve saturation, near-full-data macro-F1, QWK, or a conservative variance-aware threshold. The per-class curves show why a single stopping rule is risky: Slovenian negative-class F1 remains data-hungry, while neutral F1 stabilizes early; in Serbo-Croatian, the less extreme class distribution allows positive and neutral F1 to scale more smoothly, but negative F1 still benefits from later data additions.

The practical interpretation is that there is no single minimum viable set for AspectBench. A small seed set can reveal the broad learning curve and uncertainty behavior, but minority-class behavior and high-confidence deployment decisions require substantially more data. This is consistent with the per-class panel in Figure 5 and the calibration and uncertainty diagnostics in Supplementary Figures S17, S18, where neutral-class behavior stabilizes earlier, while sentiment-bearing classes and calibration-sensitive decisions need more evidence. The MVS analysis therefore supports an incremental annotation strategy, starting with a small stratified pilot to estimate the curve and then expanding until the metric that matters for the deployment setting, such as macro-F1, QWK, minority-class recall, or uncertainty stability, reaches the required operating band.

## 7 Discussion

Our evaluation of diverse modeling methods on the AspectBench dataset provides a detailed view of the challenges and opportunities in document-level ABSA for less-resourced South Slavic languages. In this section, we provide additional interpretations of our findings in the context of our initial research questions and discuss their broader implications. The results answer the five research questions as follows.

- RQ1: Fine-tuned supervised experts are the most reliable foundation for AspectBench. Dense document embeddings are useful lightweight baselines, but the stronger PLM and hierarchical systems better capture target-specific sentiment. The input-conditioning experiments also show that explicit extraction or LLM summarization is not generally required, since standard article-aspect fine-tuning remains strong across the expanded PLM set.
- RQ2: Aspect masking is a useful default candidate for supervised expert models, especially PLM and HAN variants, because it often improves macro-F1, QWK, or unseen-aspect robustness without changing the model architecture. It should still be validated per model, since the embedding and language-specific encoder rows show that masking is not automatically superior, and LLM adjudication can benefit from seeing the explicit target name.
- RQ3: Non-LLM expert policies provide strong baselines but do not remove the hard-case ceiling. Majority vote, probability averaging, and confidence pick improve over several single experts, but shallow stackers do not clearly dominate, suggesting that the best experts share many failure points.
- RQ4: The LLM is most useful as a selective adjudicator rather than as a full-stream classifier. Prompting-only LLMs are weak, complete deferral is unstable, and selective deferral works when expert confidence identifies a low-confidence band where the LLM can make more corrections than degradations.
- RQ5: The minimum viable set analysis provides an annotation-planning lens rather than a universal data-size rule, because performance, minority-class behavior, calibration, and uncertainty stabilize at different rates.

### 7.1 Generalization and robustness

The seen/unseen split confirms that the test-only aspects are not a cosmetic detail. Unseen aspects account for 69.2% of the Slovenian holdout set and 59.1% of the Serbo-Croatian holdout set, so robustness to new entities is a central part of the benchmark. Most methods lose performance on unseen aspects, but the strongest expert-LLM deferral systems remain most useful.

In Slovenian, selective deferral with the masked Longformer expert and an unmasked Gemma-3 prompt gives competitive seen-aspect macro-F1, the highest seen-aspect QWK, and the strongest unseen-aspect results. In Serbo-Croatian, masked Longformer and masked mDeBERTa-v3 are the most useful expert backbones for selective deferral, while the unmasked Gemma-3 prompt gives the strongest LLM adjudication setting for unseen aspects.

The masking pattern is also visible in the seen/unseen tables. PLMs generally improve with masking, but selective deferral often works best when the expert is masked and the LLM prompt remains unmasked, allowing the LLM to see the explicit target name during adjudication. SloBERTa and BERTić are useful counterexamples to a universal masking claim because their masked and unmasked variants are close, and the preferred setting should be validated per model and language.

The practical conclusion is therefore to evaluate masking explicitly when unseen-entity generalization matters, not to assume it will always help.

### 7.2 Language-specific performance

Slovenian and Serbo-Croatian differ in ways that affect both expert modeling and deferral. Slovenian is much more imbalanced, with only 1.2% negative instances and 82.6% neutral instances in the holdout set. This makes macro-F1 sensitive to whether a model can recover the minority sentiment-bearing classes, and it also explains why the low-confidence band is enriched for positive and negative examples. Serbo-Croatian is more balanced, so confidence gating behaves less like a minority-class filter and more like a general difficulty filter.

These differences affect the best operating point. Slovenian benefits most from a narrow selective gate, with the 10% Longformer setting capturing a difficult subset without exposing too many already-correct expert predictions to the LLM. Serbo-Croatian tolerates a wider gate, with the mDeBERTa-v3 setting remaining useful at 30% and the Longformer setting giving a strong supplementary result at 20% (Supplementary Figure S11). The shared conclusion is not that the same gate rate transfers across languages, but that confidence-ranked routing is informative in both settings and should be tuned on validation data.

### 7.3 Industrial applicability

Our findings offer several practical insights for practitioners building industrial media monitoring systems.

- Strong expert models remain the core of the system because they are cheaper, more stable, and easier to validate than full-stream LLM adjudication. Based on these experiments, a conservative deployment path should keep the supervised expert as the default decision maker.
- Complete LLM deferral is not an attractive default, because it increases inference cost while also exposing high-confidence expert predictions to unnecessary LLM revisions.
- Expert confidence is useful for deferral because it ranks risk, with the lowest-confidence bands containing disproportionately difficult and sentiment-bearing cases.
- Selective LLM deferral is more practical. It keeps the expert prediction for most items, calls the LLM only on low-confidence cases, and produces a reasoning trace precisely where analysts are most likely to want additional evidence.
- The multi-expert policies show that simple non-LLM deferral strategies are also useful baselines. Majority voting, probability averaging, confidence picking, and stackers can stabilize performance, but their gains are limited when the expert models share the same hard cases.
- The selective LLM L2D setting is valuable because it targets those uncertain regions directly. This does not make the system production-ready by itself, but it gives a concrete operating point for media-monitoring workflows, where high-confidence expert predictions can be processed automatically and low-confidence cases can be escalated to an LLM or to a human analyst.

### 7.4 Limitations

Although our study compares most of the key methods used for aspect-based sentiment analysis tasks (Zhang et al., 2022), it still contains several limitations.

1. The Slovenian data cannot be released publicly because of privacy and commercial constraints, which limits external replication of the full multilingual benchmark.
2. The dataset was created in an industrial annotation workflow and does not include a corpus-wide retrospective inter-annotator agreement study.
3. Although we add Longformer, mDeBERTa-v3, mT5, and language-specific encoders, the model comparison is still not exhaustive; newer long-context encoders and stronger local LLMs may shift the absolute numbers. The final LLM experiments also focus on Gemma-3-27B-QAT after preliminary screening, so they should be read as evidence about this local-LLM setup rather than as a general claim about all current LLMs.
4. Selective deferral depends on two conditions. Expert confidence must identify genuinely difficult cases, and the LLM must be able to resolve a useful fraction of those cases. The present results satisfy these conditions for the tested settings, but the gate rate and prompt policy should be revalidated before deployment in a new language, domain, or annotation regime.
5. Uncertainty is a useful deferral signal in the tested settings, but alternative routing policies may be more effective for skewed class distributions. Other policies may also pair better with an LLM adjudicator once the deferral conditions are met. This should be investigated in future work.
6. Our experiments relied on a fixed set of reasonable hyperparameters without extensive tuning, meaning that further gains may be possible for each model.
7. The finding that many important sentiment cues appear in the introductory paragraphs may reflect an annotation or news-writing bias. This may be difficult to remove completely, given the trade-off between annotation volume, annotation quality, and annotation cost (Agnew et al., 2024).
8. The qualitative analysis is also exploratory. UMAP and topic models help interpret recurring failure templates and support the claim that several experts share similar difficult cases. However, the same overlap is also a limitation: if document embeddings do not cleanly separate rescues, residual errors, and LLM-induced errors, topic models derived from similar representations may inherit that limitation. These analyses therefore help describe the failure space, but they do not prove a causal mechanism.
9. Similarly, the multi-aspect diagnostic is based on known target mentions and should be read as a heuristic signal, not as full entity linking. We therefore use these analyses to support the interpretation of selective deferral, while keeping the quantitative gate-sweep and correction/degradation results as the primary evidence.
10. The minimum viable set findings are based on the masked language-specific encoder setup used in the progressive-data experiments, so the exact thresholds may be model-specific. The analysis also uses the naturally imbalanced data distribution rather than a separately balanced class-stratified target, meaning that different annotation goals could yield different optimal dataset sizes.

### 7.5 Future work

Our findings open several promising avenues for future research. The selective-deferral results suggest that more tightly integrated expert-LLM systems are a key next step. One could explore reinforcement learning techniques, such as Group Relative Policy Optimization (GRPO) (Shao et al., 2024), to use expert predictions, correction/degradation outcomes, or calibrated uncertainty as reward signals for adapting the LLM. Related fast-slow adaptation ideas, where prompt/context optimization and parameter updates are treated as complementary learning channels, may also be relevant (Tiwari et al., 2026). Our preliminary explorations in this area indicated that designing an effective policy and reward scheme for this task is non-trivial. Similarly, while direct Supervised Fine-Tuning (SFT) of the LLM on the ABSA task is another potential step, initial attempts suggested difficulties in achieving stable model convergence, warranting further investigation into more advanced fine-tuning strategies (Zhang et al., 2025). Extending AspectBench with more languages and different text domains would further enhance its value to the research community. Finally, a human-led study into the quality and trustworthiness of the generated rationales would be useful for understanding how selective deferral could support human-in-the-loop analysis pipelines.

Future work should first validate the selective-deferral pattern on additional document-level entity-targeted sentiment datasets, especially in related low-resource or morphologically rich languages. A second direction is to improve the routing policy. The present work uses expert confidence and fixed gate rates, but future systems could learn routing policies that combine confidence, entropy, disagreement across experts, and operational constraints such as LLM cost or analyst capacity. A third direction is to test stronger local LLMs as they become available. The current finding is not that the tested LLM should replace the expert, but that uncertainty-guided escalation creates a useful interface where future LLMs can be evaluated without changing the expert backbone.

The minimum viable set analysis points to a practical annotation-planning direction. Rather than treating dataset growth as an all-or-nothing requirement, future annotation campaigns can estimate how much labeled data is needed to reach a target performance band, stabilize minority-class behavior, or improve calibration. These estimates are criterion-dependent, so they should be used as planning aids rather than as universal thresholds.

## 8 Conclusion

In this paper, we addressed the need for robust, document-level Aspect-Based Sentiment Analysis systems in less-resourced languages, with a focus on news media monitoring. Our primary contribution is AspectBench, a new benchmark dataset for Slovenian and Serbo-Croatian that features long-form articles and a challenging test split designed to evaluate generalization to unseen aspects. The Serbo-Croatian subset of this benchmark is made publicly available to the research community.

Through a systematic empirical evaluation, we compared five modeling approaches, namely lightweight document embeddings, fine-tuned multilingual and language-specific PLMs, hierarchical networks, prompting-only local LLMs, and learning-to-defer policies that include non-LLM expert policies, complete LLM deferral, and selective LLM deferral.

Overall, AspectBench shows that document-level entity-targeted ABSA in South Slavic news remains challenging even for strong multilingual models. The expanded evaluation confirms that fine-tuned expert models are the strongest foundation for this setting, with Longformer, mDeBERTa-v3, mT5, and language-specific encoders adding important context to the original XLM-R and HAN comparisons. The input-conditioning results show that extraction and LLM summarization are useful diagnostics, but not necessary preprocessing steps for strong PLM performance. Aspect masking is generally useful for expert-model variants, especially across the new PLM baselines, although the effect should be described as an empirical tendency rather than a universal improvement.

The LLM results are clearest when complete and selective deferral are separated. Standalone prompting is not competitive, and complete LLM deferral is not reliable enough to replace the expert model on the full test stream. Selective LLM deferral is more promising when expert uncertainty identifies low-confidence cases, allowing the LLM to correct a useful fraction of them while leaving high-confidence expert predictions untouched. The qualitative analysis suggests that these improvements come from local target-attribution judgments in recurring news templates, not from broad topic routing. This makes selective deferral a practical and analytically useful mechanism for future media-monitoring systems because it reduces unnecessary LLM calls, exposes difficult cases for inspection, and provides a controlled way to test stronger LLMs as they become available.

## Data Availability

The Serbo-Croatian subset of AspectBench (HBS AspectBench 1.0; http://hdl.handle.net/11356/2356) is available from CLARIN.SI for non-commercial academic research under the CLARIN.SI Licence ACA ID-BY-NC-INF-NORED 1.0. Users must sign in, cite the dataset, notify the data provider before use, and must not redistribute the data. The released data include the full article text and the original aspect names. The Slovenian subset cannot be publicly released because of privacy and commercial restrictions agreed with our industrial partner.The code used for the experiments, including preprocessing scripts and model training pipelines, is available in our GitHub repository; https://github.com/nishan-chatterjee/aspect-based-sentiment-analysis. Additionally, the fine-tuned models are available in our Hugging Face collection (https://huggingface.co/collections/nishan-chatterjee/aspect-based-sentiment-analysis), while general information and instructions for using the released models are available in the accompanying Hugging Face repository (https://huggingface.co/nishan-chatterjee/aspect-based-sentiment-analysis).

## Footnotes

1 This is an empirical study of deferral behavior rather than a new formal L2D objective.

2 Unseen Aspects in this case refer to company names that are not present in the training data.

3 The Slovenian dataset contains a median of 306 articles per aspect, while Serbo-Croatian contains 296 articles per aspect.

4 We use “sl_core_news_sm” for Slovenian and “hr_core_news_sm” for Serbo-Croatian as the fast checkpoints from Spacy.

5 We tested Google Gemma-3's 27B QAT 4-bit checkpoint (Gemma Team and Google DeepMind, 2025), as well as GaMS-27B Q4_K_M checkpoints (Vreš et al., 2024), since they are faster to run while preserving most of the original quality, which is key for production deployment. However, since GaMS performed substantially worse than Gemma-3, we did not experiment further with it for summarization.

6 DSPy also allows for type specification, but for our use-case, we map the LLM predicted sentiment labels from “'negative,' 'neutral,' 'positive'” to “–1, 0, 1”.

7 The balanced subset was constructed by anchoring on the smallest class across both datasets, namely the negative class in the Slovenian split (507 total instances in the combined train-validation set). For the Slovenian balanced subset, we used all 507 negative samples and randomly downsampled the neutral and positive classes to match this count, resulting in 1,521 total instances. To preserve the original data proportions between languages, the per-class sample size for the Serbo-Croatian subset was scaled accordingly, resulting in 787 samples per class (2,361 total instances). Each of these language-specific balanced sets was then split into training (e.g., 405 per class for SL) and validation (e.g., 102 per class for SL) partitions. This entire sampling process was repeated three times with different random seeds to create three distinct balanced train-validation splits for our experiments.

8 We note that there were no failed tries during querying.

9 “Medium” creates around 2.5k LLM calls for prompt optimization while “heavy” creates around 5k LLM calls.

10 In complete LLM deferral, the LLM decides on whether to keep or override the PLMs predictions.

11 Longformer because it helped set a comparison with the Slovenian method, and mDeBERTa-v3 since it had the best masked multi-expert policy by macro-F1.

12 For the fine-tuned PLMs and Embedding-based models, we provide the standard deviation across 3 runs, where each run is performed on a randomly sampled train-val split. For LLMs, we query the model 3 times and consider the mode as the response; therefore, we do not offer standard deviation in this case.
