# Supplementary material for: Evaluating fine-tuned, embedding-based, and zero-shot models for aspect-based sentiment analysis in South Slavic news
Source: Front Artif Intell. 2026 Sep 18;9:1844418. doi: 10.3389/frai.2026.1844418 (PMC13630737; doi:10.3389/frai.2026.1844418)
Supplement: Supplementary file 1 [file Data_Sheet_1.pdf]

# Supplementary Material for: Evaluating Fine-Tuned, Embedding-Based, and Zero-Shot Models for Aspect-Based Sentiment Analysis in South Slavic News

Nishan Chatterjee[1,2,3*], Boshko Koloski[1,2], Antoine Doucet[3,4], Senja Pollak[1], Matthew Purver[1,5]

[1]Jožef Stefan Institute, Ljubljana, Slovenia.
[2]Jožef Stefan International Postgraduate School, Ljubljana, Slovenia.
[3]University of La Rochelle, La Rochelle, France.
[4]University of Ljubljana, Ljubljana, Slovenia.
[5]Queen Mary University of London, London, United Kingdom.

*Corresponding author(s). E-mail(s): nishan.chatterjee@univ-lr.fr;
Contributing authors: Boshko.Koloski@ijs.si; antoine.doucet@univ-lr.fr;
senja.pollak@ijs.si; m.purver@qmul.ac.uk;

## S1 Dataset Details

### S1.1 Example of Dataset Records

We represent our datasets as document-aspect pairs. Therefore, the same news article may appear multiple times in the dataset, once for each annotated target aspect, with a separate record identifier and potentially different sentiment labels. Listing 1 shows two simplified records derived from the same Serbo-Croatian financial news article. The article text is truncated for readability; the original dataset contains the full headline and article body. The English translation fields are included only for exposition and are not part of the soon-to-be-released dataset.

In each record, 'uuid" denotes the unique identifier of the document-aspect instance. The 'article" field contains the headline and article body, truncated here for readability. The 'aspect" field denotes the target company for which sentiment is annotated. The 'keywords" field lists tracked surface forms of the aspect name, including

wildcard patterns used during corpus construction. The ‘sentiment” field gives the sentiment expressed toward the target aspect, encoded as −1, 0, and 1 for negative, neutral, and positive sentiment, respectively. The ‘language” field gives the language label. The ‘published” field gives the publication timestamp in UTC; the printed article date may therefore differ depending on the local time zone. Finally, ‘country” indicates the country of publication.

```
{
"uuid": "11855",
"article": "bankama u srbiji bolje nego ikada ... neke banke poput sosijete zeneral banke i <aspect>erste banke</aspect> pohvalile su se da su postigle najbolji rezultat otkako posluju u srbiji. ...",
"translation": "Banks in Serbia are doing better than ever ... Some banks, such as Societe Generale Bank and Erste Bank, highlighted that they achieved their best result since they began operating in Serbia. ...",
"aspect": "Erste banka",
"keywords": ["erst* bank*", "ерст* банк*"],
"sentiment": 1,
"language": "Serbo-Croatian",
"published": "2017-05-02T22:00:00.000Z",
"country": "RS"
}

{
"uuid": "11854",
"article": "bankama u srbiji bolje nego ikada ... <aspect>telenor banka</aspect> ponovila je los rezultat iz prve godine poslovanja, a strucnjaci kazu da je to ocekivano zbog ogromnih ulaganja i da bi odrzivost ovog modela poslovanja mogla da se oceni posle cetiri, pet godina. ...",
"translation": "Banks in Serbia are doing better than ever ... Telenor Bank repeated the weak result from its first year of operation, while experts said this was expected because of substantial investments and that the sustainability of this business model could be assessed after four or five years. ...",
"aspect": "Telenor banka",
"keywords": ["telenor bank*", "теленор банк*"],
"sentiment": -1,
"language": "Serbo-Croatian",
"published": "2017-05-02T22:00:00.000Z",
"country": "RS"
}
```

**Listing 1** Simplified examples of two document-aspect records derived from the same article. The translation field is added only for exposition and is not part of the dataset.

Although both records originate from the same article text, their target aspects and corresponding sentiment labels differ. The first record is positive because the article

reports that Erste banka achieved its best result since entering the Serbian market. The second record is negative because Telenor banka is discussed as repeating a weak business result. This illustrates why the task is formulated as document-level entity-targeted sentiment analysis, that the sentiment must be predicted with respect to a specific target entity rather than assigned broadly to the document as a whole.

## S1.2 Preprocessing and Normalisation Pipeline

The raw data underwent a multi-step preprocessing pipeline to ensure reliability for model training and evaluation. The evolution of the dataset size through these steps is illustrated in Figure S1.

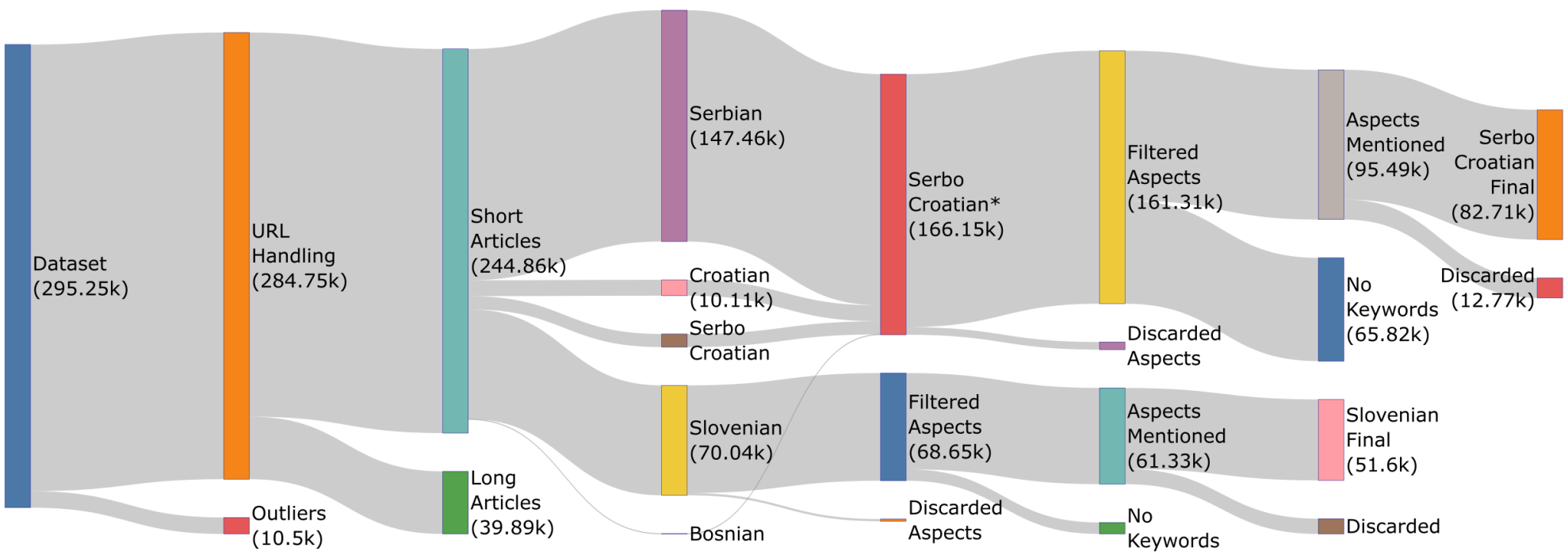


**Figure S1** Dataset Evolution Graph showing the reduction in dataset size through each preprocessing step.

1. **Outlier Removal (Initial Length-based):** We first address the extreme outliers in article length. We calculate the mean and standard deviation of character lengths using the raw article texts. Instances where the length exceeded the mean plus three standard deviations are removed, reducing the dataset to 291.2k items.
2. **URL Replacement and Secondary Outlier Removal:** URLs within the article texts were identified and replaced with a special “<URL>” token. This process occasionally introduced artifacts or highlighted previously masked outliers related to URL-heavy content. A second pass of outlier removal, using the same 3-standard-deviation threshold on character length post-URL replacement, further reduced the dataset to 284.8k items.
3. **Article Length Truncation:** To manage computational resources and align with typical input lengths for many transformer models, articles exceeding 5,000 characters were truncated to this maximum length. This step resulted in a dataset of 244.9k items.
4. **Language Identification and Filtering:** The original language tags provided with the articles exhibited some inconsistencies. To ensure greater accuracy, we employed FastText [1, 2] for language identification on each article. Articles associated with languages detected by FastText fewer than 100 times in the entire dataset

were removed to focus on more substantially represented languages, leaving 244.6k items.

5. **Language Subsetting (Serbo-Croatian (SC) and Slovenian (SL)):** Based on the refined language labels, we created two primary language subsets for AspectBench:

   - **Serbo-Croatian (SC):** This subset includes articles where the original language tag was "sr" (Serbian), "hr" (Croatian), "bs" (Bosnian), or "sh" (Serbo-Croatian) and FastText confirmed the language to be within this group.
   - **Slovenian (SL):** This subset includes articles where the original language tag was "sl" (Slovenian) and the FastText prediction was also "sl".

   After this step, the Serbo-Croatian subset contained approximately 166.2k items, and the Slovenian subset contained 70k items.
6. **Aspect Keyword Mention Filtering:** An essential factor in replicating realistic industrial tracking scenarios, where sentiment is typically analysed for entities explicitly mentioned, is to filter articles based on the presence of the target aspect. In collaboration with Kliping d.o.o., we compiled a dictionary mapping canonical aspect names to a list of their common inflections, colloquial forms, and relevant keywords. We then retained only those article-aspect pairs where at least one tracked form of the aspect was found within the article text using regular expression matching. Articles lacking any mention of their designated aspect's keywords were removed. This significantly impacted the Serbo-Croatian subset, where 40.81% (65.8k) of the items were removed, and the Slovenian subset, where 10.65% (7.3k) of the items were removed.
7. **Final Length and Keyword Count Normalisation:** To create more homogeneous subsets for focused analysis and reduce noise from extremely short/long documents or those with excessive keyword stuffing, a final filtering step was applied. We constrained article character lengths to be between 200 and 1,800 characters, and the number of keyword mentions for the target aspect to be between 1 and 5. These ranges were determined by analysing the distributions for each language and selecting percentiles that balanced data retention with consistency (specifically, the 20th-80th percentiles for Slovenian document length and keyword counts and the 29th-86th for Serbo-Croatian).

This resulted in the final AspectBench splits: the Serbo-Croatian subset containing 82.7k items and the Slovenian subset containing 51.6k items.

### S1.3 Sentiment Distribution

Figure S2 illustrates the sentiment distribution across the two language subsets.

## S2 LLM Prompts

This section details the evolution of prompts used for our Large Language Model (LLM) experiments, including any summarisation prompts, from our initial handcrafted versions to the final prompts generated by the DSPy MIPROv2 optimiser.

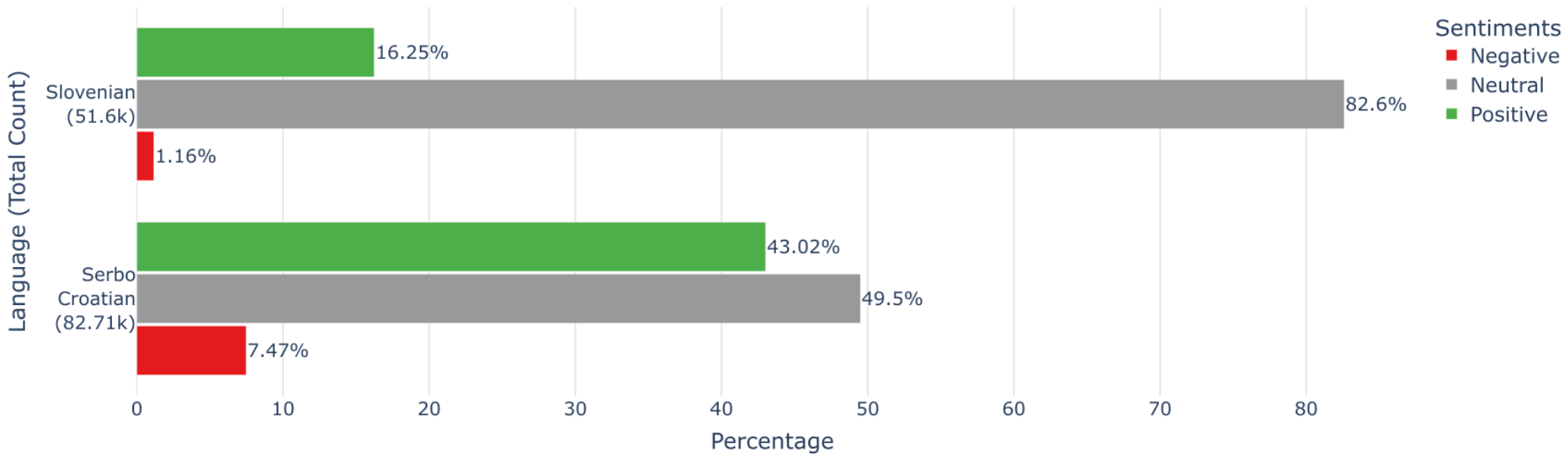


**Figure S2** Sentiment distribution across Slovenian and Serbo-Croatian subsets. Slovenian shows a significant skew towards neutral (82.6%) with minimal negative instances (1.16%), while Serbo-Croatian exhibits a more balanced distribution (positive: 43.0%, neutral: 49.5%, negative: 7.5%).

We present a combined summary and comparative analysis of the key changes for both the Slovenian and Serbo-Croatian datasets. The full, machine-readable JSON configurations for all optimised prompts will be made available in our public GitHub repository [1] for reproducibility.

```
Task: Create a concise summary focusing on the aspect "{aspect}" from the following news article. The aspect is marked with <aspect> </aspect> tags in the input. The summary should help understand the aspect's role and the sentiment towards it in the article.

Key Instructions:
1. Focus: Concentrate on the parts of the article discussing or closely related to the aspect "{aspect}" (marked with <aspect> tags). Include immediate context if it's important for understanding the situation or sentiment.
2. Content & Sentiment: Summarise the main points *about the aspect* and accurately reflect the sentiment (positive, negative, or neutral) conveyed towards it in the article.
3. Accuracy: Base your summary strictly on the facts presented in the provided article text. Do not add information not present in the text or misattribute details.
4. Language: Your response MUST be in {language_name}. Do NOT translate to English.
5. Conciseness: Keep the summary reasonably brief (e.g., around 3-6 sentences), capturing the essential points without excessive detail.

Article (in 'language_name'): 'article'
Aspect: 'aspect'
Answer (in 'language_name'):
```

**Listing 2** Aspect-based Summarisation Prompt

[1] currently set to private before acceptance

### S2.1 LLM Summarisation Prompt

We use the aforementioned summarisation prompt with the Gemma-3 27B QAT as well as GaMS-27B Q4_K_M models to generate aspect-focused summaries for the "XLM-RoBERTa with LLM-Assisted Summarisation" experiments. The placeholders "aspect", "language_name", and "article" were filled dynamically for each instance.

### S2.2 Evolution of Optimised Prompts

The following subsections trace the prompt evolution for each experimental setup, highlighting where the optimisation process converged on similar strategies for both languages and where it discovered divergent paths.

#### S2.2.1 Direct Prompting (Zero-Shot)

**Handcrafted Base Prompt:** Our initial prompt for both languages was highly structured, providing explicit, numbered instructions for the LLM to read the article, locate the aspect, and respond with a single, unadorned sentiment word.

**DSPy-Optimised Prompts & Analysis:** For this task, the optimisers for *both languages converged on an identical strategy, i.e., a drastic simplification of the instructions, namely:*

- **Key Changes:** The lengthy, step-by-step instructions and negative constraints (e.g., "Do NOT provide...") were completely removed.
- **Tonal Shift:** The tone shifted from conversational to a purely functional, declarative command (e.g., "Determine the sentiment ... Choose from..."). This indicates that for straightforward classification, a concise, machine-centric command is more effective than verbose, human-oriented instructions.

#### S2.2.2 Chain-of-Thought (CoT) Prompting

**Handcrafted Base Prompt (Conceptual):** For both languages, the conceptual base prompt simply added a "Reasoning" field to the direct prompt and instructed the model to "think step-by-step" before providing the final sentiment.

**DSPy-Optimised Prompts & Analysis:** This is where the optimised prompt strategies *diverged significantly*, showcasing the different local optima for each dataset.

The *Slovenian* prompt remained direct and functional, establishing a clear, two-part structure for the output without adding narrative flair.

```
Given an article and an aspect, first provide step-by-step
reasoning about the sentiment towards the aspect, considering its
context. Then, conclude with the final sentiment: 'negative',
'neutral', or 'positive'. Ensure the reasoning clearly and
logically leads to the final sentiment label.
```

**Listing 3** DSPy Optimised Slovenian CoT Prompt

In sharp contrast, the *Serbo-Croatian* optimiser invented a professional persona, introduced contextual stakes, and provided an illustrative example.

```
Given an article and an aspect, you are a critical analyst
responsible for evaluating media coverage. Your task is to
provide a detailed, step-by-step reasoning about the
sentiment towards the specified aspect, considering its
context within the article. This analysis will be used to
make strategic business decisions. Ensure that your
reasoning is clear, logical, and leads to a final sentiment
label of 'negative', 'neutral', or 'positive'. The accuracy
and thoroughness of your analysis are crucial for maintaining
the company's reputation and making informed decisions.

1. **Article**: Provide the full text of the article.
2. **Aspect**: Specify the aspect (e.g., a company, person) to
   analyse for sentiment.
3. **Reasoning**: Generate step-by-step reasoning about the
   sentiment towards the specified aspect, considering the
   context within the article. Identify key phrases, tone,
   and any direct or indirect references to the aspect.
4. **Sentiment**: Conclude with the final sentiment label
   ('negative', 'neutral', or 'positive') based on your
   reasoning.

Example:
- **Article**: "Telekom Srbija faces criticism over media
  pluralism concerns."
- **Aspect**: Telekom Srbija
- **Reasoning**: The article states that Reporters Without
  Borders (RSF) has expressed concern about the agreement
  between Telekom Srbija and Telenor, suggesting it could
  negatively impact media pluralism in Serbia. This indicates
  a negative sentiment towards Telekom Srbija as it is seen as
  potentially harmful to the media landscape.
- **Sentiment**: Negative
```

**Listing 4** DSPy Optimised Serbo-Croatian CoT Prompt

This divergence highlights that for tasks of moderate complexity, multiple effective prompting strategies exist. The optimiser found that a simple, clear instruction was sufficient for the Slovenian data, while a more elaborate, persona-driven scenario was more effective for the Serbo-Croatian data.

## S3 Complete and Selective Deferral Diagnostics

This section provides mechanism diagnostics for our selective LLM deferral experiments and strategies. The first question is whether expert confidence is a meaningful routing signal. Figures S3 and S4 sort items by model confidence, remove progressively larger low-confidence fractions, and report performance only on the retained items. If retained-set Macro-F1 and QWK improve as low-confidence items are removed, then

confidence ranks risk: the removed items are disproportionately difficult for the expert. A sudden drop at high defer fractions does not necessarily mean that confidence has stopped being useful. At high defer rates, the retained subset can become small or class-impoverished, and Macro-F1 is especially sensitive because minority-class F1 estimates can become unstable. The calibration plots answer a related but different question. They show whether the confidence values should be interpreted as calibrated probabilities or as practical risk-ranking scores.

The mechanism claim is therefore intentionally narrow, since selective deferral works only when two conditions hold at the same time: the expert's uncertainty ranking must identify a subset with real correction headroom, and the LLM must correct more items than it degrades inside that subset. The figures below test these two conditions separately. The confidence-triage and oracle/random-oracle curves test the routing signal, while the selective-LLM and gated-item replacement curves test whether the LLM contributes useful adjudication after routing.

## S3.1 Optimised Complete-Deferral Prompt Programs (Softmax vs. Uncertainty)

The optimised complete-deferral programs correspond to the first stage of the deferral exploration, where every document-aspect instance is sent to the LLM together with the article, the target aspect, the expert prediction, and supporting evidence from the expert model. Unlike selective deferral, these programs do not first gate low-confidence items. They therefore test whether the LLM can act as a full-stream adjudicator over the expert's predictions.

The optimised and calibrated prompt programs differ mainly in the type of expert evidence that is supplied to the LLM. In the **softmax-evidence** setting, the LLM receives the expert's predicted sentiment label and raw class probabilities. In the **uncertainty-evidence** setting, the LLM receives richer confidence and uncertainty information, including confidence, predictive entropy, and Monte-Carlo-dropout-derived uncertainty signals. In both settings, DSPy optimisation adapts the instruction structure separately for masked and unmasked variants.

For **softmax-evidence complete deferral**, the optimised prompt structure depends strongly on whether the aspect name is visible. In the unmasked Slovenian and Serbo-Croatian settings, the optimiser converges on a structured procedural checklist. The LLM is instructed to read the article, identify the target aspect, inspect the expert prediction and probabilities, compare the probabilities with textual evidence, and then produce a final sentiment judgment. This suggests that when the evidence consists mainly of raw softmax probabilities, the optimiser benefits from forcing the LLM to process the article, aspect, expert label, and probability evidence in a fixed order.

In the masked softmax-evidence setting, the optimised prompts become shorter and more direct. Since the target name is replaced by a placeholder, the LLM cannot rely on entity identity or world knowledge about the aspect. The task becomes closer to reasoning from local textual evidence around an anonymous target mention. In this setting, the optimiser tends to prefer concise instructions that ask for step-by-step reasoning and a final sentiment label, rather than a longer procedural checklist.

For **uncertainty-evidence complete deferral**, the optimised programs move toward a different form of guidance. Instead of relying only on direct instructions, the optimiser often introduces an in-context reasoning template that demonstrates how to combine article evidence with uncertainty signals. This is especially visible in masked variants, where both languages converge toward example-driven prompts. The example acts as a template for interpreting confidence, entropy, and uncertainty alongside the article text when the concrete aspect name is unavailable.

The unmasked uncertainty-evidence prompts show more language-specific variation. For Slovenian, the optimised program remains comparatively direct, closer to the shorter masked softmax prompts. For Serbo-Croatian, the optimiser embeds a fuller example into the instruction, showing how the LLM should reason through the relation between the article, the aspect, the expert prediction, and the uncertainty evidence. We treat this variation as a prompt-program diagnostic rather than as a standalone claim about language-specific prompting.

Overall, the complete-deferral prompt comparison supports two observations. First, the optimiser does not find one universal prompt structure for expert-guided LLM adjudication. The preferred structure changes with the evidence type and with whether the aspect name is visible. Second, uncertainty-evidence prompts tend to require more explicit reasoning support than softmax-evidence prompts, because the LLM must interpret confidence, entropy, and disagreement signals in addition to the article itself. This helps explain why complete deferral is more informative as a diagnostic than as a deployment strategy: optimised prompts can improve the LLM's use of expert evidence, but applying the LLM to every instance still exposes many already-correct high-confidence expert predictions to unnecessary revision.

## S3.2 Optimised Selective-Deferral Prompt Programs

The optimised selective-deferral programs use the same input and output schema across the selected runs. Each prompt receives the article, the target aspect, the primary expert identity, the primary expert prediction, the primary probabilities, uncertainty information such as confidence, entropy, vote distribution, and threshold hints, auxiliary expert predictions and uncertainties, and a routing-context field explaining why the item was selected for possible deferral. The output schema asks for a short evidence-based reasoning trace, an action from `keep_plm`, `override`, or `abstain_uncertain`, and a final sentiment label.

The optimised instructions differ in useful but modest ways. The Slovenian Longformer program remains a compact procedural checklist: review the article and aspect, assess the primary prediction, compare auxiliary experts, inspect article evidence, and then keep, override, or abstain. The Serbo-Croatian Longformer program keeps the same audit structure but adds a conservative in-context example where the primary Longformer prediction and auxiliary experts agree on a neutral label. This makes the keep decision explicit and discourages unnecessary overrides. The Serbo-Croatian mDeBERTa-v3 program instead uses an example where the primary mDeBERTa-v3 prediction is neutral, but article evidence and auxiliary experts support a positive override. This makes the override case more salient and helps explain why the two Serbo-Croatian selective-deferral backbones can show different macro-F1 and QWK

behaviour even under the same general DSPy setup. We treat these differences as prompt-program diagnostics rather than as a separate claim of prompt novelty.

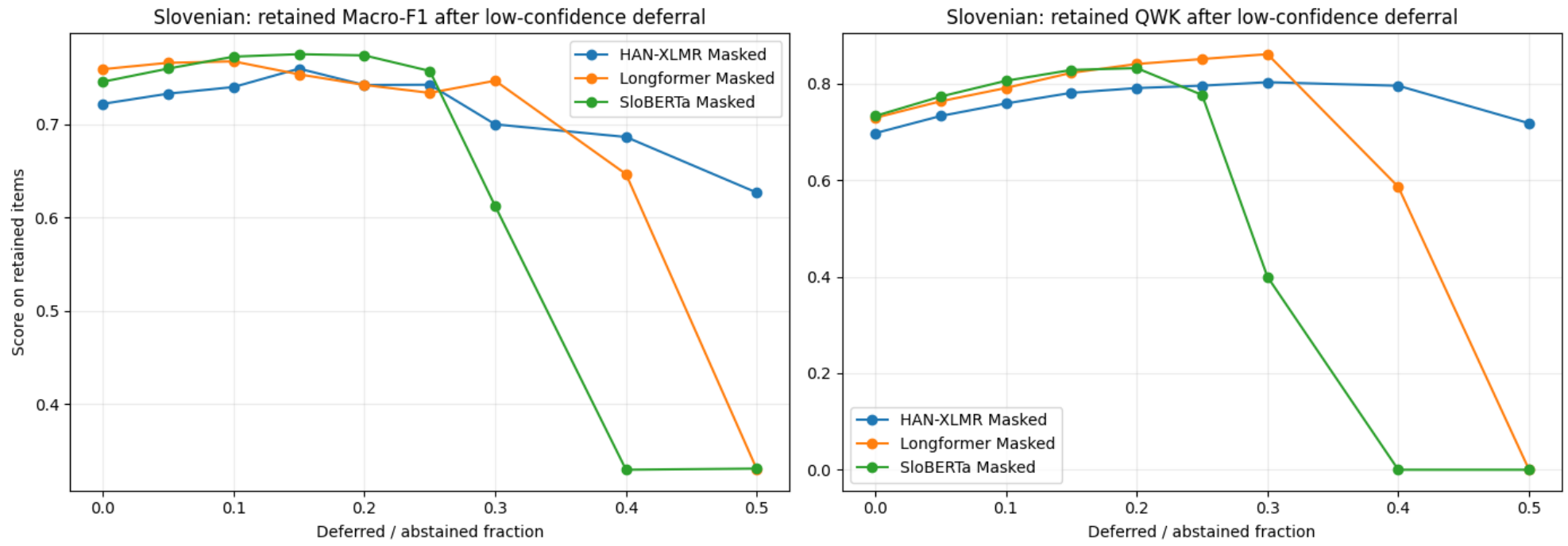


**Figure S3** Confidence triage for Slovenian experts. Retained-set scores generally improve when the lowest-confidence items are removed, which supports using expert confidence as a routing signal for selective deferral. The later drops for Longformer and SloBERTa should be read cautiously because high deferral fractions leave a smaller and more class-skewed retained set.

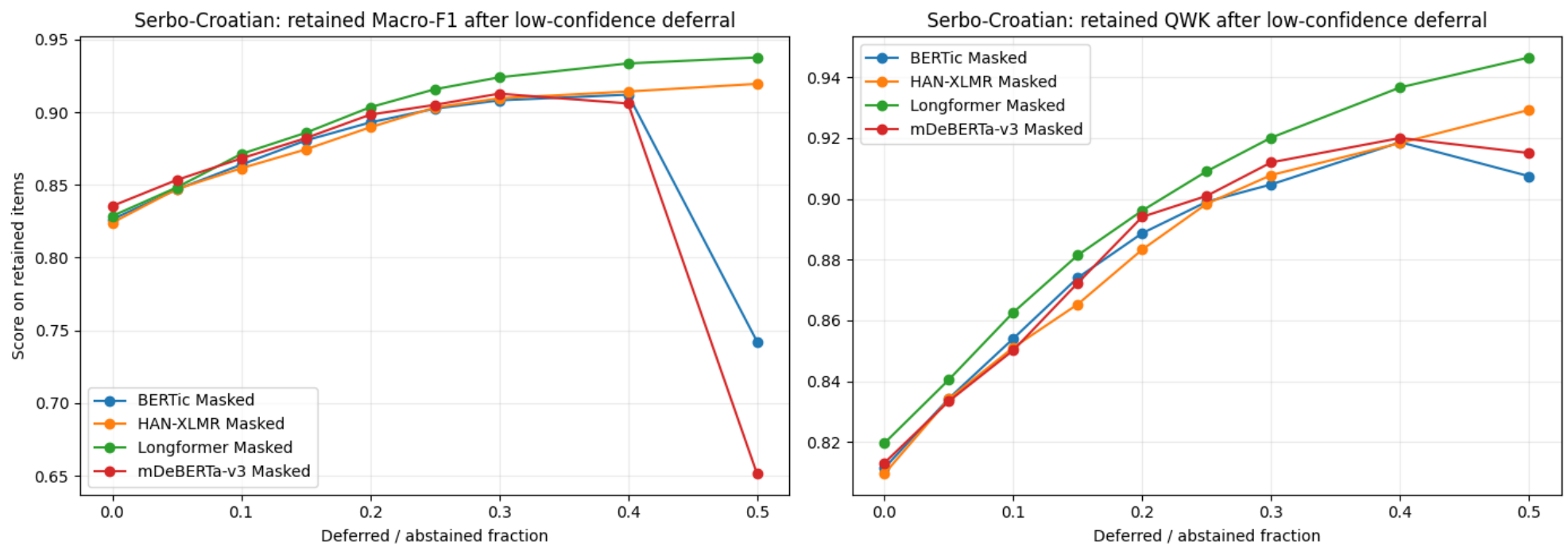


**Figure S4** Confidence triage for Serbo-Croatian experts. The retained-set performance rises across moderate deferral fractions, indicating that low-confidence items are disproportionately difficult and can be targeted by selective LLM deferral. The curves are smoother than in Slovenian for several experts, consistent with the less extreme class distribution.

The low-confidence bands differ by language. The Slovenian holdout set is highly skewed, with approximately 1.2% negative, 82.6% neutral, and 16.2% positive items. For the Slovenian Longformer expert, the bottom 30% confidence band contains approximately 2.3% negative, 65.5% neutral, and 32.3% positive items. Low confidence is therefore strongly enriched for sentiment-bearing minority classes, especially positive and negative examples. This helps explain why retained Macro-F1 can become

unstable beyond roughly 25-30% deferral: the remaining retained set is increasingly neutral-heavy, while the deferred band contains much of the minority-class difficulty.

Serbo-Croatian is less skewed, with approximately 7.5% negative, 49.5% neutral, and 43.0% positive items in the holdout set. For the Serbo-Croatian Longformer expert, the bottom 40% confidence band contains approximately 11.5% negative, 50.3% neutral, and 38.2% positive items. In this case, confidence triage behaves more like a general difficulty filter than a class-skew filter, although negative cases are still over-represented among low-confidence items. The triage plots therefore do not prove that the deferred items are intrinsically ambiguous, but they show that confidence is an empirically useful signal for selecting harder or more ambiguous cases.

The shape of the triage curves is not by itself a sufficient explanation for selective-deferral success. Some experts show a sharp retained-set drop at high deferral fractions, while others remain stable over the same range. This means we should not overinterpret a single curve shape as the mechanism. What matters for the deployed selective system is whether the gate creates headroom at a moderate call rate and whether the LLM can use the routed evidence to produce more corrections than degradations.

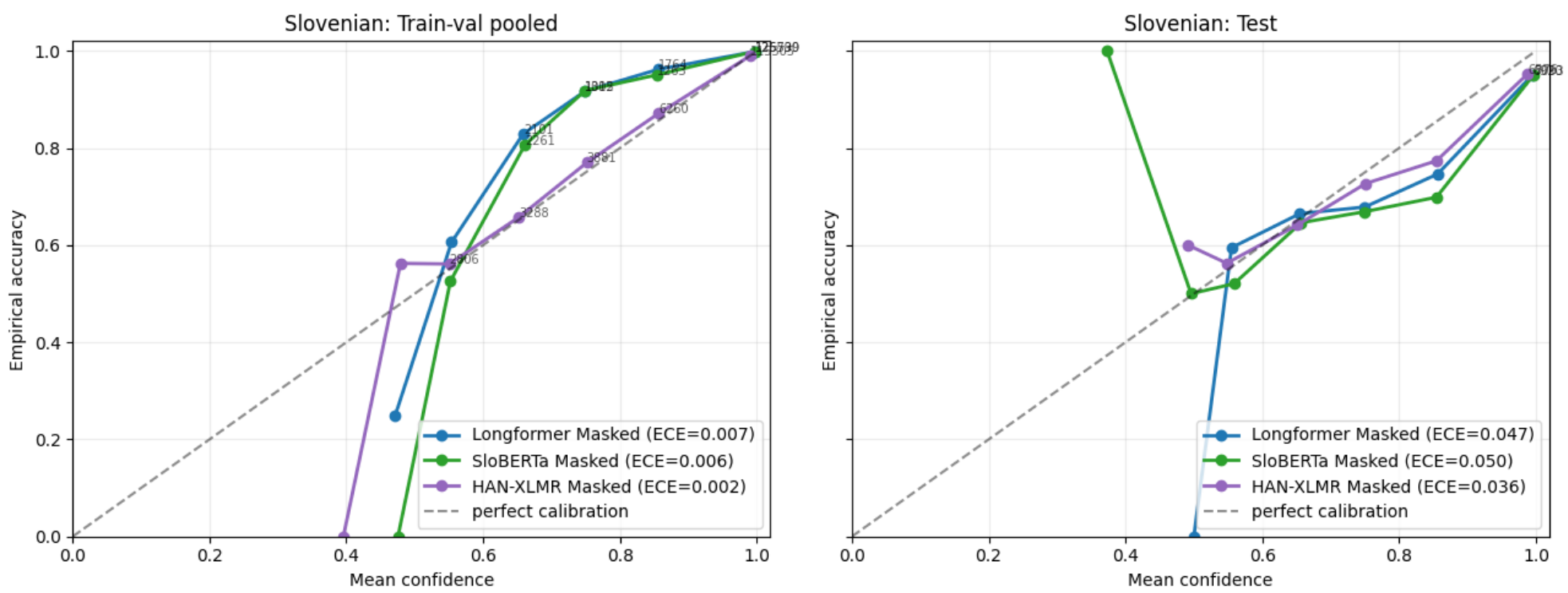


**Figure S5** Expert calibration curves for Slovenian. The curves support confidence-based risk ranking, but also show why the paper treats confidence as a routing signal rather than as a perfect probability estimate. Miscalibration is most consequential for selective deferral when the routed subset contains few positive or negative examples.

The next figures expand the complete-versus-selective deferral comparison from the main text. In the complete/selective curves, the expert baseline is the score without LLM intervention. The oracle curve replaces the lowest-confidence expert predictions with gold labels and therefore estimates the maximum headroom available under confidence-based routing. The random-oracle curve replaces the same number of randomly selected items and provides a routing control. When the oracle curve is far above the random-oracle curve, expert confidence is selecting harder cases than random choice. If the two curves are close, confidence gating is only weakly better than random escalation for that subset. The selective LLM curve then shows the actual

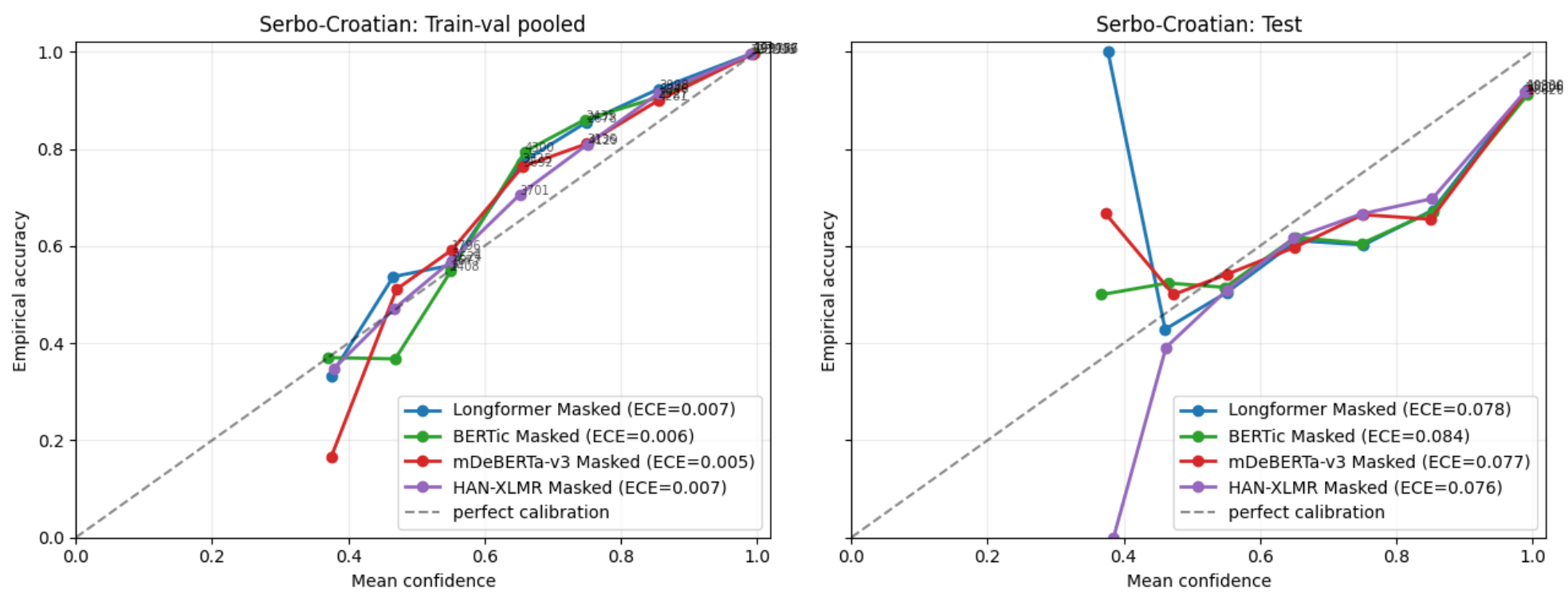


**Figure S6** Expert calibration curves for Serbo-Croatian. Confidence is informative for identifying difficult cases, but the calibration is not perfect and should be interpreted together with the triage and gate-sweep results.

routed system. Scores above the expert baseline indicate useful deferral, while the distance from the oracle curve is the remaining gap between current LLM adjudication and the best possible correction of the selected hard cases.

The sentiment-bearing panels restrict the evaluation to positive and negative cases, where target sentiment is explicit but often harder to attribute correctly. These panels are useful precisely because they are less forgiving: the class counts are smaller, calibration errors matter more, and the oracle and random-oracle curves can move closer together. For the Slovenian Longformer polar subset in Figure S7, the narrower gap between the oracle and random-oracle curves means that uncertainty is a weaker policy than in the full-set view, usually because model confidence no longer separates errors from correct predictions as cleanly. This is consistent with the minimum viable set analysis, where sentiment-bearing classes, especially Slovenian negative cases, need more data before their behaviour stabilises.

The gated-item replacement panels compare the selective LLM decision with keeping the expert prediction on the same routed items and with simple replacement controls. These panels answer the second mechanism question: after uncertainty has selected a hard subset, does the LLM improve those routed decisions? Improvements over keeping the expert prediction show that the LLM is not merely changing labels on a difficult subset, but is adding some evidence-based adjudication.

## S4 Qualitative and Industrial Diagnostics

The qualitative plots are exploratory. They help interpret why selective deferral helps, but they should not be read as independent proof of the mechanism. We organise the routed cases by language, target, and recurring news templates, while rescues, residual errors, and LLM-induced errors remain mixed inside the same neighbourhoods. This overlap supports the main-text interpretation that the strongest experts share similar hard cases and that the LLM helps mainly with local target attribution rather than broad topic routing or unsupported world-knowledge lookup. It is also a limitation

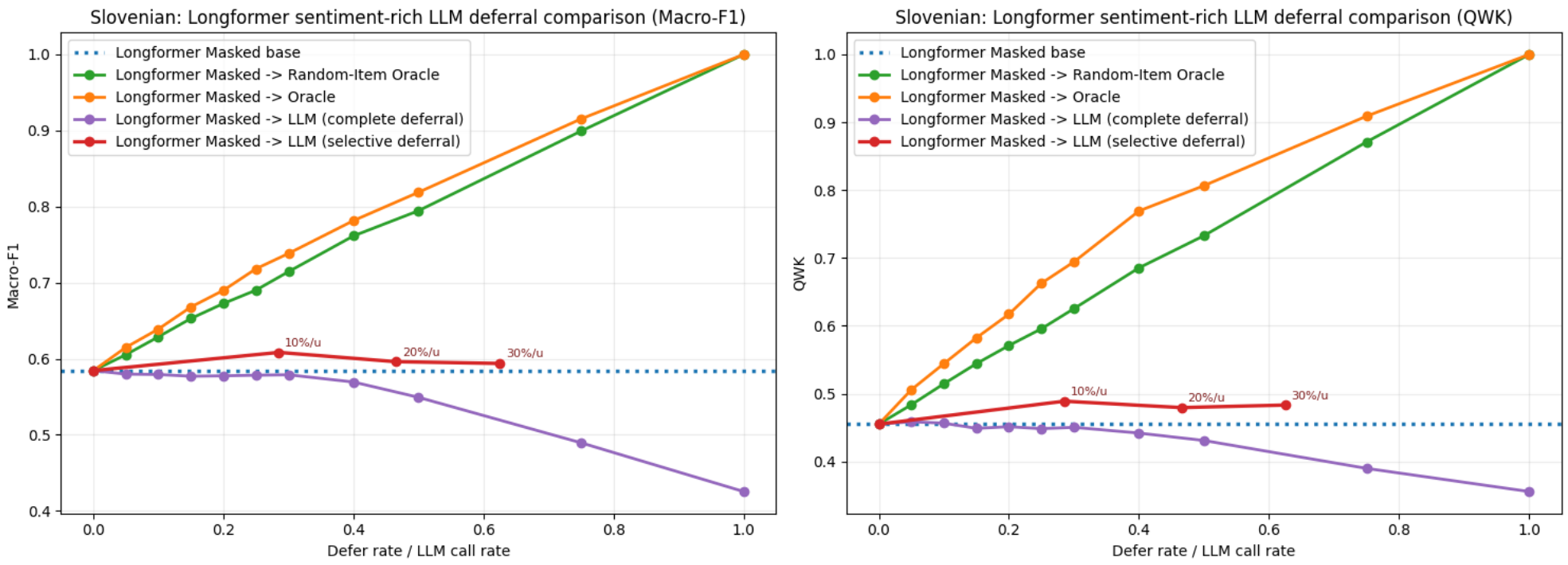


**Figure S7** Slovenian Longformer complete versus selective deferral on sentiment-bearing positive and negative cases. The oracle headroom is large, but the gap between oracle and random-oracle is less stable than in the full-set view because the polar subset is small and class-skewed. Selective LLM deferral recovers only part of the available headroom, reinforcing that the LLM is helpful on selected cases but not a complete solution.

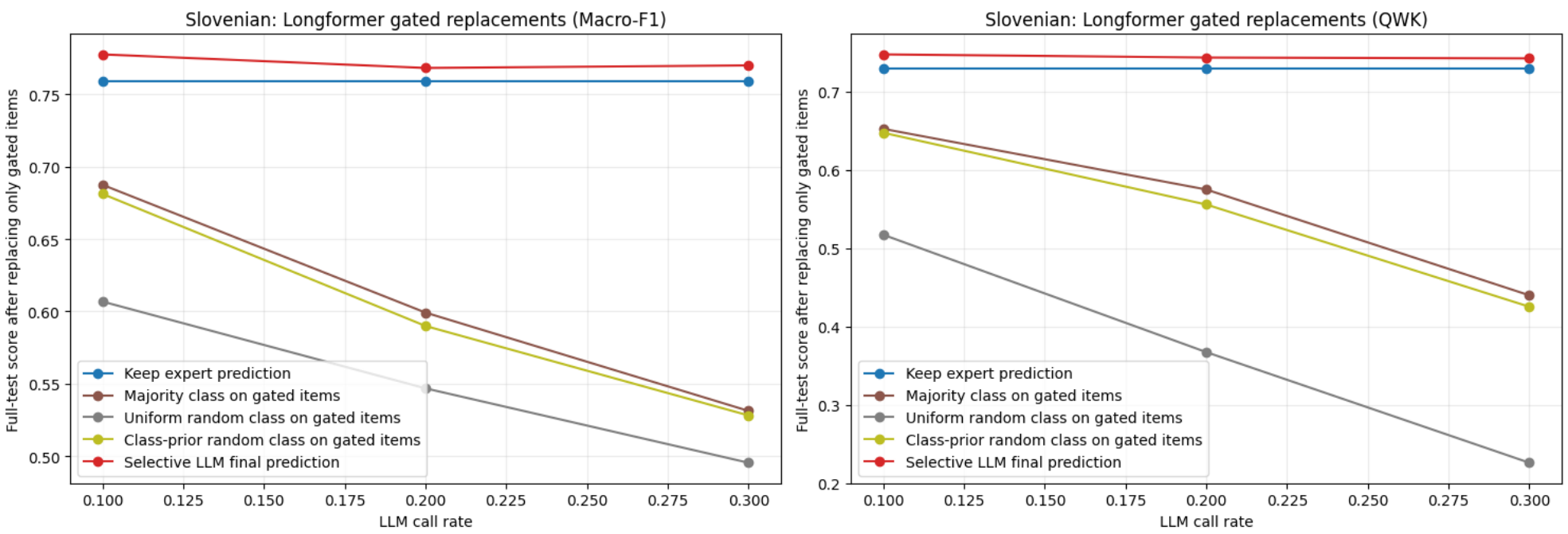


**Figure S8** Slovenian Longformer gated-item replacement scores. The comparison is restricted to the same routed items at each gate, so improvements over keeping the expert prediction and simple replacement controls indicate that the LLM performs meaningful adjudication on low-confidence cases.

of the diagnostic: if the document-embedding space does not cleanly separate these outcomes, then topic summaries built from related representations may also merge distinct linguistic failure modes.

The selected qualitative cache uses different gate rates for the two languages because the best stable operating point differs by language. Slovenian uses the masked Longformer expert with an unmasked LLM prompt at a 10% gate. Serbo-Croatian uses the masked mDeBERTa-v3 expert with an unmasked LLM prompt at a 30% gate. These settings match the main-text comparison: Slovenian benefits most from the narrower gate, while Serbo-Croatian remains stable under wider routing.

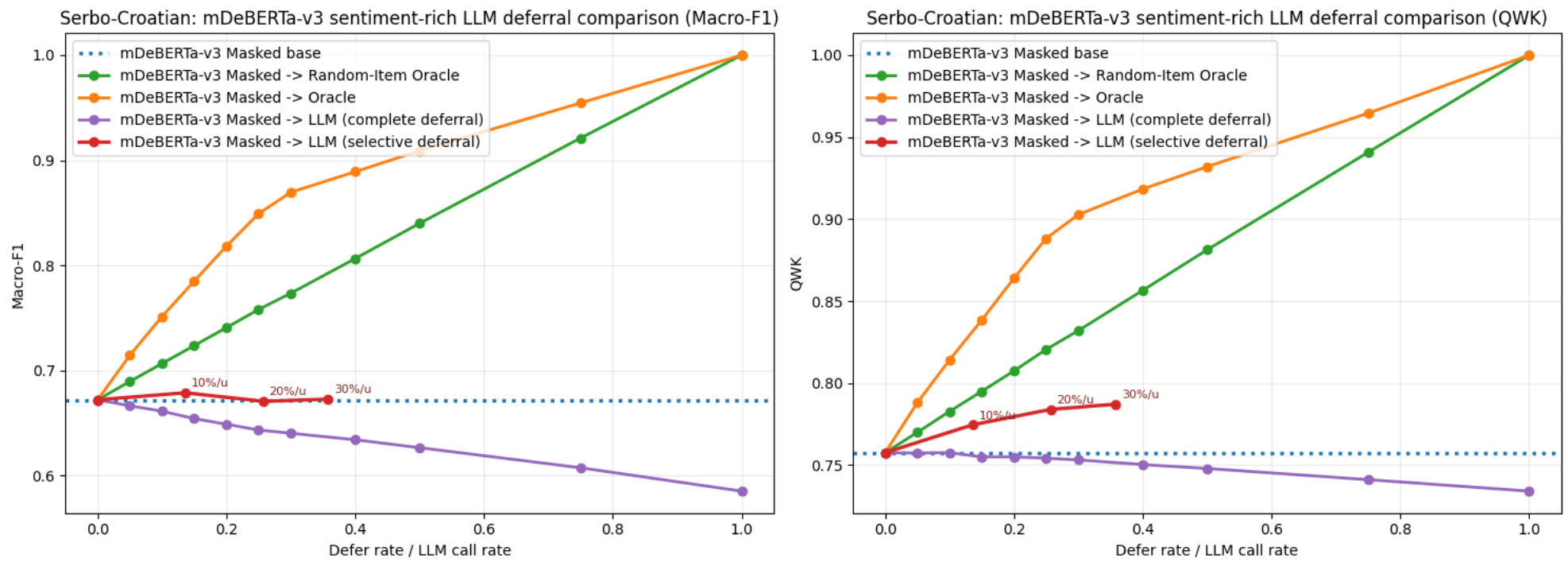


**Figure S9** Serbo-Croatian mDeBERTa-v3 complete versus selective deferral on positive and negative cases. The sentiment-bearing subset shows substantial headroom, but the macro-F1 curve is flatter than the QWK curve because class-balanced F1 is sensitive to which positive and negative items are changed, whereas QWK rewards reductions in ordinal error severity. Selective deferral remains a partial and gated improvement rather than an oracle-level replacement.

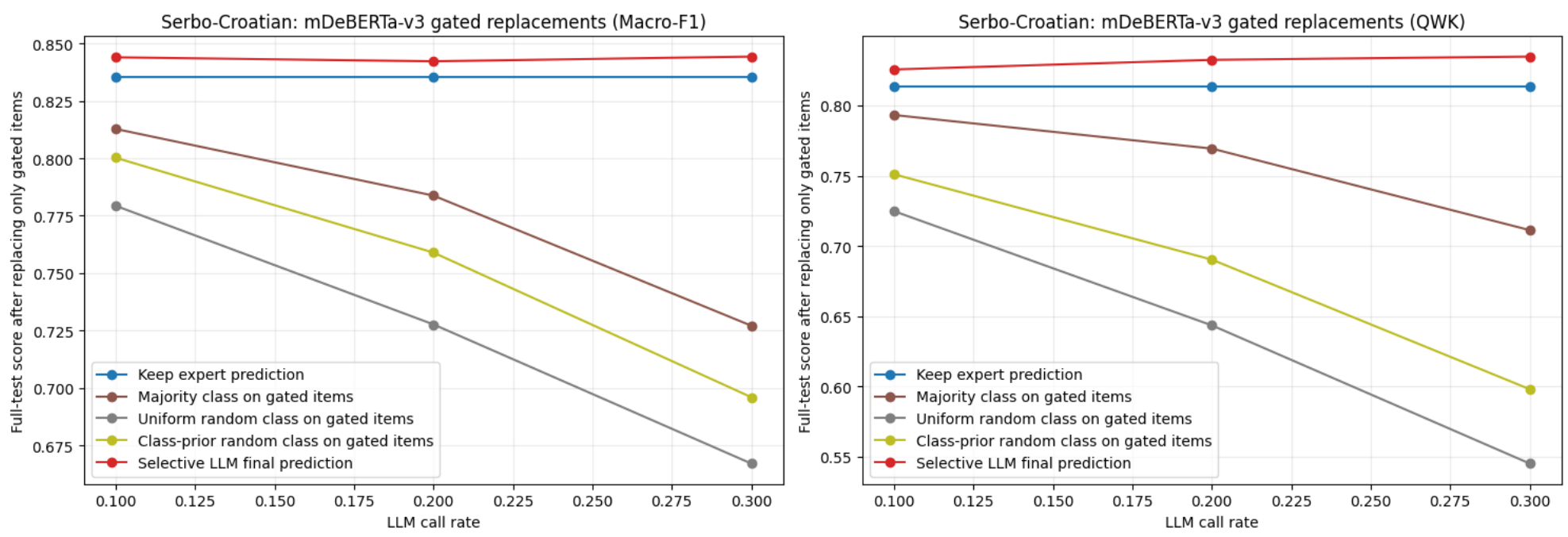


**Figure S10** Serbo-Croatian mDeBERTa-v3 gated-item replacement scores. The selective LLM decision remains competitive with, and often above, keeping the expert prediction on the routed subset, which supports the interpretation that the LLM contributes useful adjudication rather than merely changing labels through a naive replacement policy.

| Language | Gold class | Routed n | Expert wrong | LLM rescues | Expert correct | LLM-induced errors |
|---|---|---|---|---|---|---|
| Serbo-Croatian | Negative | 486 | 170 | 61 | 316 | 12 |
| Serbo-Croatian | Neutral | 1900 | 579 | 102 | 1321 | 167 |
| Serbo-Croatian | Positive | 1337 | 384 | 139 | 953 | 11 |
| Slovenian | Negative | 21 | 8 | 4 | 13 | 0 |
| Slovenian | Neutral | 440 | 127 | 18 | 313 | 15 |
| Slovenian | Positive | 313 | 115 | 29 | 198 | 1 |

**Table S1** Class-conditioned routed-case outcomes for the qualitative cache. The LLM rescue rate is higher on sentiment-bearing classes than on neutral, while LLM-induced errors concentrate more strongly in neutral cases. Slovenian negative routed cases are few, so that row should be interpreted cautiously.

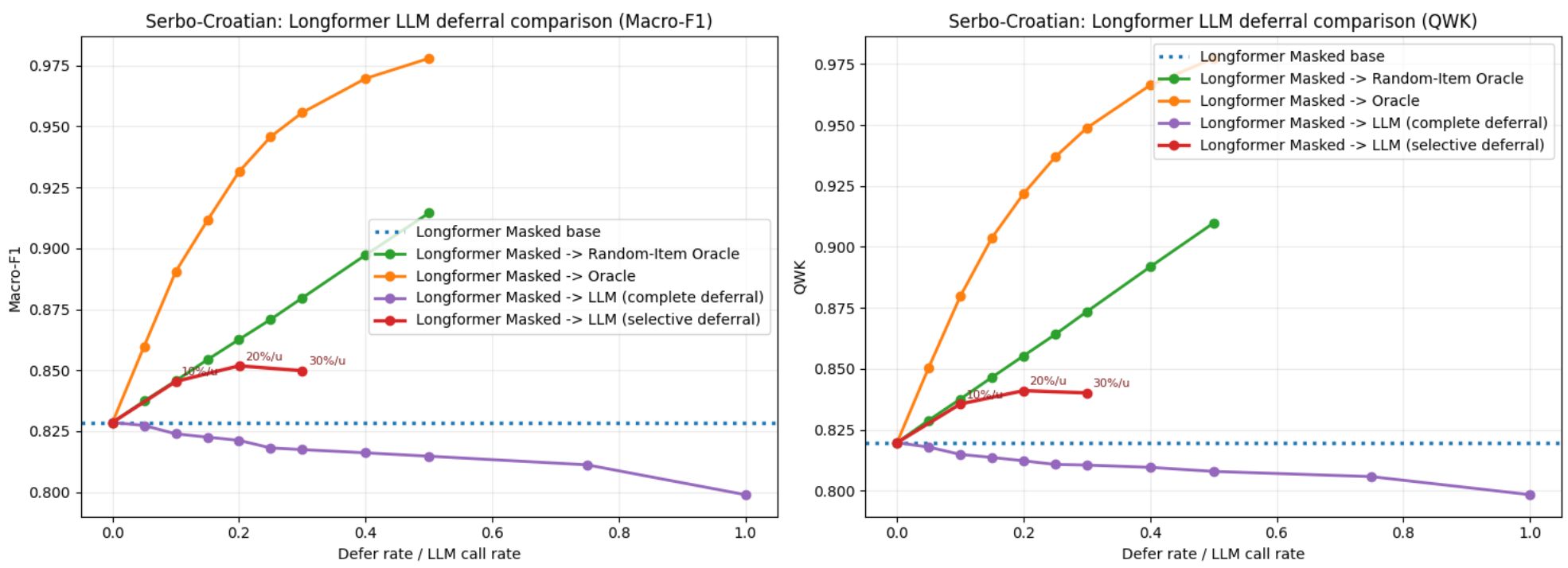


**Figure S11** Serbo-Croatian Longformer complete versus selective deferral. This provides a second Serbo-Croatian expert check for the main mDeBERTa-v3 diagnostic. The Longformer selective curve improves more clearly over the expert baseline in macro-F1 and QWK, which is consistent with the table-level finding that the Longformer-based selective row is the strongest Gemma selective-deferral row for Serbo-Croatian.

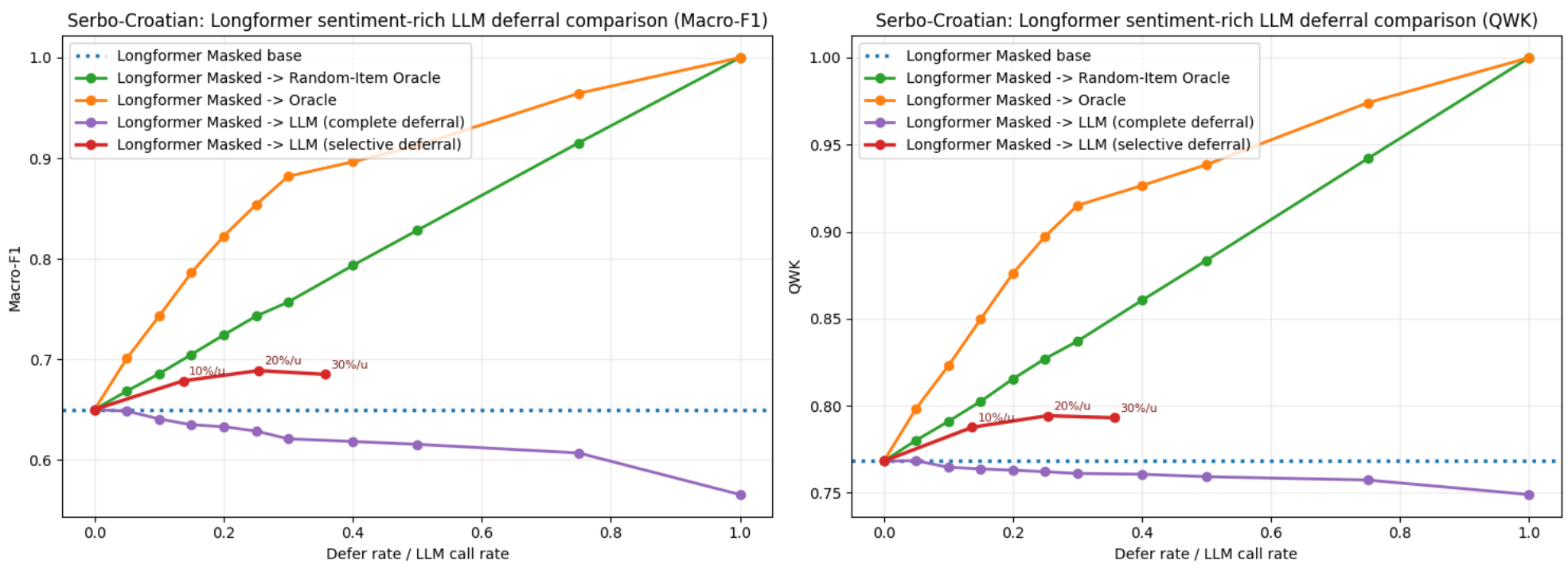


**Figure S12** Serbo-Croatian Longformer complete versus selective deferral on sentiment-bearing cases. Compared with the mDeBERTa-v3 polar subset, the Longformer curve does not taper in the same way, suggesting that the usefulness of selective deferral depends on the expert's error distribution and uncertainty ranking, not only on the language split.

The topic-level analysis is consistent with the class-conditioned view, but should be read as a descriptive aid rather than as a definitive clustering of failures. Rescue-heavy topics include telecom service and user-option contexts, product availability or promotion, crisis/SMS notifications, channel or platform listings, and financial-reporting templates. Residual both-wrong topics include bank merger or ownership articles, product-promotion articles where positive product language may not imply positive sentiment toward the target, and platform/channel listings where the target is present but not evaluated. LLM-induced errors often arise when the LLM overreacts to global affect, political or crisis language, or promotional wording. These patterns point toward local pragmatic adjudication rather than morphology-specific rescue or broad

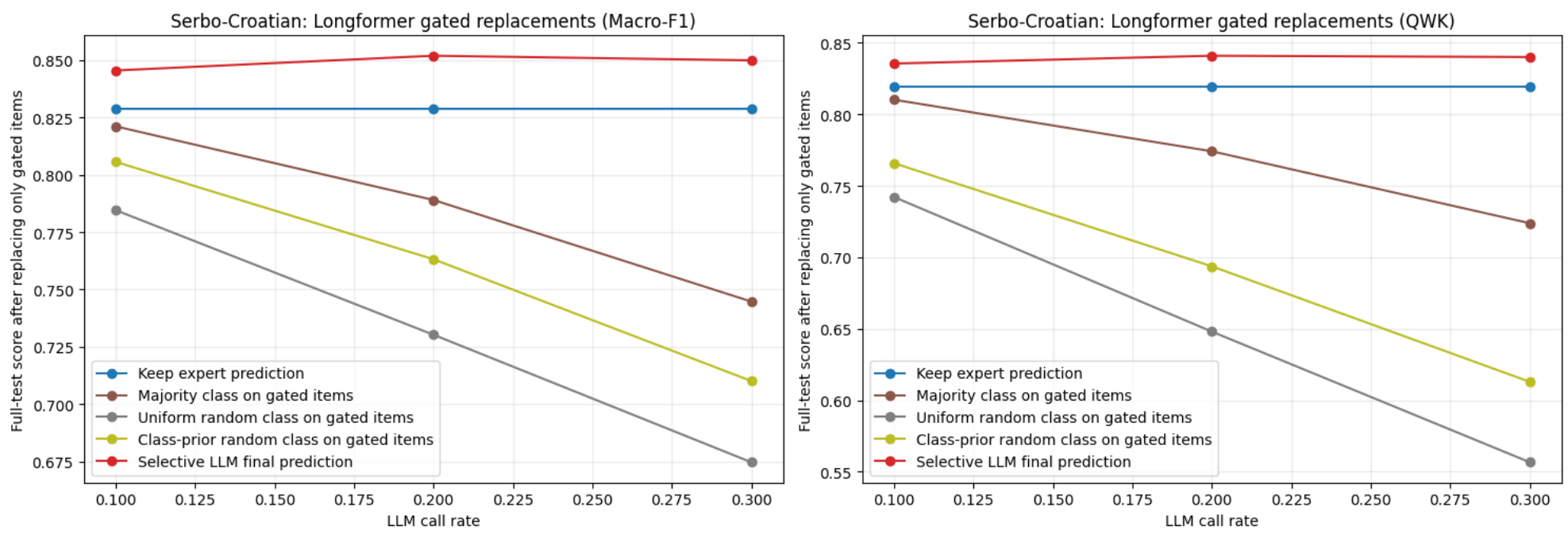


**Figure S13** Serbo-Croatian Longformer gated-item replacement scores. This supplementary robustness panel shows that selective LLM replacement can improve over keeping the expert prediction on routed hard cases for another Serbo-Croatian expert, again supporting the claim that the LLM policy is doing more than arbitrary replacement.

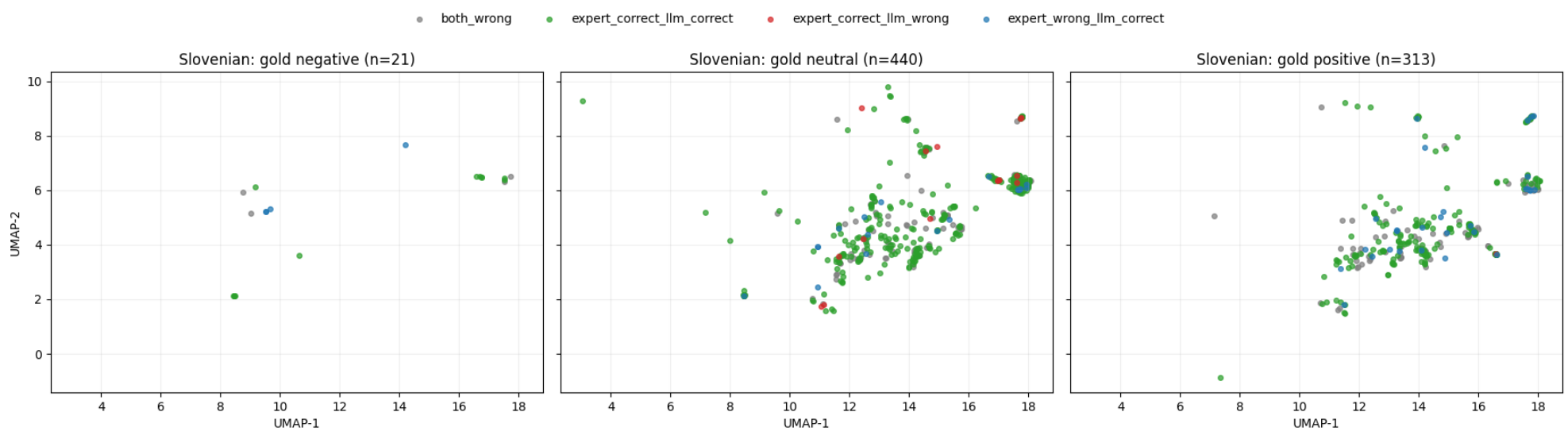


**Figure S14** Class-conditioned UMAP for Slovenian routed hard cases. The negative routed subset is small, so class-specific Slovenian claims should be treated cautiously.

external world-knowledge lookup, while the overlap across topics cautions against treating topic membership as a reliable routing rule.

Finally, the minimum viable set analysis is included as an industrial planning diagnostic rather than as a central model-comparison result. It estimates how performance, class coverage, calibration, and uncertainty change as the amount of labelled training data increases for the masked language-specific encoder setup. The progressive-data runs use 1%, 2%, 5%, 10%, 20%, 30%, 40%, 50%, 75%, and 100% training-data fractions, three run indices, validation-based early stopping, and Monte-Carlo dropout uncertainty estimates.

The main paper reports the combined performance and per-class scaling plot for panels (a) and (b). Slovenian reaches the within-2% macro-F1 band at 40% of the training data, while Serbo-Croatian reaches the within-5% macro-F1 band at 30%. These are useful planning anchors, but they are not universal stopping rules: the required data volume changes if the target is QWK, minority-class F1, calibration, or conservative variance-aware performance.

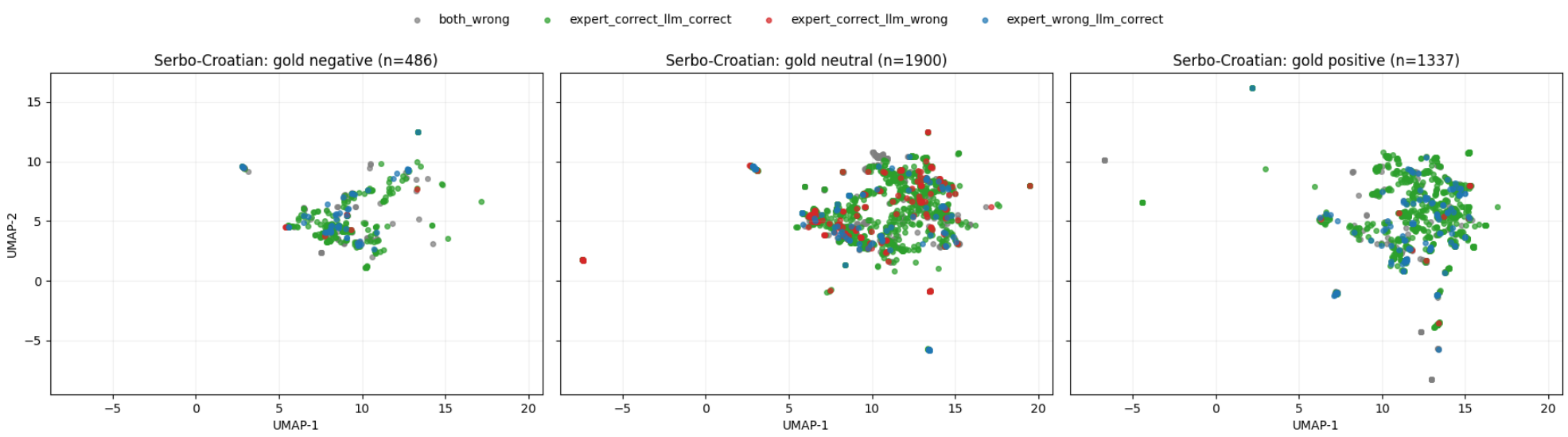


**Figure S15** Class-conditioned UMAP for Serbo-Croatian routed hard cases. Rescues are more visible on sentiment-bearing classes, while neutral cases contain more residual and LLM-induced errors.

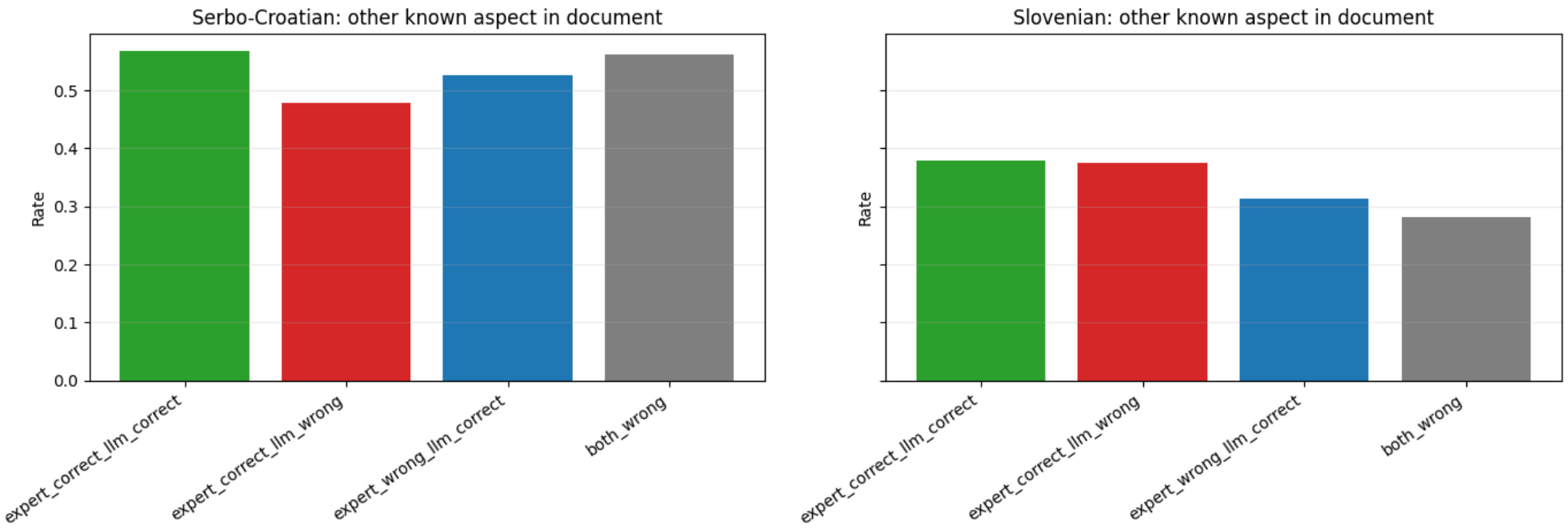


**Figure S16** Heuristic multi-aspect diagnostic based on known AspectBench target mentions. Multi-entity articles are common, but the diagnostic does not show that they are uniquely enriched among residual errors, suggesting that entity competition is one recurring failure mode rather than the dominant explanation for routed difficulty.

Panel (c) focuses on four uncertainty and calibration diagnostics. Expected Calibration Error (ECE) measures how far predicted confidence is from observed accuracy; lower ECE means a confidence score is closer to what it claims to be. Total uncertainty is the predictive entropy of the final probability distribution, so it is high when the model's predicted class distribution is spread out. Parameter uncertainty, measured through mutual information under Monte-Carlo dropout, helps us isolate uncertainty due to the model parameters, which is different from total uncertainty because it is meant to shrink as the model sees enough representative training data. The Brier score measures the squared distance between the predicted probability vector and the true one-hot label. Unlike QWK, which evaluates the final hard label and penalises ordinal sentiment mistakes, Brier score evaluates the quality of the predicted probabilities themselves.

Panel (d) compares several MVS detection methods. Relative macro-F1 thresholds ask how much data is needed to come within 10%, 5%, or 2% of the full-data macro-F1 score. The geometric knee marks the point where the scaling curve bends from rapid early gains into slower diminishing returns; it is useful for identifying a small pilot

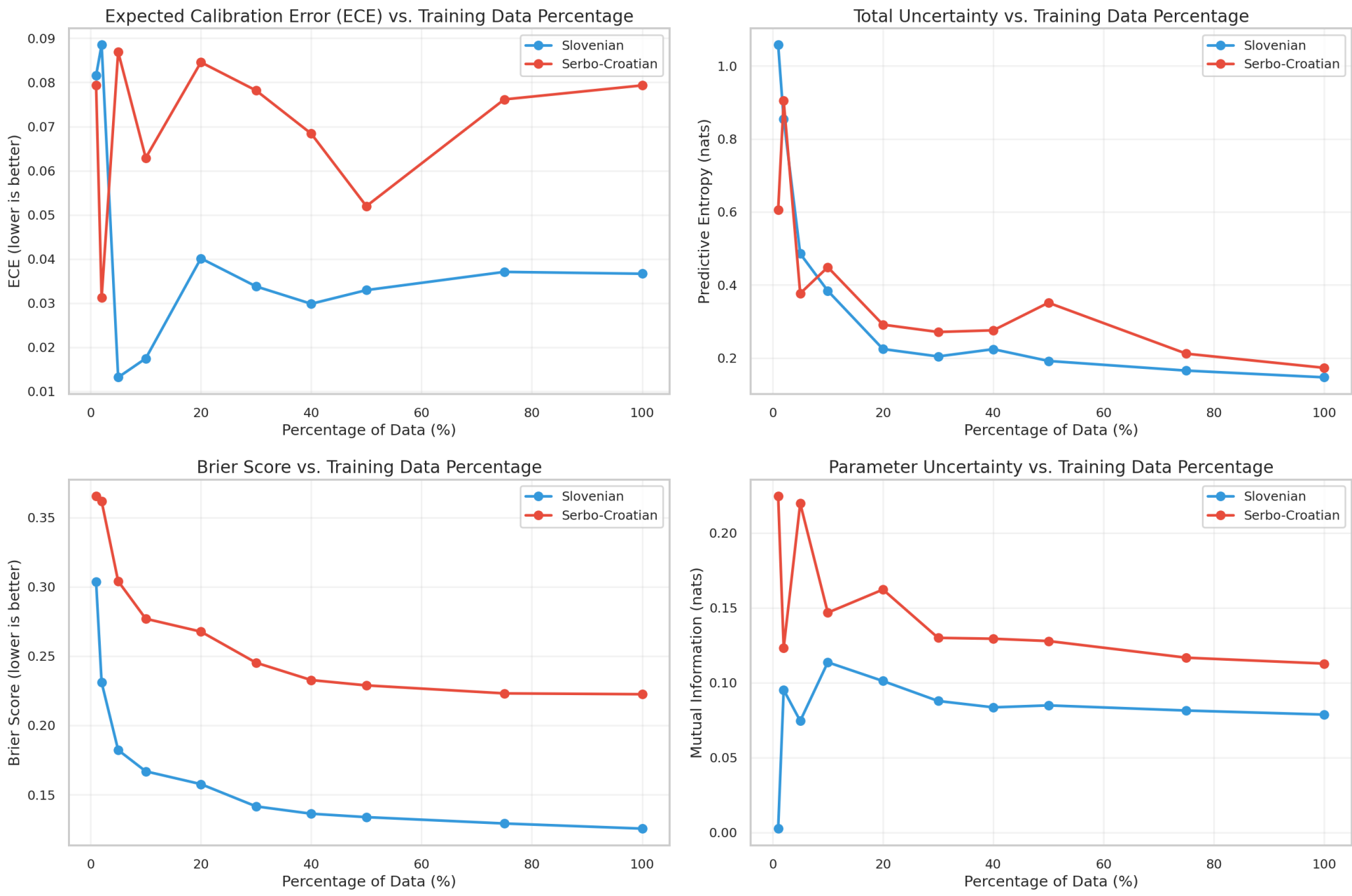


**Figure S17** Minimum viable set analysis: calibration, total uncertainty, Brier score, and parameter uncertainty across training-data percentages. Total uncertainty and Brier score generally decrease as more data is added, while ECE and parameter uncertainty are less monotonic. These curves are therefore best read as planning diagnostics for uncertainty-aware deployment rather than as a single stopping rule.

size, but it is not the same as final-quality performance. The statistical one-standard-error rule selects the smallest data fraction whose performance is within one standard error of the best observed score, making it deliberately conservative. The epistemic plateau identifies where parameter uncertainty stops decreasing meaningfully under a 10% tolerance. The wide spread across these criteria is the main result: an annotation campaign should choose the stopping rule that matches its operational goal.

# S5 Model Hyperparameters and Training Details

This section summarises the hyperparameters and key configuration details for the models evaluated in this study. For supervised encoder models, the reported checkpoint is selected by validation macro-F1 and evaluated on the fixed holdout test split. Run-level details for the LLM deferral experiments are retained in the experiment metadata used to generate the reported tables. Tables S2 and S3 report the main training and inference settings.

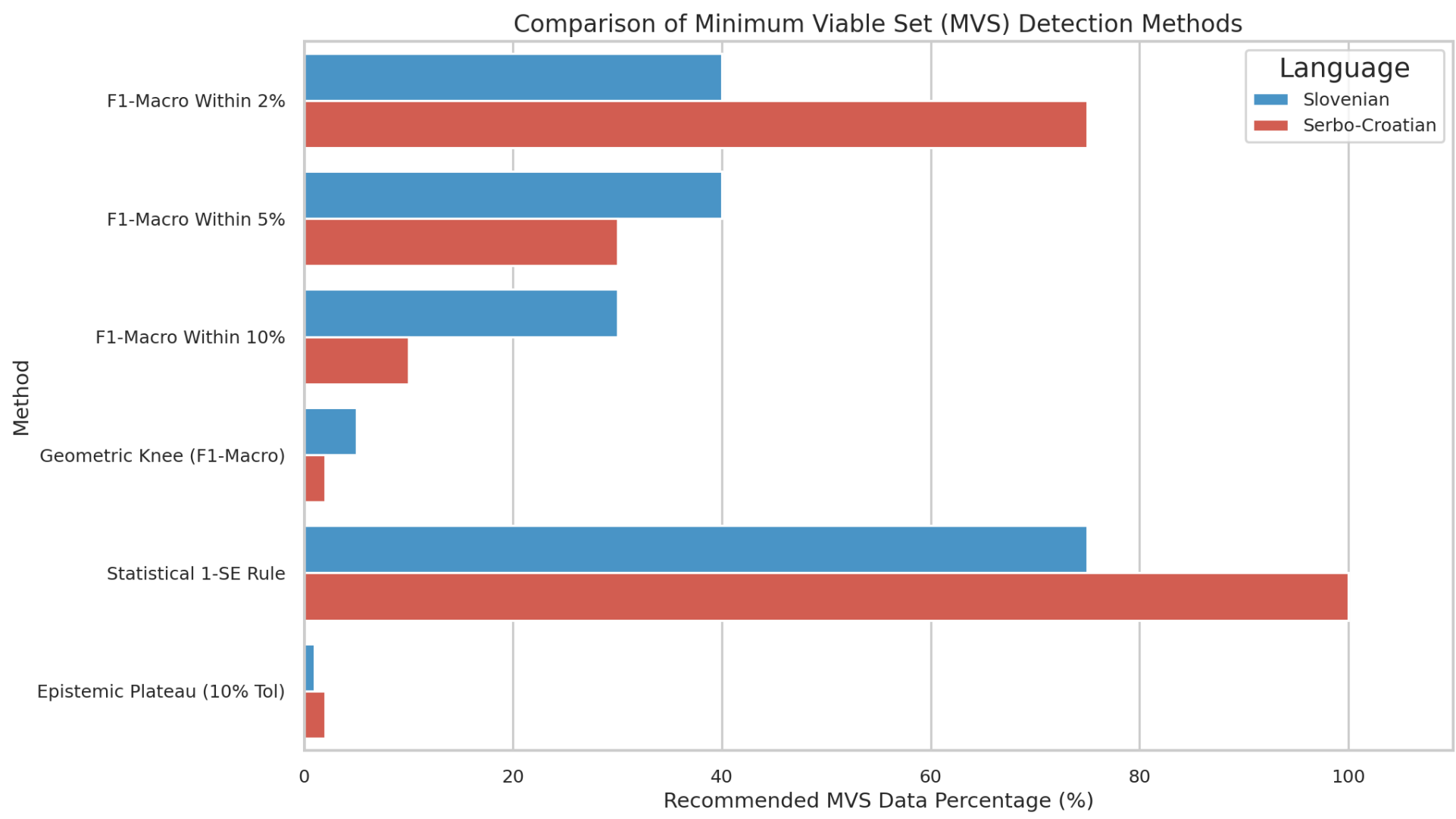


**Figure S18** Minimum viable set analysis: comparison of detection criteria. Slovenian reaches the within-2% macro-F1 band at 40%, while Serbo-Croatian reaches the within-5% macro-F1 band at 30%, but conservative one-standard-error thresholds require substantially more data. The estimated minimum viable set is therefore criterion-dependent and should be treated as an annotation-planning lens rather than as a universal threshold.

| Model Family | Setting | Value |
|---|---|---|
| **Embeddings** | Base model | BGE-M3 (‘BAAI/bge-m3’), 1024-dimensional document embeddings |
| | Classifier | MLP classifier (1024 → 512 → 256 → 3), ReLU activations, dropout 0.3 |
| | Optimization | AdamW, learning rate 1e-4, batch size 64, weight decay 0.01, 15 epochs, validation macro-F1 checkpoint selection |
| **XLM-RoBERTa** | Base model and inputs | ‘xlm-roberta-base’; article-aspect paired input, maximum length 512 |
| | Variants | Truncated full article, extractive aspect-sentence filtering, LLM summary, and masked LLM summary |
| | Optimisation | AdamW, learning rate 2e-5, batch size 32, 10 epochs, validation macro-F1 checkpoint selection |
| **Longformer** | Base model and inputs | ‘markussagen/xlm-roberta-longformer-base-4096’; article-aspect paired input, maximum length 4096, global attention assigned to the first token |
| | Optimisation | AdamW, learning rate 1e-5, batch size 2, gradient accumulation over 16 steps (effective batch size 32), 10 epochs, gradient checkpointing |
| **mDeBERTa-v3** | Base model and inputs | ‘microsoft/mdeberta-v3-base’; article-aspect paired input, maximum length 512 |
| | Optimisation | AdamW, learning rate 1e-5, batch size 8, gradient accumulation over 4 steps (effective batch size 32), 10 epochs, full precision |
| **mT5** | Base model and inputs | ‘google/mt5-base’; source text contains task prefix, aspect, and article; maximum source length 512, maximum target length 8 |
| | Label decision | Class label selected by minimum conditional generation loss over {negative, neutral, positive} |
| | Optimisation | AdamW, learning rate 3e-5, batch size 4, gradient accumulation over 8 steps (effective batch size 32), 10 epochs, gradient checkpointing, full precision |
| **SloBERTa / BERTić** | Base models and inputs | Slovenian: ‘EMBEDDIA/sloberta’; Serbo-Croatian/HBS: ‘classla/bcms-bertic’; article-aspect paired input, maximum length 512 |
| | Optimisation | AdamW, learning rate 2e-5, batch size 16, gradient accumulation over 2 steps (effective batch size 32), 10 epochs, full precision |
| **HAN-XLMR** | Document structure | XLM-R sentence encoder, maximum sentence length 96, maximum 128 sentences per document |
| | Architecture | Two-layer sentence-interaction transformer; 8 interaction heads; 4 aggregation attention heads; final MLP hidden size 256; dropout 0.2 |
| | Optimisation | AdamW, learning rate 1e-5, micro-batch size 4 with gradient accumulation to effective batch size 32, 10 epochs |

**Table S2** Hyperparameter settings for embedding, encoder, sequence-to-sequence, and hierarchical neural baselines.

| Model Family | Setting | Value |
|---|---|---|
| **LLM Prompting** | Student and teacher models | Inference model: Gemma-3-27b-QAT; DSPy optimisation teacher: Qwen-2.5-72b |
| | Decoding | Three independent queries per item; majority-vote prediction; temperature 1.0, top-k 64, top-p 0.95, maximum generation length 1024 tokens |
| | DSPy optimisation | MIPROv2 over balanced train/validation subsets; direct prompting, DSPy direct prompting, and DSPy-CoT variants |
| **Learning to Defer** | Expert policies | Majority vote, probability averaging, confidence selection, logistic-regression stacking, and random-forest stacking |
| | Expert sets | Slovenian: Longformer, SloBERTa, HAN-XLMR; Serbo-Croatian: Longformer, mDeBERTa-v3, BERTić, HAN-XLMR |
| **Complete Deferral** | Evidence designs | Softmax-evidence and uncertainty-evidence deferral; all instances are sent to the LLM together with the expert prediction and supporting confidence evidence |
| | DSPy optimisation budget | Slovenian HAN softmax/uncertainty rows: heavy; Serbo-Croatian HAN softmax unmasked: medium; Serbo-Croatian HAN softmax masked: heavy; Serbo-Croatian HAN uncertainty rows: medium; expanded top-expert complete rows: medium except Serbo-Croatian Longformer masked complete deferral: heavy |
| **Selective Deferral** | Routing policy | Instances ranked by expert confidence; only the lowest-confidence fixed fraction is routed to Gemma-3-27b-QAT, while high-confidence instances keep the expert prediction |
| | Gate sweep and budget | Gate rates 0.10, 0.20, and 0.30; all selected selective-deferral runs use medium-budget DSPy optimisation |
| | Main selected points | Slovenian Longformer: 0.10 gate with unmasked LLM prompt; Serbo-Croatian mDeBERTa-v3: 0.30 gate with unmasked LLM prompt; Serbo-Croatian Longformer: 0.20 gate as supplementary robustness check |
| **Controls** | Baselines | Zero-shot document sentiment classifier and majority-class classifier |

**Table S3** Hyperparameter and inference settings for LLM prompting, learning-to-defer, deferral, and control models.

*Note.* Medium and heavy refer to DSPy MIPROv2 optimisation budgets defined in the experiment configuration. Full run-level settings are preserved in the experiment metadata used to generate the reported result tables.

# References

[1] Joulin, A., et al.: Fasttext.zip: Compressing text classification models. arXiv preprint (2016) 1612.03651

[2] Joulin, A., *et al.*: Bag of tricks for efficient text classification. In: Proc. 15th Eur. ACL Short Papers, pp. 427–431 (2017)
